# Supplementary material for: Olaparib, durvalumab, and cyclophosphamide, and a prognostic blood signature in platinum-sensitive ovarian cancer: the randomized phase 2 SOLACE2 trial
Source: Nat Commun. 2025 Nov 5;16:9756. doi: 10.1038/s41467-025-64130-6 (PMC12589412; doi:10.1038/s41467-025-64130-6)
Supplement: Supplementary file 1 — Supplementary Information [file 41467_2025_64130_MOESM1_ESM.pdf]

Supplementary note 1

Supplementary Figure 1

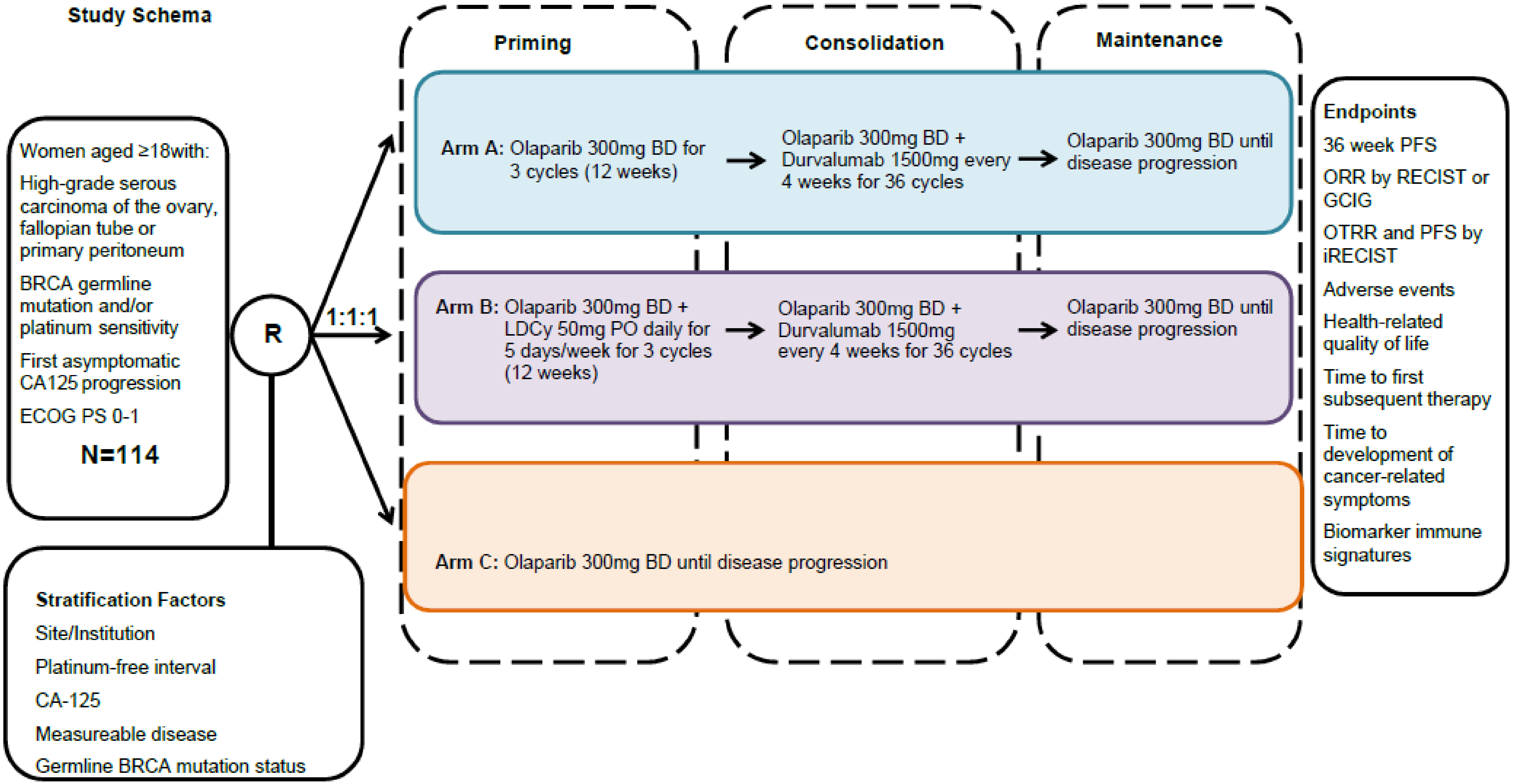

Study schema for the SOLACE2 trial
BRCA, breast cancer gene; ECOG PS, Eastern Cooperative Oncology Group Performance Status; BD, twice daily; PO, by mouth; PFS, progression free survival; ORR, Overall response rate; RECIST, Response Evaluation Criteria in Solid Tumors; GCIG, Gynecologic Cancer InterGroup CA125 criteria; OTRR, Overall Tumor Response Rate; iRECIST, Immune-related Response Evaluation Criteria in Solid Tumors;

Supplementary Figure 2

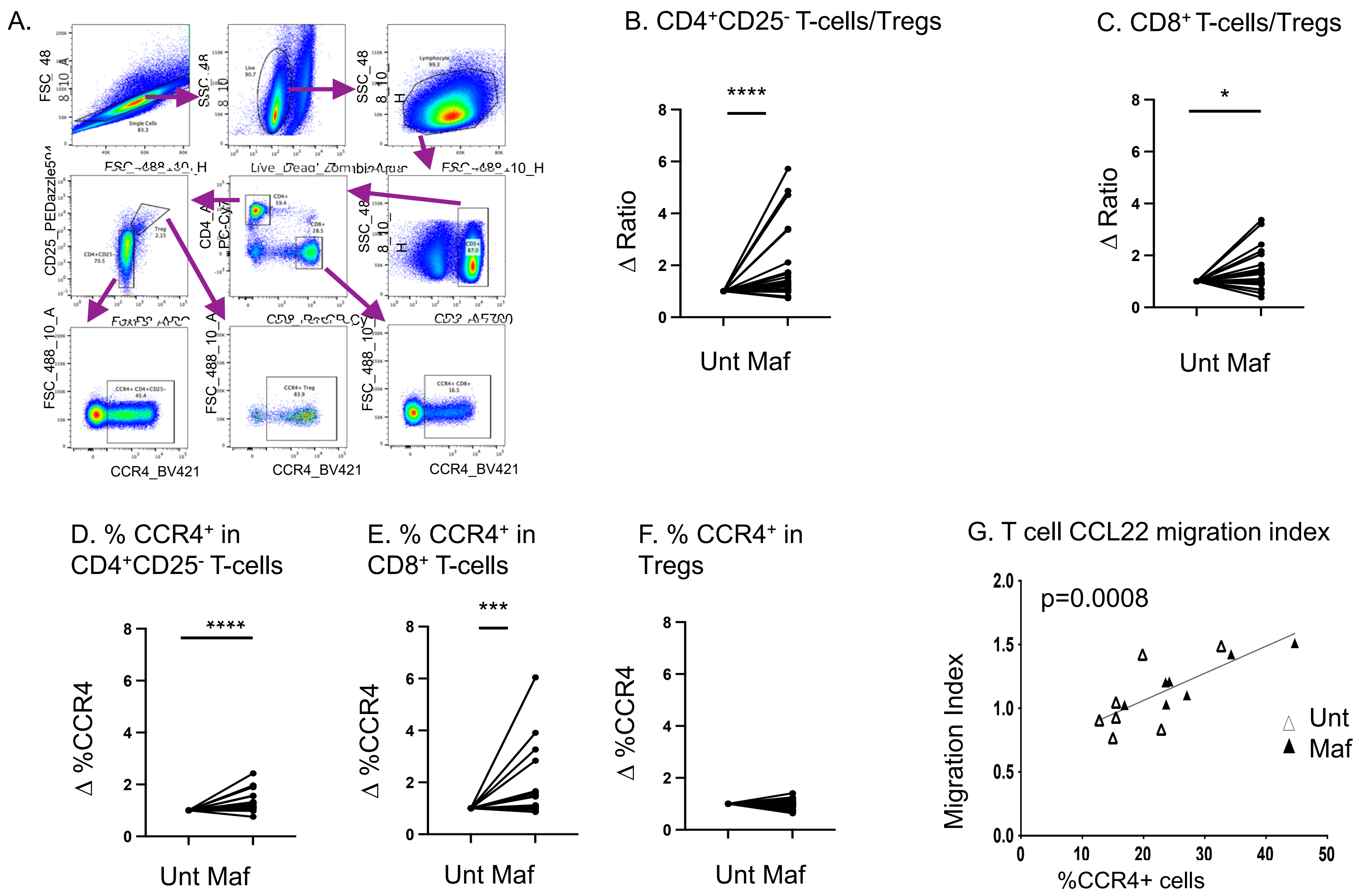

Mafosfamide increases the CD4$_+$CD25$_-$ T cells/Treg and CD8$_+$ T cells/Treg ratio and the functional expression of CCR4 on the surface of CD4$_+$CD25$_-$ T cells and CD8$_+$ T cells, but not Treg in healthy donors. Mafosfamide effects were analyzed on post co-culture for 72 hours with human peripheral blood mononuclear cells from healthy blood donors (N=26). (A) Flow cytometry gating strategy to characterize T-cell subsets and their CCR4 expression. The ratios of (B) CD4 +CD25- T cells/Treg and (C) CD8+ T cells/Treg were increased following 72 hours incubation with mafosfamide in comparison to the untreated controls. The proportion of CCR4+cells within both (D) CD4+CD25- T cells and (E) CD8+ T cells were increased, while in the (F) Treg population they remained unchanged.

Non-parametric paired test Wilcoxon matched-pairs signed-rank test. **** P<0.0001, *** P<0.001, ** P<0.01 and * P<0.05. Unt, Untreated; Maf, Mafosfamide

Supplementary Figure 3

A

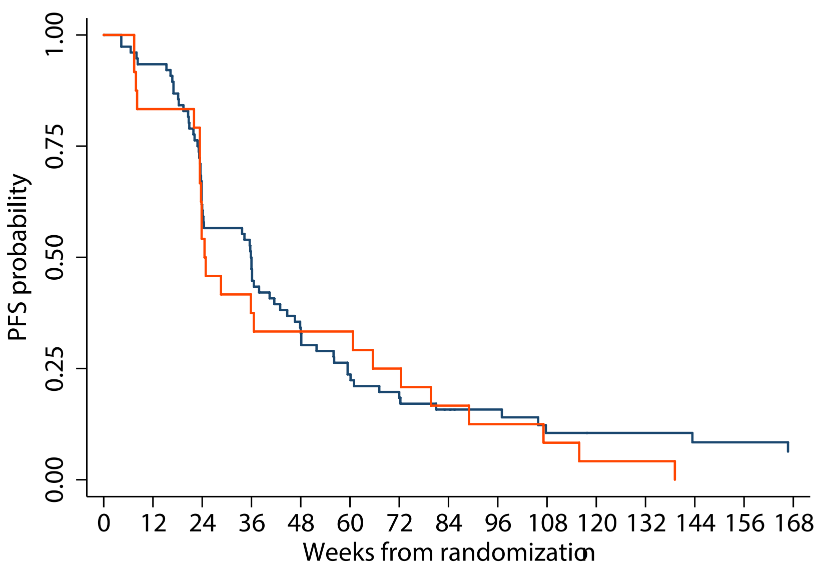

Number at risk															
CD4+CD25−T cell/Treg≤270	76	71	47	38	26	18	15	11	9	6	5	5	4	4	3
CD4+CD25−T cell/Treg>270	24	20	13	9	8	8	6	4	3	2	1	1	0	0	0

CD4+CD25−T cell/Treg≤270 CD4+CD25− T cell/Treg>270

B

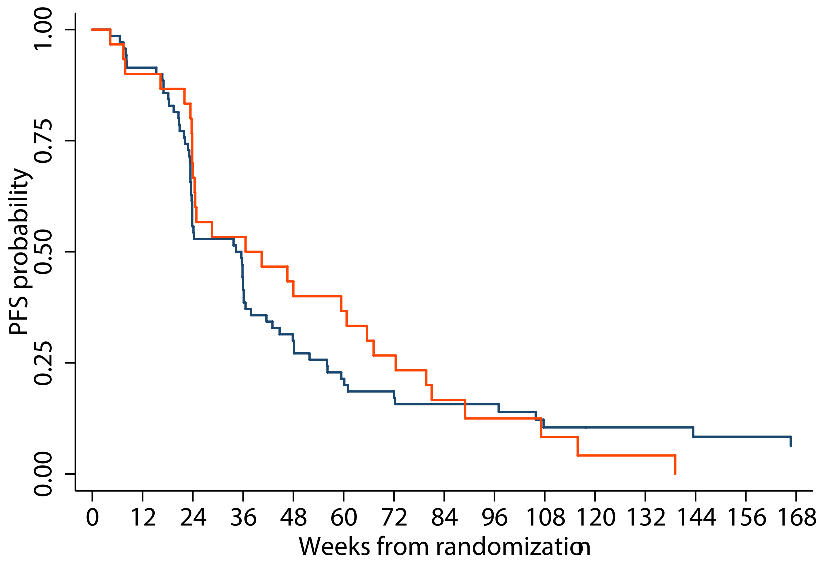

Number at risk															
CD8 T cell/Treg≤40	70	64	39	31	21	15	13	10	9	6	5	5	4	4	3
CD8 T cell/Treg>40	30	27	21	16	13	11	8	5	3	2	1	1	0	0	0

CD8 T cell/Treg≤40 CD8 T cell/Treg>40

Kaplan-Meier plot of progression-free survival based on (A) CD4+CD25- T cell/ Treg and (B) CD8 T cell/Treg following mafosfamide stimulation

PFS, Progression Free Survival;

Supplementary Figure 4

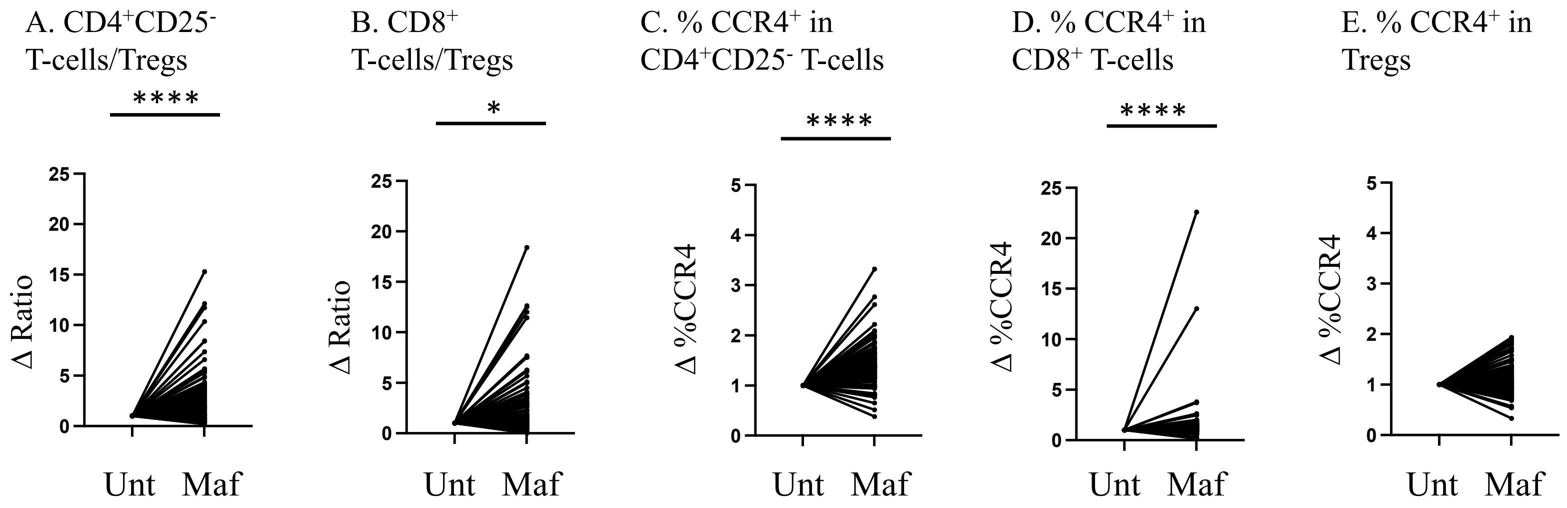

Mafosfamide increases the CD4$^+$CD25$^-$ T cells/Treg and CD8$^+$ T cells/Treg ratio and the expression of CCR4 on the surface of CD4+CD25- T cells and CD8+ T cells, but not Treg in SOLACE2 participants. T-cell subsets and percentages of CCR4+ expressing cells were measured following 72 hours incubation with or withoutmafosfamide, using flow cytometry, in SOLACE2 participants (N=106). Ratios of (A) CD4+CD25- T cells/Treg and (B) CD8+ T cells/Treg increased following 72 hours of mafosfamide culture. Mafosfamide increased the proportion of CCR4+ cells within (C) CD4+CD25- T cells, (D) CD8+ T cells, but not (E) Treg. Non-parametric paired test Wilcoxon matched-pairs signed-rank test. **** P<0.0001, *** P<0.001, ** P<0.01 * P<0.05.

Unt, Untreated; Maf, Mafosfamide

Supplementary Figure 5

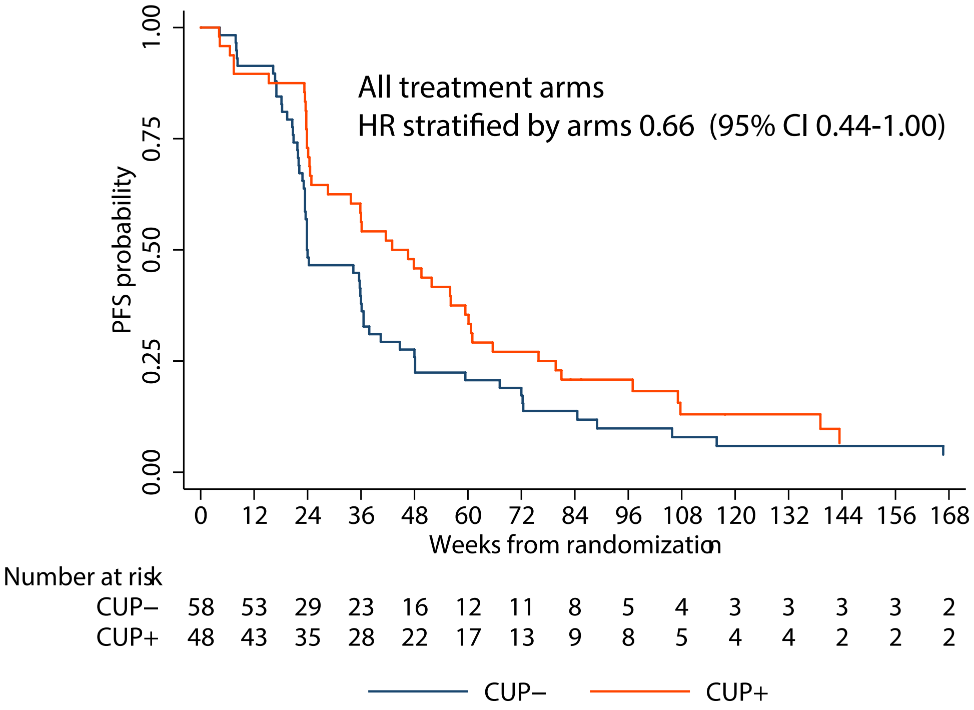

Kaplan-Meier plot of progression-free survival based on CCR4 upregulation status in CD4+CD25- T cells and CD8+ T cells (CUP)
PFS, Progression Free Survival;HR, Hazard Ratio; CI, Confidence interval; CUP, CCR4 upregulation;

Supplementary Figure 6

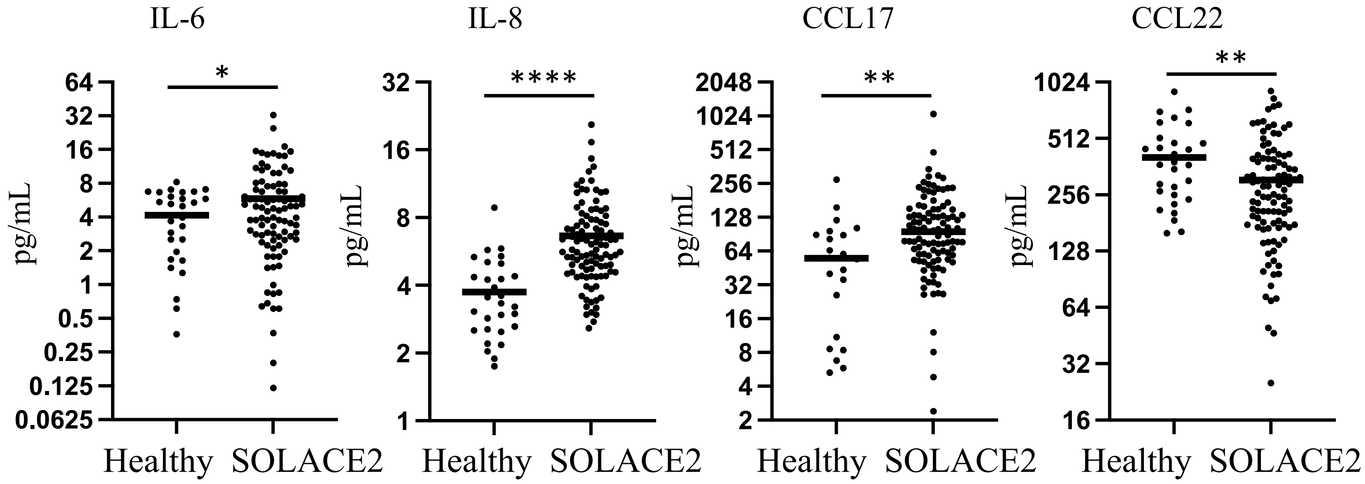

Pro-inflammatory cytokine (IL-6 and IL-8) and CCR4 chemokine ligands (CCL17 and CCL22) levels in healthyblood donors and SOLACE2 participants. The circulating pro-inflammatory cytokines, IL-6 and IL-8 were higher in SOLACE2 participants (N=105) compared to healthy blood donors (N=29). The CCL17 ligand was also higher, but circulating CCL22 ligand was lower in SOLACE2 participants compared to the healthy blood donors. The Mann-Whitney tests were used to compare the circulating cytokines/ chemokines of healthy to cancer patients. **** is P<0.0001, *** is P<0.001, ** is P<0.01 and * is P<0.05. IL, interleukin

Supplementary Figure 7

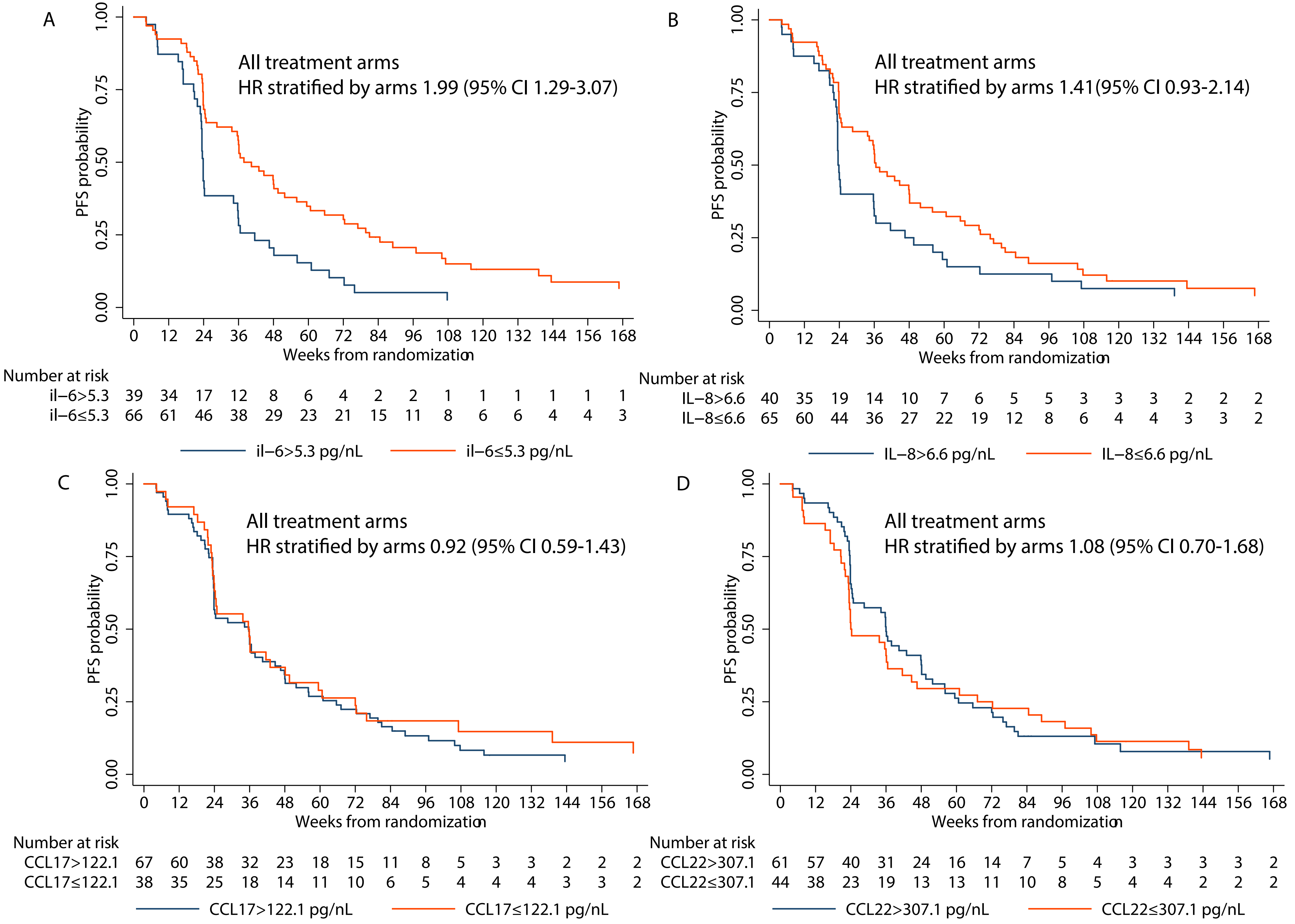

Kaplan-Meier plot of progression-free survival based on baseline serum levels of (A) Interleukin-6, (B) Interleukin-8, (C) CCL17 and (D) CCL22 PFS, Progression Free Survival;HR, Hazard Ratio; CI, Confidence interval; CCL, chemokine ligand;

Supplementary Figure 8

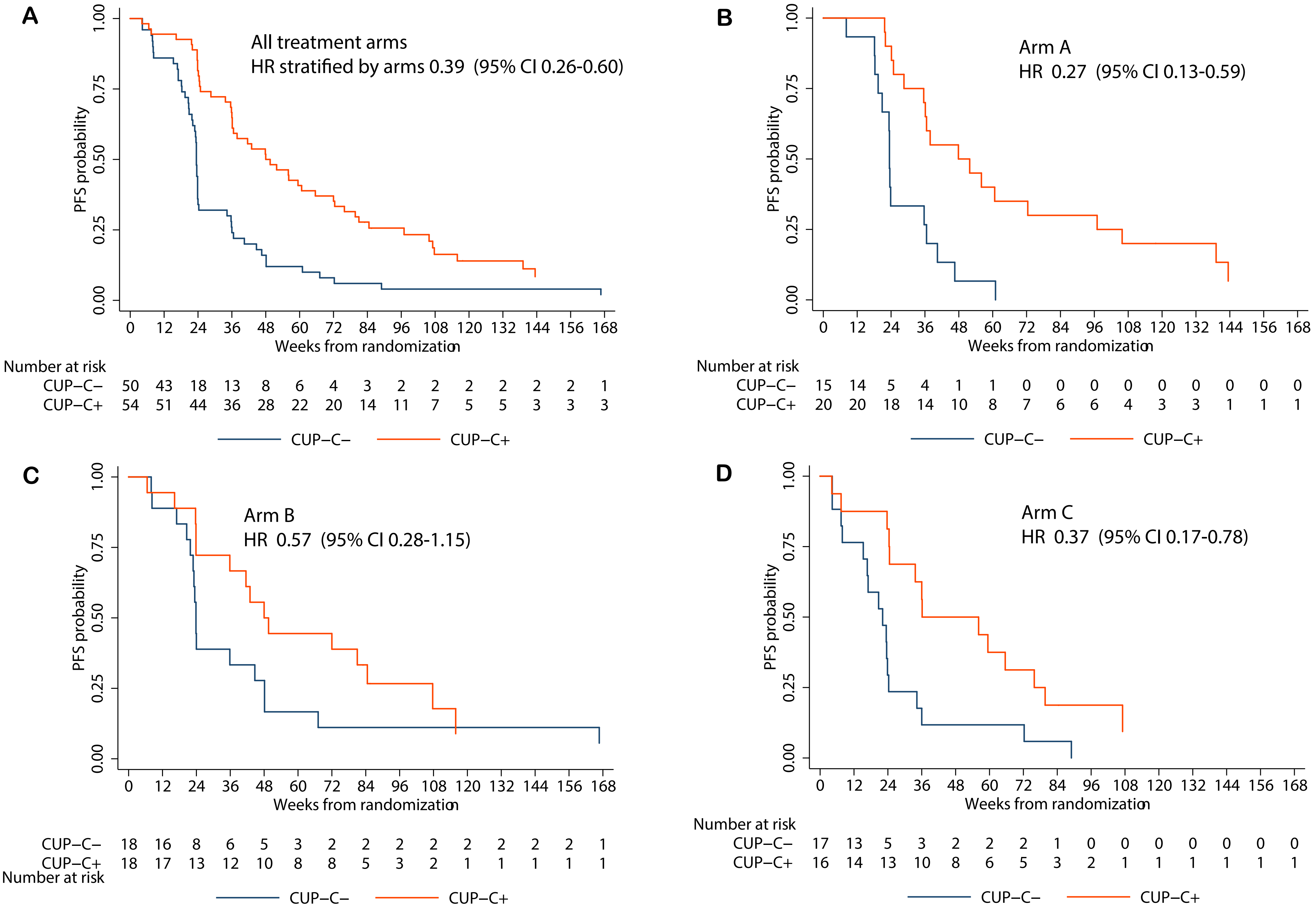

Kaplan-Meier plot of prognostic significance of CUP-C. PFS (A) in all patients and (B-D) by treatment arms

PFS, Progression Free Survival;HR, Hazard Ratio; CI, Confidence interval; CUP-C, CCR4 up-regulation and cytokines;

Supplementary Figure 9

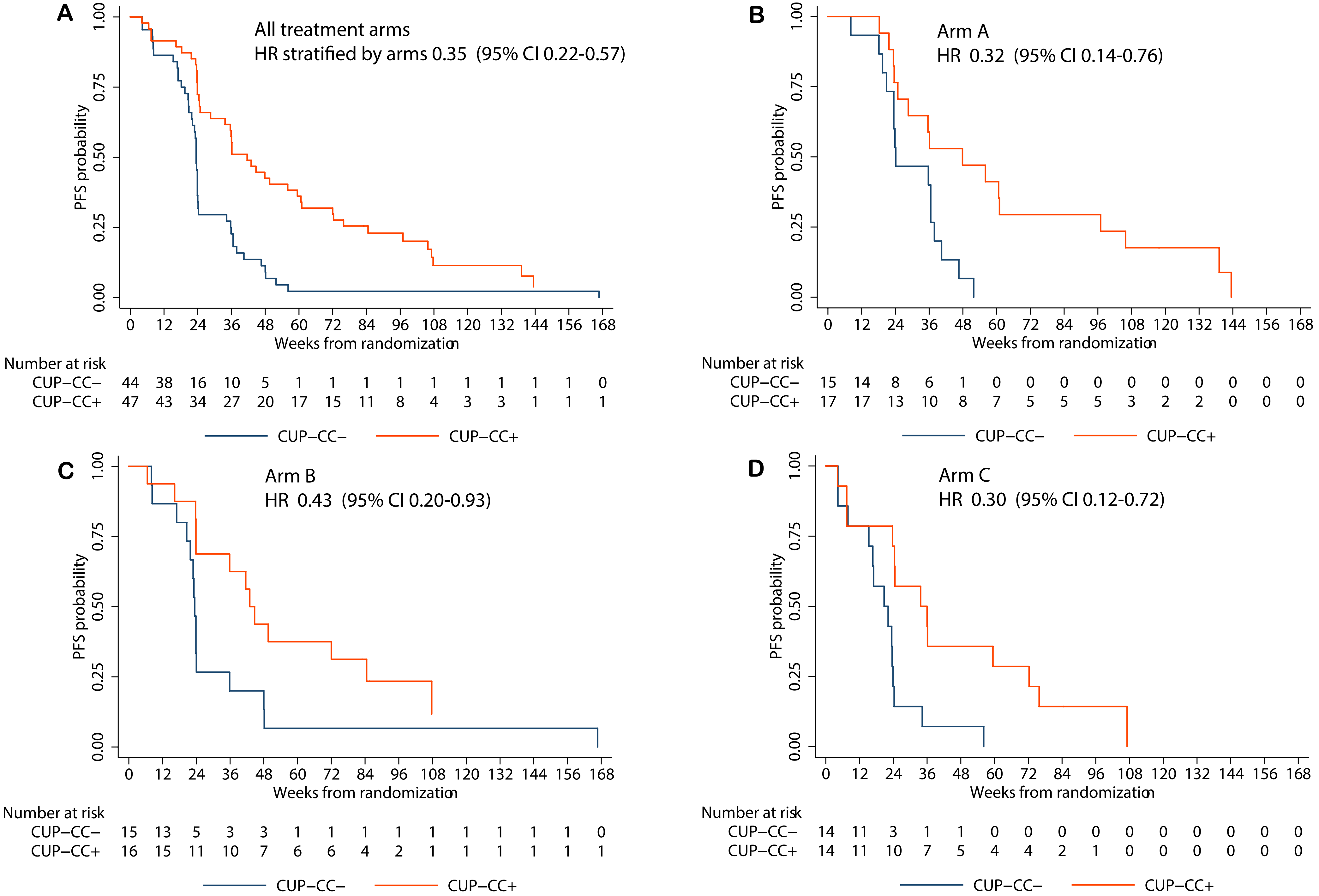

Prognostic significance of CUP-CC in all BRCA wild-type population only (A) All treatment arms and (B-D) by treatment arm
PFS, Progression Free Survival;HR, Hazard Ratio; CI, Confidence interval; CUP-CC, CCR4 up-regulation, cytokines and chemokines;

Supplementary Figure 10

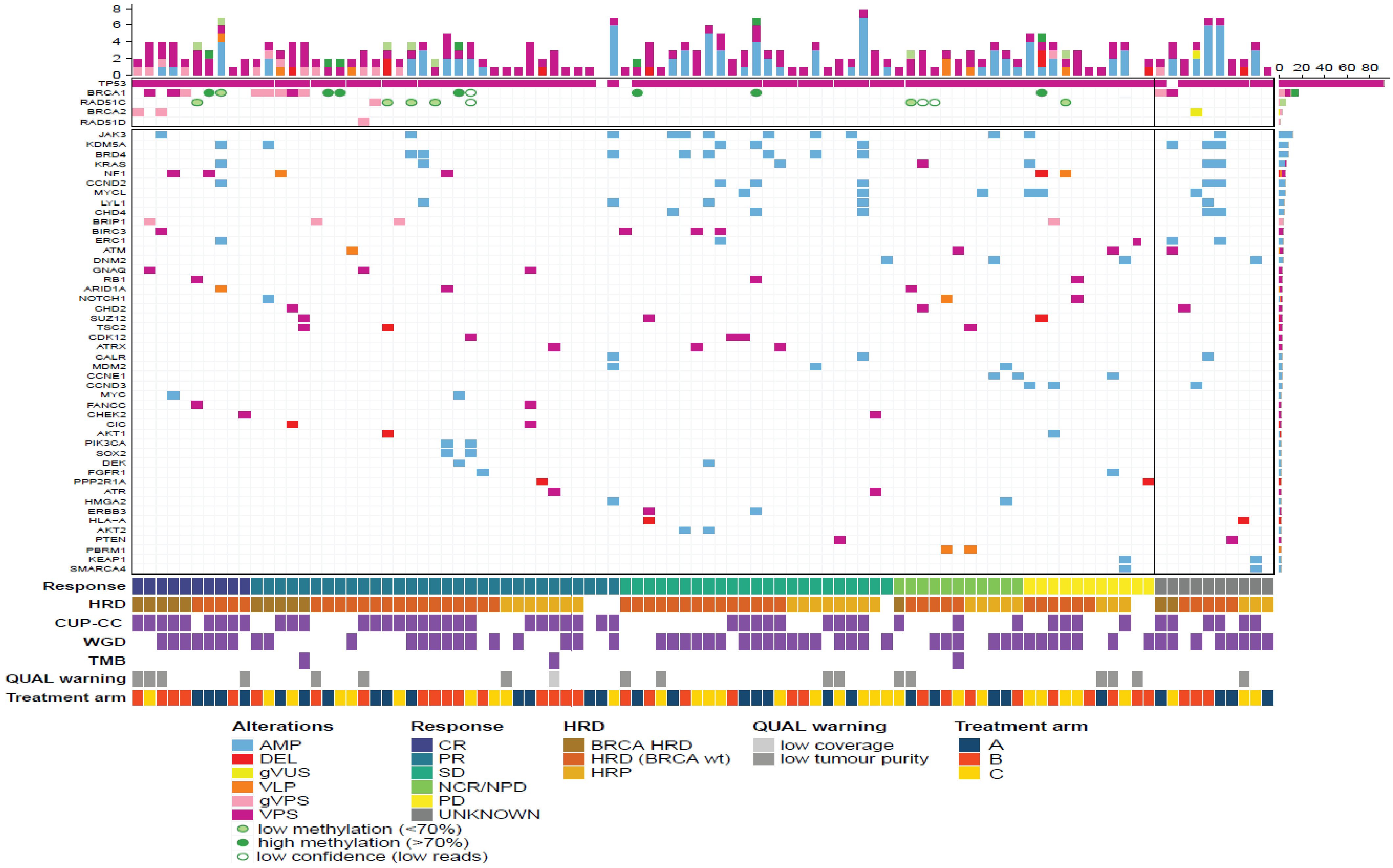

Oncoprint - Whole Exome Sequencing analysis
(TWIST Exome 2.0 with 1Mb SNP backbone and IDT UMI for single molecule normalization) HRD status determined by Genomic Instability Score, mutation status and manual review

Supplementary Figure 11

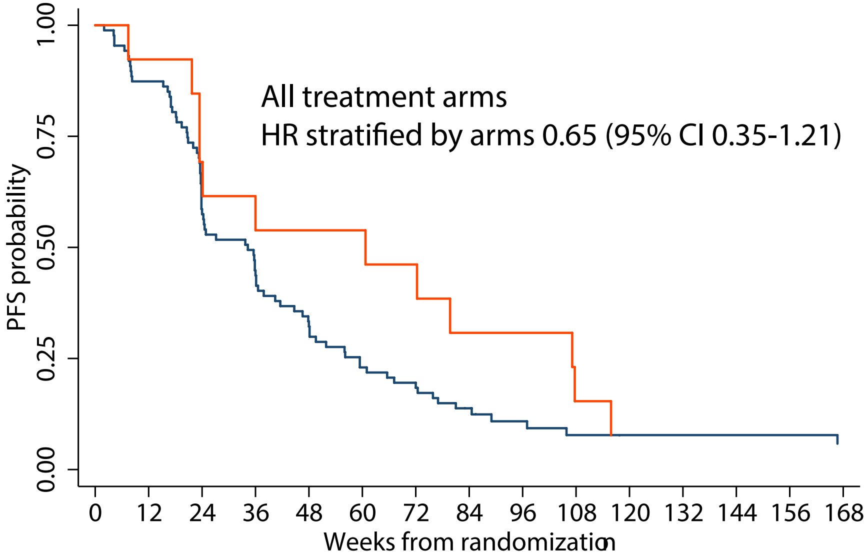

Number at risk

TMB≤5	87	76	51	39	29	20	17	11	7	5	4	4	4	4	3
TMB>5	13	12	9	8	7	7	6	4	4	2	1	1	1	1	1

TMB ≤5 mut/megabase

TMB>5 mut/megabase

Kaplan-Meier plot of progression-free survival based on tumor mutation burden PFS, Progression Free Survival;HR, Hazard Ratio; CI, Confidence interval; TMB, Tumor mutation burden;

Supplementary Figure 12

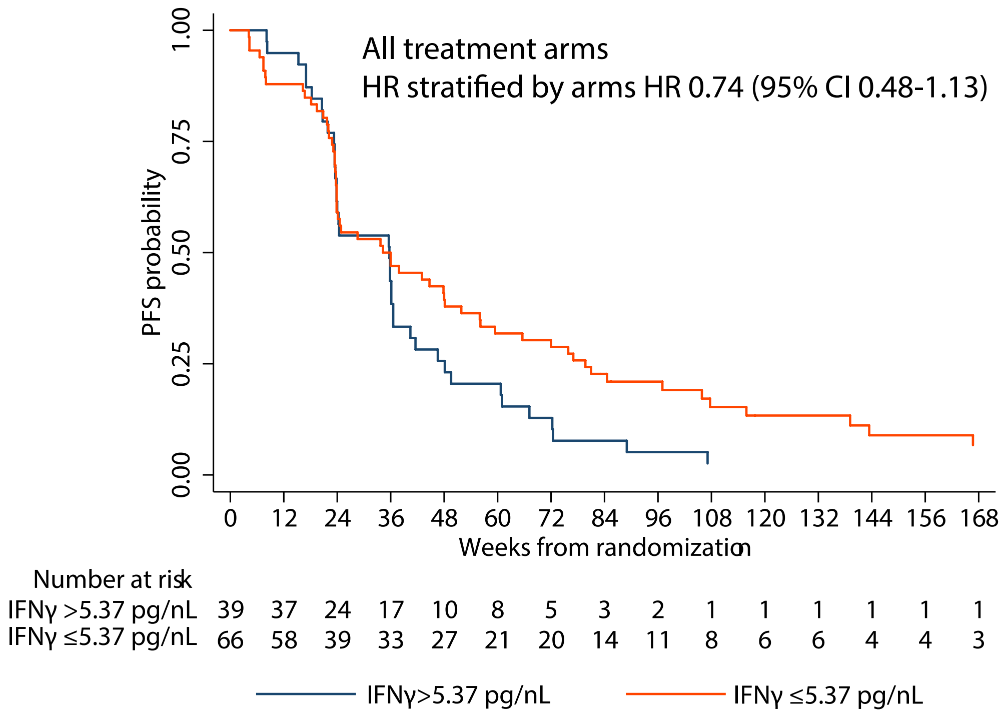

Kaplan-Meier plot of progression-free survival based on baseline level of IFN-γ PFS, Progression Free Survival; HR, Hazard Ratio; CI, Confidence interval; IFN-γ, interferon gamma;

Supplementary Table 1
Detailed adverse event listing by treatment arms
AE, adverse event;

Table 1A: Priming Phase	ARM A (N = 38)		ARM B (N = 39)		ARM C (N = 37)	
AE Term	Any Grade	Grade 3+	Any Grade	Grade 3+	Any Grade	Grade 3+
Nausea	23 (60.5%)	0	30 (76.9%)	0	26 (70.3%)	0
Fatigue	18 (47.4%)	0	19 (48.7%)	1 (2.6%)	16 (43.2%)	0
Anemia	6 (15.8%)	3 (7.9%)	15 (38.5%)	9 (23.1%)	8 (21.6%)	2 (5.4%)
Diarrhea	5 (13.2%)	0	6 (15.4%)	0	9 (24.3%)	0
Constipation	6 (15.8%)	0	5 (12.8%)	0	5 (13.5%)	0
Dysgeusia	5 (13.2%)	0	4 (10.3%)	0	7 (18.9%)	0
Vomiting	4 (10.5%)	1 (2.6%)	6 (15.4%)	0	6 (16.2%)	0
Anorexia	2 (5.3%)	0	6 (15.4%)	0	4 (10.8%)	0
Neutrophil Count Decreased	0	0	10 (25.6%)	7 (17.9%)	2 (5.4%)	0
Dyspnea	3 (7.9%)	0	1 (2.6%)	0	7 (18.9%)	0
Abdominal Pain	2 (5.3%)	0	2 (5.1%)	0	6 (16.2%)	0
Creatinine Increased	4 (10.5%)	0	2 (5.1%)	0	2 (5.4%)	0
Gastroesophageal Reflux Disease	2 (5.3%)	0	2 (5.1%)	0	3 (8.1%)	0
Rash Maculo-Papular	2 (5.3%)	0	0	0	5 (13.5%)	0
Alopecia	0	0	5 (12.8%)	0	0	0
Insomnia	2 (5.3%)	0	2 (5.1%)	0	1 (2.7%)	0
Mucositis Oral	2 (5.3%)	0	3 (7.7%)	0	0	0
Pain	1 (2.6%)	0	3 (7.7%)	0	1 (2.7%)	0
Back Pain	2 (5.3%)	0	0	0	2 (5.4%)	0
Bloating	2 (5.3%)	0	1 (2.6%)	0	1 (2.7%)	0
Cough	1 (2.6%)	0	2 (5.1%)	1 (2.6%)	1 (2.7%)	0
Dyspepsia	0	0	3 (7.7%)	0	1 (2.7%)	0
Edema Limbs	0	0	1 (2.6%)	0	3 (8.1%)	0
Headache	1 (2.6%)	0	1 (2.6%)	0	2 (5.4%)	0
Urinary Tract Infection	1 (2.6%)	0	2 (5.1%)	1 (2.6%)	1 (2.7%)	0
Dizziness	0	0	3 (7.7%)	1 (2.6%)	0	0
Dysuria	1 (2.6%)	0	1 (2.6%)	0	1 (2.7%)	0
Lethargy	1 (2.6%)	0	1 (2.6%)	0	1 (2.7%)	0
Lymphocyte Count Decreased	1 (2.6%)	0	2 (5.1%)	2 (5.1%)	0	0
Muscle Cramp	2 (5.3%)	0	0	0	1 (2.7%)	0
Platelet Count Decreased	0	0	2 (5.1%)	1 (2.6%)	1 (2.7%)	1 (2.7%)
Urinary Frequency	1 (2.6%)	0	1 (2.6%)	0	1 (2.7%)	0
White Blood Cell Decreased	0	0	3 (7.7%)	2 (5.1%)	0	0
Anxiety	2 (5.3%)	1 (2.6%)	0	0	0	0
Arthralgia	1 (2.6%)	0	1 (2.6%)	0	0	0
Chest Pain - Cardiac	0	0	1 (2.6%)	0	1 (2.7%)	0
Depression	0	0	2 (5.1%)	0	0	0
General Disorders And Administration Site Conditions - Other, Specify	1 (2.6%)	0	0	0	1 (2.7%)	0
Hypomagnesemia	0	0	0	0	2 (5.4%)	0
Localized Edema	1 (2.6%)	0	0	0	1 (2.7%)	0
Palpitations	1 (2.6%)	0	0	0	1 (2.7%)	0

Table 1A: Priming Phase	ARM A (N = 38)		ARM B (N = 39)		ARM C (N = 37)	
AE Term	Any Grade	Grade 3+	Any Grade	Grade 3+	Any Grade	Grade 3+
Pleural Effusion	0	0	0	0	2 (5.4%)	1 (2.7%)
Pruritus	1 (2.6%)	0	1 (2.6%)	0	0	0
Sinus Tachycardia	2 (5.3%)	0	0	0	0	0
Small Intestinal Obstruction	1 (2.6%)	0	0	0	1 (2.7%)	1 (2.7%)
Acute Kidney Injury	0	0	0	0	1 (2.7%)	1 (2.7%)
Agitation	1 (2.6%)	0	0	0	0	0
Allergic Rhinitis	0	0	0	0	1 (2.7%)	0
Ascites	1 (2.6%)	1 (2.6%)	0	0	0	0
Belching	0	0	0	0	1 (2.7%)	0
Blood And Lymphatic System Disorders - Other, Specify	0	0	1 (2.6%)	1 (2.6%)	0	0
Blurred Vision	0	0	1 (2.6%)	0	0	0
Bone Pain	0	0	1 (2.6%)	0	0	0
Cataract	0	0	0	0	1 (2.7%)	0
Chills	0	0	0	0	1 (2.7%)	0
Concentration Impairment	0	0	0	0	1 (2.7%)	0
Dental Caries	1 (2.6%)	0	0	0	0	0
Dry Skin	1 (2.6%)	0	0	0	0	0
Epistaxis	1 (2.6%)	0	0	0	0	0
Erythema Multiforme	1 (2.6%)	0	0	0	0	0
Fever	1 (2.6%)	0	0	0	0	0
Flushing	1 (2.6%)	0	0	0	0	0
Fracture	1 (2.6%)	0	0	0	0	0
Herpes Simplex Reactivation	0	0	1 (2.6%)	0	0	0
Hypertension	1 (2.6%)	0	0	0	0	0
Hyperthyroidism	0	0	1 (2.6%)	0	0	0
Hypoglycemia	0	0	1 (2.6%)	0	0	0
Hypokalemia	0	0	0	0	1 (2.7%)	0
Hyponatremia	0	0	1 (2.6%)	0	0	0
Infections And Infestations - Other, Specify	1 (2.6%)	0	0	0	0	0
Injury, Poisoning And Procedural Complications - Other, Specify	1 (2.6%)	0	0	0	0	0
Muscle Weakness Lower Limb	0	0	1 (2.6%)	0	0	0
Musculoskeletal And Connective Tissue Disorder - Other, Specify	1 (2.6%)	0	0	0	0	0
Myalgia	0	0	1 (2.6%)	0	0	0
Nail Discoloration	0	0	1 (2.6%)	0	0	0
Neoplasms Benign, Malignant And Unspecified (Incl Cysts And Polyps) - Other, Specify	0	0	0	0	1 (2.7%)	0
Peripheral Motor Neuropathy	1 (2.6%)	0	0	0	0	0
Peripheral Sensory Neuropathy	1 (2.6%)	0	0	0	0	0
Pleuritic Pain	0	0	0	0	1 (2.7%)	1 (2.7%)

Table 1A: Priming Phase	ARM A (N = 38)		ARM B (N = 39)		ARM C (N = 37)	
**AE Term**	**Any Grade**	**Grade 3+**	**Any Grade**	**Grade 3+**	**Any Grade**	**Grade 3+**
Psychiatric Disorders - Other, Specify	0	0	1 (2.6%)	0	0	0
Rash Acneiform	0	0	1 (2.6%)	0	0	0
Renal And Urinary Disorders - Other, Specify	1 (2.6%)	0	0	0	0	0
Respiratory, Thoracic And Mediastinal Disorders - Other, Specify	0	0	1 (2.6%)	0	0	0
Rhinorrhea	0	0	0	0	1 (2.7%)	0
Shingles	0	0	1 (2.6%)	0	0	0
Skin Hyperpigmentation	0	0	1 (2.6%)	0	0	0
Skin Infection	0	0	1 (2.6%)	0	0	0
Stroke	1 (2.6%)	0	0	0	0	0
Thrush	1 (2.6%)	0	0	0	0	0
Upper Respiratory Infection	0	0	1 (2.6%)	0	0	0
Urinary Incontinence	0	0	0	0	1 (2.7%)	0
Urinary Tract Obstruction	0	0	1 (2.6%)	1 (2.6%)	0	0
Urinary Tract Pain	0	0	1 (2.6%)	0	0	0
Vision Decreased	0	0	0	0	1 (2.7%)	1 (2.7%)

Data are number and % of participants experiencing a given AE (worst grade used).

Denominator is number of participants that received treatment during priming phase.

Table 1B: Consolidation Phase	ARM A (N = 37)		ARM B (N = 35)		ARM C (N = 30)	
**AE Term**	**Any Grade**	**Grade 3+**	**Any Grade**	**Grade 3+**	**Any Grade**	**Grade 3+**
Fatigue	23 (62.2%)	1 (2.7%)	24 (68.6%)	1 (2.9%)	20 (66.7%)	2 (6.7%)
Nausea	19 (51.4%)	0	23 (65.7%)	0	19 (63.3%)	0
Anemia	11 (29.7%)	4 (10.8%)	16 (45.7%)	5 (14.3%)	9 (30.0%)	2 (6.7%)
Constipation	8 (21.6%)	0	12 (34.3%)	0	7 (23.3%)	0
Abdominal Pain	8 (21.6%)	1 (2.7%)	6 (17.1%)	0	7 (23.3%)	2 (6.7%)
Dysgeusia	8 (21.6%)	0	6 (17.1%)	0	7 (23.3%)	0
Anorexia	6 (16.2%)	1 (2.7%)	6 (17.1%)	0	6 (20.0%)	0
Diarrhea	6 (16.2%)	0	6 (17.1%)	0	6 (20.0%)	1 (3.3%)
Rash Maculo-Papular	9 (24.3%)	0	5 (14.3%)	0	3 (10.0%)	0
Neutrophil Count Decreased	2 (5.4%)	0	8 (22.9%)	6 (17.1%)	3 (10.0%)	0
Pain	3 (8.1%)	0	7 (20.0%)	0	2 (6.7%)	1 (3.3%)
Vomiting	3 (8.1%)	0	5 (14.3%)	0	4 (13.3%)	0
Arthralgia	5 (13.5%)	0	5 (14.3%)	1 (2.9%)	1 (3.3%)	0
Cough	2 (5.4%)	0	5 (14.3%)	1 (2.9%)	4 (13.3%)	0
Gastroesophageal Reflux Disease	5 (13.5%)	0	3 (8.6%)	0	3 (10.0%)	0
Creatinine Increased	4 (10.8%)	0	4 (11.4%)	0	2 (6.7%)	0
Hypothyroidism	5 (13.5%)	0	4 (11.4%)	0	1 (3.3%)	0
Urinary Tract Infection	3 (8.1%)	0	4 (11.4%)	0	3 (10.0%)	0

Table 1B: Consolidation Phase	ARM A (N = 37)		ARM B (N = 35)		ARM C (N = 30)	
**AE Term**	**Any Grade**	**Grade 3+**	**Any Grade**	**Grade 3+**	**Any Grade**	**Grade 3+**
Back Pain	2 (5.4%)	0	3 (8.6%)	0	4 (13.3%)	1 (3.3%)
Dyspnea	3 (8.1%)	0	3 (8.6%)	0	3 (10.0%)	1 (3.3%)
Insomnia	6 (16.2%)	0	1 (2.9%)	0	2 (6.7%)	0
Alopecia	1 (2.7%)	0	6 (17.1%)	0	1 (3.3%)	0
Edema Limbs	3 (8.1%)	0	3 (8.6%)	0	2 (6.7%)	0
Mucositis Oral	4 (10.8%)	1 (2.7%)	3 (8.6%)	0	1 (3.3%)	0
Pruritus	2 (5.4%)	0	4 (11.4%)	0	2 (6.7%)	0
Infections And Infestations - Other, Specify	2 (5.4%)	0	2 (5.7%)	0	3 (10.0%)	0
Peripheral Sensory Neuropathy	4 (10.8%)	0	0	0	3 (10.0%)	0
Headache	3 (8.1%)	0	1 (2.9%)	0	2 (6.7%)	0
Hyperthyroidism	4 (10.8%)	0	2 (5.7%)	0	0	0
Muscle Cramp	3 (8.1%)	0	3 (8.6%)	0	0	0
Palpitations	4 (10.8%)	0	1 (2.9%)	0	1 (3.3%)	0
Bloating	1 (2.7%)	0	2 (5.7%)	0	2 (6.7%)	0
Dyspepsia	1 (2.7%)	0	3 (8.6%)	0	1 (3.3%)	0
Hot Flashes	1 (2.7%)	0	2 (5.7%)	0	2 (6.7%)	0
Lethargy	3 (8.1%)	0	1 (2.9%)	0	1 (3.3%)	0
Pain In Extremity	2 (5.4%)	0	2 (5.7%)	1 (2.9%)	1 (3.3%)	0
Platelet Count Decreased	1 (2.7%)	1 (2.7%)	3 (8.6%)	0	1 (3.3%)	0
Anxiety	3 (8.1%)	1 (2.7%)	0	0	1 (3.3%)	0
Aspartate Aminotransferase Increased	1 (2.7%)	0	2 (5.7%)	1 (2.9%)	1 (3.3%)	0
Dizziness	2 (5.4%)	1 (2.7%)	1 (2.9%)	0	1 (3.3%)	0
Dysuria	1 (2.7%)	0	2 (5.7%)	0	1 (3.3%)	0
Lymphocyte Count Decreased	1 (2.7%)	0	3 (8.6%)	2 (5.7%)	0	0
Myalgia	2 (5.4%)	0	2 (5.7%)	0	0	0
Obstruction Gastric	2 (5.4%)	2 (5.4%)	0	0	2 (6.7%)	2 (6.7%)
Upper Respiratory Infection	1 (2.7%)	0	2 (5.7%)	1 (2.9%)	1 (3.3%)	0
White Blood Cell Decreased	1 (2.7%)	0	3 (8.6%)	3 (8.6%)	0	0
Alanine Aminotransferase Increased	1 (2.7%)	0	1 (2.9%)	1 (2.9%)	1 (3.3%)	0
Arthritis	2 (5.4%)	0	1 (2.9%)	0	0	0
Bone Pain	1 (2.7%)	0	2 (5.7%)	0	0	0
Chest Wall Pain	0	0	2 (5.7%)	0	1 (3.3%)	0
Dry Mouth	2 (5.4%)	0	1 (2.9%)	0	0	0
General Disorders And Administration Site Conditions - Other, Specify	2 (5.4%)	0	0	0	1 (3.3%)	0
Hypomagnesemia	1 (2.7%)	0	1 (2.9%)	0	1 (3.3%)	0
Neoplasms Benign, Malignant And Unspecified (Incl Cysts And Polyps) - Other, Specify	0	0	2 (5.7%)	1 (2.9%)	1 (3.3%)	0
Skin And Subcutaneous Tissue Disorders - Other, Specify	0	0	2 (5.7%)	0	1 (3.3%)	0
Vertigo	0	0	3 (8.6%)	2 (5.7%)	0	0

Table 1B: Consolidation Phase	ARM A (N = 37)		ARM B (N = 35)		ARM C (N = 30)	
AE Term	Any Grade	Grade 3+	Any Grade	Grade 3+	Any Grade	Grade 3+
Weight Loss	0	0	1 (2.9%)	0	2 (6.7%)	0
Acute Kidney Injury	1 (2.7%)	0	0	0	1 (3.3%)	1 (3.3%)
Alkaline Phosphatase Increased	1 (2.7%)	0	1 (2.9%)	0	0	0
Allergic Reaction	1 (2.7%)	0	0	0	1 (3.3%)	0
Chest Pain - Cardiac	0	0	1 (2.9%)	0	1 (3.3%)	0
Depression	0	0	2 (5.7%)	0	0	0
Dry Skin	2 (5.4%)	0	0	0	0	0
Dysphagia	1 (2.7%)	0	0	0	1 (3.3%)	0
Facial Pain	1 (2.7%)	0	1 (2.9%)	0	0	0
Fever	2 (5.4%)	0	0	0	0	0
Flu Like Symptoms	1 (2.7%)	0	1 (2.9%)	0	0	0
Gastrointestinal Pain	1 (2.7%)	0	0	0	1 (3.3%)	0
Hepatic Pain	1 (2.7%)	0	1 (2.9%)	0	0	0
Hoarseness	2 (5.4%)	0	0	0	0	0
Hyperglycemia	1 (2.7%)	0	1 (2.9%)	0	0	0
Hypokalemia	0	0	1 (2.9%)	0	1 (3.3%)	0
Hyponatremia	1 (2.7%)	0	1 (2.9%)	0	0	0
Immune System Disorders - Other, Specify	0	0	2 (5.7%)	1 (2.9%)	0	0
Kidney Infection	2 (5.4%)	2 (5.4%)	0	0	0	0
Lipase Increased	1 (2.7%)	0	1 (2.9%)	1 (2.9%)	0	0
Localized Edema	1 (2.7%)	0	0	0	1 (3.3%)	0
Lung Infection	0	0	1 (2.9%)	0	1 (3.3%)	0
Middle Ear Inflammation	1 (2.7%)	0	1 (2.9%)	0	0	0
Mucosal Infection	1 (2.7%)	0	0	0	1 (3.3%)	0
Musculoskeletal And Connective Tissue Disorder - Other, Specify	1 (2.7%)	0	1 (2.9%)	0	0	0
Pleural Effusion	1 (2.7%)	1 (2.7%)	1 (2.9%)	1 (2.9%)	0	0
Rash Acneiform	1 (2.7%)	0	1 (2.9%)	0	0	0
Sinus Tachycardia	2 (5.4%)	0	0	0	0	0
Sinusitis	1 (2.7%)	0	1 (2.9%)	0	0	0
Skin Infection	1 (2.7%)	0	0	0	1 (3.3%)	0
Thromboembolic Event	1 (2.7%)	1 (2.7%)	0	0	1 (3.3%)	0
Thyroid Stimulating Hormone Increased	1 (2.7%)	0	0	0	1 (3.3%)	0
Tooth Infection	1 (2.7%)	0	1 (2.9%)	0	0	0
Urinary Frequency	1 (2.7%)	0	0	0	1 (3.3%)	0
Urinary Tract Pain	1 (2.7%)	0	1 (2.9%)	0	0	0
Vaginal Dryness	0	0	1 (2.9%)	0	1 (3.3%)	0
Abdominal Distension	0	0	0	0	1 (3.3%)	0
Agitation	1 (2.7%)	0	0	0	0	0
Allergic Rhinitis	1 (2.7%)	0	0	0	0	0
Anal Pain	0	0	1 (2.9%)	0	0	0
Ankle Fracture	0	0	0	0	1 (3.3%)	1 (3.3%)

Table 1B: Consolidation Phase	ARM A (N = 37)		ARM B (N = 35)		ARM C (N = 30)	
**AE Term**	**Any Grade**	**Grade 3+**	**Any Grade**	**Grade 3+**	**Any Grade**	**Grade 3+**
Ataxia	0	0	0	0	1 (3.3%)	0
Belching	0	0	0	0	1 (3.3%)	0
Bladder Infection	1 (2.7%)	1 (2.7%)	0	0	0	0
Blood And Lymphatic System Disorders - Other, Specify	0	0	0	0	1 (3.3%)	0
Blood Prolactin Abnormal	0	0	0	0	1 (3.3%)	0
Bronchospasm	0	0	1 (2.9%)	0	0	0
Bruising	1 (2.7%)	0	0	0	0	0
Bullous Dermatitis	0	0	1 (2.9%)	0	0	0
Burn	0	0	1 (2.9%)	0	0	0
Cholesterol High	0	0	1 (2.9%)	0	0	0
Colitis	0	0	1 (2.9%)	0	0	0
Concentration Impairment	0	0	0	0	1 (3.3%)	0
Dry Eye	1 (2.7%)	0	0	0	0	0
Duodenal Obstruction	1 (2.7%)	1 (2.7%)	0	0	0	0
Erythema Multiforme	0	0	0	0	1 (3.3%)	0
Eye Disorders - Other, Specify	1 (2.7%)	0	0	0	0	0
Eye Infection	1 (2.7%)	0	0	0	0	0
Fall	1 (2.7%)	0	0	0	0	0
Flank Pain	0	0	1 (2.9%)	0	0	0
Gastritis	0	0	1 (2.9%)	0	0	0
Gastrointestinal Disorders - Other, Specify	1 (2.7%)	1 (2.7%)	0	0	0	0
Ggt Increased	1 (2.7%)	0	0	0	0	0
Hearing Impaired	1 (2.7%)	0	0	0	0	0
Hemorrhoids	0	0	0	0	1 (3.3%)	0
Hypercalcemia	0	0	1 (2.9%)	0	0	0
Hyperkalemia	0	0	1 (2.9%)	0	0	0
Hyperkeratosis	0	0	0	0	1 (3.3%)	0
Hyperphosphatemia	1 (2.7%)	0	0	0	0	0
Hypertension	1 (2.7%)	0	0	0	0	0
Hypoglycemia	0	0	0	0	1 (3.3%)	0
Hypoparathyroidism	0	0	1 (2.9%)	0	0	0
Hypophosphatemia	1 (2.7%)	0	0	0	0	0
Hypopituitarism	1 (2.7%)	0	0	0	0	0
Hypotension	0	0	1 (2.9%)	0	0	0
Injection Site Reaction	0	0	0	0	1 (3.3%)	0
Joint Effusion	1 (2.7%)	0	0	0	0	0
Lymphocyte Count Increased	0	0	1 (2.9%)	0	0	0
Malaise	0	0	0	0	1 (3.3%)	1 (3.3%)
Metabolism And Nutrition Disorders - Other, Specify	1 (2.7%)	0	0	0	0	0
Myelodysplastic Syndrome	0	0	1 (2.9%)	1 (2.9%)	0	0

Table 1B: Consolidation Phase	ARM A (N = 37)		ARM B (N = 35)		ARM C (N = 30)	
AE Term	Any Grade	Grade 3+	Any Grade	Grade 3+	Any Grade	Grade 3+
Nail Changes	1 (2.7%)	0	0	0	0	0
Nail Discoloration	0	0	1 (2.9%)	0	0	0
Nervous System Disorders - Other, Specify	0	0	0	0	1 (3.3%)	0
Oral Dysesthesia	1 (2.7%)	0	0	0	0	0
Osteoporosis	0	0	0	0	1 (3.3%)	0
Papulopustular Rash	0	0	1 (2.9%)	0	0	0
Paresthesia	0	0	1 (2.9%)	0	0	0
Periodontal Disease	0	0	1 (2.9%)	0	0	0
Peripheral Motor Neuropathy	1 (2.7%)	0	0	0	0	0
Psychiatric Disorders - Other, Specify	0	0	1 (2.9%)	0	0	0
Renal And Urinary Disorders - Other, Specify	1 (2.7%)	0	0	0	0	0
Renal Calculi	1 (2.7%)	1 (2.7%)	0	0	0	0
Rhinitis Infective	1 (2.7%)	0	0	0	0	0
Rhinorrhea	1 (2.7%)	0	0	0	0	0
Shingles	0	0	1 (2.9%)	0	0	0
Skin Hyperpigmentation	0	0	1 (2.9%)	0	0	0
Soft Tissue Infection	0	0	0	0	1 (3.3%)	0
Stroke	1 (2.7%)	0	0	0	0	0
Superficial Thrombophlebitis	0	0	1 (2.9%)	0	0	0
Tremor	0	0	1 (2.9%)	0	0	0
Urinary Incontinence	0	0	0	0	1 (3.3%)	0
Urinary Tract Obstruction	0	0	1 (2.9%)	1 (2.9%)	0	0
Urticaria	0	0	0	0	1 (3.3%)	0
Vaginal Hemorrhage	1 (2.7%)	0	0	0	0	0
Wound Infection	1 (2.7%)	0	0	0	0	0

Data are number and % of participants experiencing a given AE (worst grade used).

Denominator is number of participants that received treatment during consolidation phase.

Supplementary Table 2
Immune related adverse events by treatment arm
AE, adverse event;

	ARM A (N = 37)		ARM B (N = 35)		ARM C (N = 30)	
**AE Term**	**Any Grade**	**Grade 3+**	**Any Grade**	**Grade 3+**	**Any Grade**	**Grade 3+**
Hypothyroidism	5 (13.5%)	0	3 (8.6%)	0	0	0
Hyperthyroidism	3 (8.1%)	0	2 (5.7%)	0	0	0
Pruritus	0	0	2 (5.7%)	0	0	0
Rash Maculo-Papular	0	0	2 (5.7%)	0	0	0
Acute Kidney Injury	1 (2.7%)	0	0	0	0	0
Alanine Aminotransferase Increased	0	0	1 (2.9%)	1 (2.9%)	0	0
Aspartate Aminotransferase Increased	0	0	1 (2.9%)	1 (2.9%)	0	0
Dry Mouth	1 (2.7%)	0	0	0	0	0
Hypopituitarism	1 (2.7%)	0	0	0	0	0
Joint Effusion	1 (2.7%)	0	0	0	0	0
Mucositis Oral	1 (2.7%)	0	0	0	0	0

Supplementary note 2

SOLACE2 trial protocol

NHMRC Clinical Trials Centre

SOLACE2: A Phase II randomised trial comparing immune priming by low dose oral cyclophosphamide plus olaparib versus priming by olaparib alone, prior to combination therapy with olaparib plus durvalumab, versus single agent olaparib alone, in asymptomatic platinum-sensitive recurrent ovarian, fallopian tube or primary peritoneal cancers with homologous recombination repair defects

NHMRC CTC protocol number: CTC 0178 / ANZGOG 1723/2018

Protocol Version 5.0 06 May 2021

Australian Sponsor: **The University of Sydney**
NSW 2006 Australia

This study is a collaboration between the Australia New Zealand Gynaecological Oncology Group and the NHMRC Clinical Trials Centre, University of Sydney.

ANZCTR Number: ANZCTR12618000686202

Study Chair: Professor Clare Scott
Study Co-Chairs: A/Prof Chee Khoon Lee
Professor Michael Friedlander
Translational Science Chair: Professor Magdalena Plebanski

Coordinating Centre: NHMRC Clinical Trials Centre
92-94 Parramatta Road
Camperdown NSW 2050
Telephone: 61-2-9562-5000
Fax: 61-2-9565-1863
Email: solace2@ctc.usyd.edu.au

CONFIDENTIAL

Senior Statistician: Rachel O'Connell

CTC Clinical Lead: Martin Stockler

Project Manager: Katrina Diamante

Protocol Development Working Party

The following individuals also contributed to the design and development of this protocol:

Name	Position	Organisation
Dr Monica Tang	ANZGOG Clinical Research Fellow	CTC
Dr Peey Sei Kok	ANZGOG Clinical Research Fellow	CTC
Dr Elisa Carolus	ANZGOG Clinical Research Fellow	CTC
Dr Yeh Chen Lee	ANZGOG Clinical Research Fellow	CTC
Candace Carter	ANZGOG Associate Oncology Program Manager	CTC
Tracey Meares	Clinical Research Project Manager	ANZGOG

Abbreviations

AE	Adverse Event
ALP	Alkaline phosphatase
ALT	Alanine aminotransferase
APTT	Activated partial thromboplastin time
AST	Aspartate aminotransferase
BD	Bi-daily
BP	Blood Pressure
CBR	Clinical benefit rate
CT	Computed tomography (scan)
CRF	Case report form
CTC	NHMRC Clinical Trials Centre, University of Sydney
CTCAE	Common Terminology Criteria for Adverse Events
CR	Complete Response
CTC	NHMRC Clinical Trials Centre
DNA	Deoxyribonucleic acid
DRG	Diagnosis Related Groups
EBRT	External beam radiation therapy
EGFR	Epidermal Growth Factor receptor
EORTC	European Organisation for Research and Treatment of Cancer
EQ-5D-5L	Euroqol 5 item preference-based measure of health (5L)
FDA	Food and Drug Administration
FFPE	Formalin-Fixed Paraffin-Embedded
GIST	Gastrointestinal Stromal Tumour
GP	General Practitioner
GSA	Group Specific Appendix
HREC	Human Research Ethics Committee
HIV	Human immunodeficiency virus
HR	Hazard Ratio
HRQL	Health-Related Quality of Life
IC_{50}	50% maximal inhibitory concentration
ICER	Incremental cost effectiveness ratio
IDSMC	Independent Data, Safety Monitoring Committee
INR	International Normalised Ratio
ISC	International Steering Committee
MBS	Medicare Benefits Scheme (Australia)
NCIC CTG	Canadian NCIC Clinical Trials Group
NYHA	New York Heart Association
ORR	Objective response rate
OS	Overall survival
OTRR	Objective tumour response rate
PBS	Pharmaceutical Benefits Scheme (Australia)
PET	Positron emission tomography
PDGFRA	Platelet-derived growth factor receptor alpha
PFS	Progression Free Survival
PD	Progressive Disease
PR	Partial Response
PR-25	EORTC Quality of Life Questionnaire for Prostate Cancer (25 items)

PT	Prothrombin Time
QLQ-C30	EORTC Core Quality of Life Questionnaire (30 items)
QOL	Quality of life
RECIST	Response Evaluation Criteria in Solid Tumours
SAE	Serious adverse event
SD	Stable Disease
SUSAR	Suspected Unexpected Serious Adverse Reaction
SUV	Standardised uptake values
TGA	Therapeutic Goods Administration
TFT	Thyroid Function Tests
TKI	Tyrosine Kinase inhibitor
ULN	Upper limit of normal
WBBS	Whole Body Bone Scan

Table of Contents

Contents

SYNOPSIS AND SCHEMA 8
1 BACKGROUND 11
1.1 SOLACE: A phase 1 study of olaparib (O) + LDCy (C) in HGSOC and TNBC/BRCA1/2 BC 12
1.2 Role of Immunological Priming: 12
1.3 Platinum Sensitivity as a Surrogate for DNA Repair Defective Ovarian Cancer 13
1.4 Durvalumab 14
1.4.1 Non-clinical and Clinical Experience 14
1.4.2 Safety 14
1.5 Hypothesis 15
1.6 Patient Reported Outcomes 15
1.6.1 SOLACE2 PRO Endpoints 15
2 AIM AND OBJECTIVES 15
3 DESIGN 17
4 STUDY POPULATION 17
4.1 Target Population 17
4.2 Inclusion Criteria 17
4.3 Exclusion Criteria 18
4.4 Study Enrolment 20
4.4.1 Screening 20
4.4.2 Registration and Randomisation 20
5 TREATMENT PLAN 20
5.1 Treatments 20
5.1.1 Study Treatment 20
5.1.2 Required Background Treatment 21
5.1.3 Olaparib 21
5.1.4 Durvalumab Administration 22
5.1.5 Monitoring of Durvalumab Administration 22
5.2 Dose Modifications 22
5.2.1 Dose Levels 23
5.2.2 Dose Modifications for Adverse Events 23
5.3 Post-study treatment 27
5.4 Concomitant Medications 27
5.4.1 Recommended 27
5.4.2 Prohibited 27
5.4.3 Use with Caution 27
5.4.4 Concomitant Medication Reporting 28
5.5 Restrictions During the Study 28
5.5.1 Contraception 28
5.6 Treatment Compliance 29
5.7 Study Treatment Discontinuation 29
6 ASSESSMENT PLAN 30
6.1 Schedule of Assessments 30
6.2 Details of Assessments 32
6.2.1 Screening phase 32
6.2.2 Clinical Assessment 32
6.2.3 Imaging 32
6.2.4 Quality of Life 33
6.2.5 Blood Collection 33
6.2.6 Tissue Collection 33
6.2.7 Ascites/ Pleural Fluid Collection 33
6.3 Follow-up After Treatment 33

7 OUTCOMES, ENDPOINTS AND OTHER MEASUREMENTS 34
7.1 Progression Free Survival 34
7.2 Objective Response Rate 34
7.3 Frequency and Severity of Adverse Events 34
7.4 Health Related Quality of Life 34
7.5 Time to Starting First Subsequent Therapy or Death 35
7.6 Tertiary/correlative Measures 35
8 SAFETY REPORTING 35
8.1 Definitions 35
8.2 Adverse Events of Special Interest 37
8.2.1 AESIs for Durvalumab 37
8.2.2 AESIs for Olaparib 38
8.3 Recording Adverse Events 38
8.4 Pregnancy 38
8.5 Overdose 38
8.5.1 Olaparib 38
8.5.2 Cyclophosphamide 39
8.6 Reporting of Serious Adverse Events (including SUSARs) 39
9 CENTRAL REVIEW 39
9.1 Central Tissue Collection 39
9.2 Central Blood Collection 39
9.3 Central Ascites/ Pleural Fluid Collection 40
10 CENTRAL STORAGE OF BIOSPECIMENS 40
11 STUDY TREATMENT SUPPLY AND ACCOUNTABILITY 40
11.1 Study Treatment and Supply - Durvalumab 40
11.2 Study Treatment and Supply – Olaparib 41
11.3 Study Treatment and Supply - Cyclophosphamide (LDCy) 41
11.4 Drug Accountability 41
11.5 Background treatment 41
12 STATISTICAL CONSIDERATIONS 41
12.1 Sample Size 41
12.2 Event Monitoring 42
12.3 Statistical Analysis 42
12.3.1 Primary Endpoint 42
12.3.2 Secondary Endpoints 42
13 STUDY ORGANISATION and COMMITTEES 42
13.1 Study coordination 42
13.2 Trial Management Committee 42
13.3 Independent Safety and Data Monitoring Committee 43
14 ADMINISTRATIVE ASPECTS 43
14.1 Ethics and regulatory compliance 43
14.2 Confidentiality 43
14.3 Protocol deviations and amendments 43
14.4 Data Handling and Record Keeping 44
14.5 Study Monitoring 44
14.6 Audit and Inspection 44
14.7 Clinical Study Report 44
14.8 Publication Policy 44
15 PROTOCOL AMENDMENTS 45
16 REFERENCES 48
17 APPENDICES 51
17.1 Appendix 1 ECOG Performance Status Scales 51
17.2 Appendix 2 Cockroft-Gault formula 52
17.3 Appendix 3 Management of Infusion-related Reactions Associated with Durvalumab .53
17.4.1 Pneumonitis/Interstitial Lung Disease Management Algorithm 55

17.4.2 Diarrhoea/Colitis Management Algorithm 56
17.4.3 Hepatitis/ Elevated LFTs Management Algorithm 58
17.4.4 Nephritis or Renal Dysfunction (elevated serum creatinine) Management Algorithm 60
17.4.5 Rash or Dermatitis (including Pemphigoid) Management Algorithm 61
17.4.6 Endocrinopathy Management Algorithm 62
17.4.6.1 Amylase/Lipase Increased 64
17.4.6.2 Acute Pancreatitis 64
17.4.7 Immune-mediated Neurotoxicity (to include, but not be limited to non-infectious meningitis, non-infectious encephalitis and automatic neuropathy, excluding Myasthenia Gravis and Guillain-Barre) Management Algorithm 65
17.4.8 Immune-mediated Peripheral Neuromotor Syndromes, such as Guillain-Barre and Myasthenia Gravis, Management Algorithm 66
17.4.9 Myocarditis Management Algorithm 68
17.4.10 Myositis/Polymyositis Management Algorithm 69
17.4.11 Other Immune-Mediated Reactions 71
17.5.1 CYP3A4 INDUCERS (PROHIBITED) 72
17.5.2 CYP3A4 INHIBITORS 72
17.5.3 CYP3A4 Substrates (Allowed – take note of possible interactions) 73
17.6 Appendix 6 EORTC QLQ-C30 Questionnaire 75
17.8 Appendix 8 MOST Questionnaire 79
17.9.1 Disease and Lesion Definitions 81
17.9.2 Response Definitions 82
17.11 Appendix 11 iRECIST Criteria 85
17.11.1 Confirming Progression 85
17.11.2 New Lesions (NL) 85
17.11.3 Time-point (TP) iResponse 86
17.11.4 iRECIST Best Overall Response (iBOR) 87
17.11.5 Response and Stable Disease Duration (RECIST 1.1 and iRECIST) 87

SYNOPSIS AND SCHEMA

**Background**	Up to 50% of high grade ovarian cancer (OC) may have impaired DNA repair by homologous recombination (HR). HR defective OC is associated with response to the PARP inhibitor (PARPi) olaparib. Low dose cyclophosphamide (LDCy) promotes anti-tumour immunity through selective depletion of regulatory T cells (Tregs), which suggests potential for enhancing the efficacy of checkpoint inhibitor therapies with prior sequencing of LDCy. The Phase 1 SOLACE study demonstrated that the combination of olaparib and LDCy is well-tolerated and active, particularly in HR defective high grade serous ovarian cancer (HGSOC). Hence, immunological "priming" with LDCy in combination with olaparib could improve subsequent response to checkpoint inhibitor therapy in women with HGSOC at first relapse.
**Aim**	To determine the efficacy and tolerability of three months of olaparib + low dose oral cyclophosphamide followed by olaparib + durvalumab, versus three months of olaparib monotherapy followed by olaparib + durvalumab, versus olaparib monotherapy alone, in asymptomatic women at first CA125 progression with platinum sensitive recurrent ovarian, fallopian tube or primary peritoneal cancer.
**Primary objective**	To determine in each treatment arm:<br>1) 36 week progression-free survival rate (36wPFS, death or PD by RECIST 1.1 at 36 weeks)
**Secondary objectives**	2) Objective response (OR = CR or PR) rate (ORR) by RECIST 1.1 or GCIG CA125 criteria<br>3) Progression free survival (PFS) by RECIST 1.1 or GCIG CA125 criteria<br>4) Objective tumour response (OTR = CR or PR) rate (OTRR) and PFS by iRECIST<br>5) Frequency and severity of adverse events (CTCAE v5.0)<br>6) Aspects of health related quality of life (EORTC QLQ-C30, OV28 and MOST)<br>7) Time to starting first subsequent therapy (TFST) or death<br>8) Time to development of symptoms associated with progression, specifically abdominal/gastrointestinal symptoms
**Tertiary and correlative objectives**	9) To determine whether an immune biomarker can be used to reliably and practically identify patients, diagnostically or prognostically, who will derive maximal beneficial effects from PARPi and/or LDCy combination and /or immune therapy.<br>10) To test whether an immune signature of T cell function, can be used to predict tumour response for the PARPi and/or LDCy combination and /or immune therapy.<br>11) A prognostic (pre-treatment) and diagnostic (post-treatment) Cyclophosphamide-UPregulation (CUP) test may practically identify responders to PARPi and/or LDCy combination and /or immune therapy.

12) To test whether genomics analyses of the baseline tumour or post-progression biopsy and/or additional immunological analyses not restricted to T cell function can be used to predict tumour response for the PARPi and/or LDCy combination and/or immune therapy, and to predict the consequences of combined or sequential application of these therapies.

13) To test whether baseline tumour PDL1 expression on immunohistochemistry is predictive of benefit with durvalumab therapy

14) To test whether change in ctDNA from baseline could be a valid surrogate marker for treatment benefit

**Study design**	Open-label, randomised, 3-arm, multicentre phase II clinical trial. Participants will be allocated to 3 treatment groups in a ratio of 1:1:1
**Population**	Women with platinum sensitive high-grade serous carcinoma of the ovary, fallopian tube or primary peritoneum, at first asymptomatic CA125 progression
**Study treatments**	Participants will be randomised to:<br>A) Olaparib tablets 300mg orally BD for 12 weeks, followed in the absence of progression according to RECIST 1.1 by olaparib 300 mg BD + durvalumab 1,500mg IV every 4 weeks for 36 cycles, followed by olaparib alone until progression;<br>B) Olaparib 300mg BD + oral cyclophosphamide 50mg daily on days 1-5 consecutively each week (1000mg per 4 weeks), for 3 x four- weekly cycles over 12 weeks, followed in the absence of progression according to RECIST 1.1 by olaparib + durvalumab (dosing schedule as above) for 36 cycles, followed by olaparib alone until progression;<br>C) Olaparib 300mg BD until progression.
**Assessments**	Clinical assessments and blood tests every 4 weeks during treatment. From randomisation, CT chest, abdomen and pelvis at 8 weeks, 24 weeks and then every 12 weeks until progression. Blood for tertiary correlative studies at study entry, pre-treatment, every 2 weeks during cycles 1-3 (priming phase), then every 4 weeks during cycles 4-15 (early consolidation phase), every 16 weeks during cycles 16-39 (late consolidation phase) and at progression.
**Statistical considerations**	Using Fleming's single stage design, with 95% confidence and 80% power, 38 participants per arm will be required to exclude an uninteresting PFS rate at 36 weeks (9-months) of 47% in favour of a more clinically meaningful PFS rate of 67%. Therefore, in each of the combination arms (arms A and B), 38 participants will be recruited. A control arm (arm C) also with 38 participants is important to help determine that the assumption of olaparib monotherapy with a 36 week PFS rate of ~ 47% is true. Additionally as there are 3 groups a total of 114 participants will also allow a minimum of 15% difference from the best performing regimen to be detected with a probability >0.80.<br>Therefore, the overall sample size is 38+38+38 = 114 participants.

NHMRC Clinical Trials Centre

Study Schema

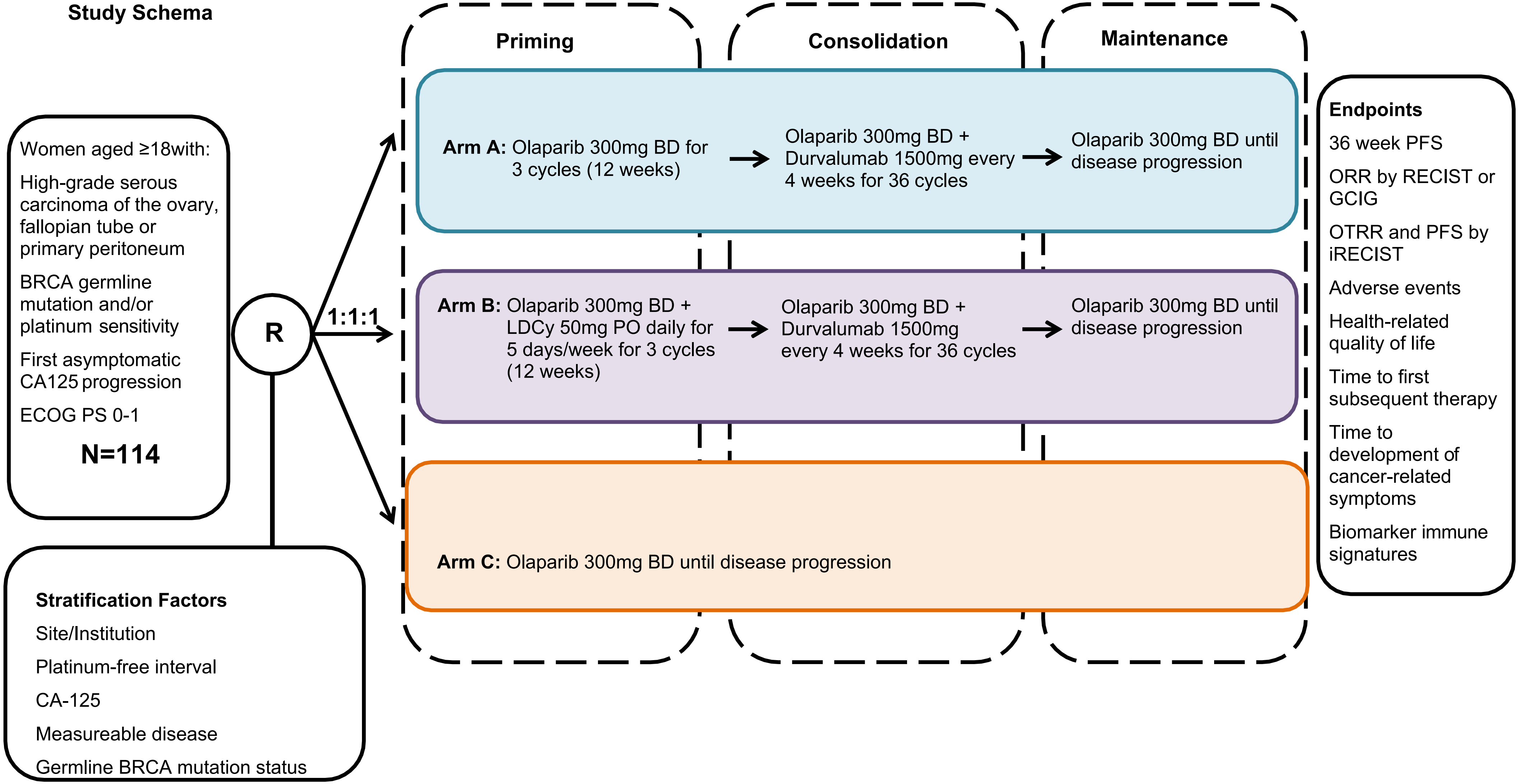

1 BACKGROUND

High-grade serous ovarian cancer (OC) is a major cause of cancer death in women. Up to 50% of OC may have impaired DNA repair by homologous recombination (HR), including 20% by germline or somatic mutation of *BRCA1* or *BRCA2.* HR defective OC is associated with improved overall survival following standard chemotherapy(1, 2) and response to the PARP inhibitor, olaparib.(3-6) The potential of targeted therapies to improve outcomes for women with high-grade serous ovarian cancer is on the horizon for the first time. PARP inhibitor therapy has been approved by the TGA and the PBAC for *BRCA1/2*-mutated platinum-sensitive HGSOC in the maintenance setting. Beyond *BRCA1/2* mutated HGSOC, screening tests for DNA repair defective therapy have not yet proven to be more accurate than platinum sensitivity in selection for PARPi responders. Immunotherapy, as for many solid tumour types, is of great interest but single agent therapy with inhibitors of PD1 or PD-L1 have relatively limited response rates in epithelial OC of only 15% with disease control rates of 22% - 45%.(7, 8) The patient's immune status, as indicated by the presence of tumour infiltrating CD8 T cells (and a high CD8 T cell/Treg ratio) is also associated with substantially better survival outcomes.(9, 10) Conversely, the presence of suppressor cells such as regulatory T cells (Treg) is strongly correlated with disease recurrence.(11-13) PARP inhibitor therapy causes DNA damage, potentially causing greater mutational burden, expanding neoantigen expression, leading to a greater immune recognition of the tumour.(14, 15) This suggests that PARP inhibitor therapy may be complementary to checkpoint inhibitor therapy.

Insights from our pre-clinical work (unpublished) and from the SOLACE study (16) have led us to design the SOLACE2 study, to address the issue of whether a cheap and well-tolerated combination therapy (oral olaparib and low dose oral cyclophosphamide (LDCy)) could prime the immune response, improving the proportion of patients whose disease is subsequently able to respond to olaparib/durvalumab therapy, perhaps converting more ovarian cancer patients into "super-responders", as already observed for a subset of *BRCA1/2* mutant cases. As immune responses are more likely, the earlier in the disease trajectory immunotherapy treatment is received, we propose to explore this at the first indication of relapse of HGSOC, in asymptomatic patients with CA125 progression, with or without small volume recurrent disease on CT scan and in whom chemotherapy or surgery is not indicated.

LDCy promotes anti-tumour immunity(17), primarily but not exclusively by selective depletion of Treg, as Treg and T effector cell metabolic adaptations make T cells differentially responsive to LDCy. This suggests the potential to **enhance the efficacy of checkpoint inhibitor therapies with prior sequencing of LDCy.** LDCy is currently used as palliative therapy in patients who have undergone multiple lines of other treatments, often having developed resistance to both taxanes and platinum. In this late setting, LDCy results in clinical benefit for 25-44% of patients OC(18).

In a pilot study in 10 women with recurrent OC treated with LDCy, we observed that those who experienced clinical benefit from LDCy (40%), were also observed to have treatment-induced increases in a specific subset of CD4+ T cells and CD8+ effector T cells, but not in Treg in their peripheral blood *in vivo*. These increases correlated with induction *in vitro* of this specific subset by the cyclophosphamide analogue, mafosfamide, in cultured T cells from the same patients**, even in pre-treatment samples**. This small pilot study was conducted to determine whether the specific biomarker appeared to associate with better outcome following treatment with LDCy. SOLACE2 translational studies have been designed to determine which of a range of biomarkers will be most relevant for predicting response to treatment.

Our findings offer the very real possibility of **prospective identification using a novel *in vitro* biomarker blood test, to identify patients who will gain the greatest immunotherapeutic benefit with the addition of LDCy to olaparib.** There are few clinical trials of LDCy in women with recurrent OC and there are no data on responses rates /clinical benefit with respect to DNA repair status or immune capability.

1.1 SOLACE: A phase 1 study of olaparib (O) + LDCy (C) in HGSOC and TNBC/BRCA1/2 BC

Of relevance for SOLACE2, which is designed to address whether prior priming with olaparib/LDCy (SOLACE) improves outcomes with subsequent olaparib/durvalumab treatment, is preliminary data from the SOLACE study.(16) Eligible patients with relapsed HGSOC or TNBC or BRCA1/2 mutant BC, received O+C with a dose escalation strategy using a 3+3 design with cohort expansions once maximal tolerated dose (MTD) was determined. Dose level 1 (DL1); O, 300 mg bid continuously, C, 50mg on days 1,3 and 5 weekly, 21 day cycle. Dose level 2 (DL2); O, 300 mg bid continuously, C, 50mg days 1-5 weekly 21 day cycle. Dose limiting toxicity, defined as grade 4 neutropenia +/- thrombocytopenia, and non-resolving grades 3 and 4 non-haematological adverse events, was evaluated during 1st two cycles. Safety was assessed by CTCAEv5.0 and efficacy with RECISTv1.1 and GCIC criteria.

In HGSOC and BC pts, the recommended phase II dose (DL2: O 300 mg bid continuously, C 50mg on days 1-5 weekly) is tolerable and active, particularly in those with germline BRCA mutation, supporting our hypothesis. Pharmacokinetics of O+C are similar to O in paired comparisons. Amongst the 32 pts (median age 55.8 [range 33-84], 9 had TNBC (*BRCA*1 22%, *BRCA*2 44%) and 23 had HGSOC (*BRCA*1 39%, *BRCA*2 26%).The MTD was dose level 2. Amongst 29 pts who had completed treatment, 16 had 8 cycles of O+C, and 14 continued with O beyond the 8th cycle. Only 1 stopped because of adverse events (AEs) and the remaining 12 stopped O+C due to disease progression. There was no grade (Gr) 4 or 5 AEs. Common AEs were nausea (Gr 1/2: 88%, Gr 3: 3%), fatigue (Gr 1/2: 81%), constipation (Gr 1/2: 38%, Gr 3: 3%), and vomiting (Gr 1/2: 38%, Gr 3: 3 %). 50% required blood transfusion for anaemia.

The median treatment duration of O+C was 4.3 months (0.7-23.5). Unconfirmed disease control rate (DCR) was 73% (N=30; CR=1, PR=9, SD=12). DCR for HGSOC was 81%. GCIG CA125 response rates were 74% and 92% in the ovarian cohort and *BRCA* ovarian cohort respectively.

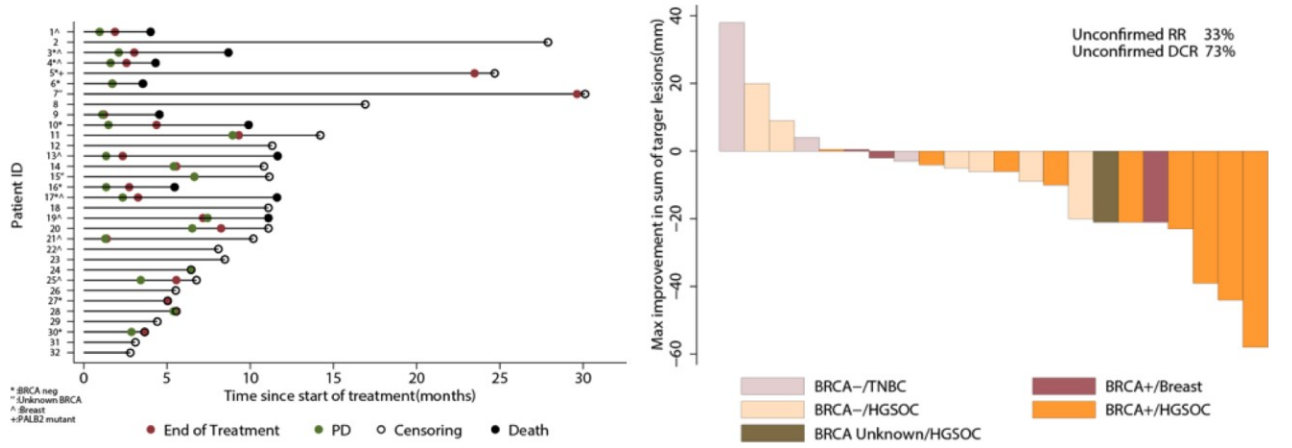

Figure 1. Results of SOLACE: Median PFS BRCA1/2 mutant HGSOC 8.9 months, all HGSOC 6.6 mo; Unconfirmed RECIST RR HGSOC 48%; unconfirmed Ca125 response 70%.

Thus, in HGSOC, the SOLACE data suggests that the combination of olaparib + LDCy is active, particularly in HR defective HGSOC. By extension, we propose that use of LDCy at first relapse (where it is not currently used) in combination with olaparib, in women with HR-defective OC, could improve subsequent response to check-point inhibitor therapy by immunological “priming” of the tumour. This would be predicted to increase the proportion of women with OC who would derive clinical benefit from check-point inhibitor therapy. We hypothesize that the combination of olaparib and LDCy will provide more effective “priming” than priming provided by olaparib alone, addressed by our study design.

1.2 Role of Immunological Priming:

The use of low dose chemotherapy to modulate or prime the immune system to aid in killing the tumour has been best studied for LDCy).(19) We describe a **new property of LDCy** as an agent which can **selectively increase beneficial effector CD4 and CD8 T cell migration, without**

increasing suppressive Treg migration, into the ovarian cancer micro-environment in a subset of patients who go on to survive longer as a result of LDCy than do non-responders. LDCy can prime the immune system to enrich the OC environment in effector CD4 and CD8 T cells (**Figure 2B**), ready for their activity to be further enhanced locally, e.g. by subsequent exposure to immune checkpoint inhibitors. LDCy would therefore be expected in the clinical setting to be a potent priming or adjuvant therapy for immune checkpoint inhibitors and/or cancer vaccines.

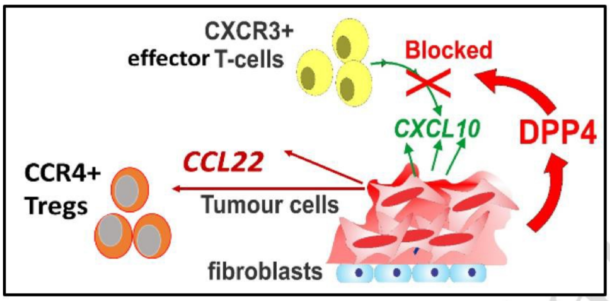

Figure 2A Pre-LDCy treatment
CCR4+ suppressor Tregs migrate towards CCL22; CXCR3+ T effectors (CD4 and CD8 T cells) are blocked from migrating by CXCL10 cleavage mediated by OC cells.

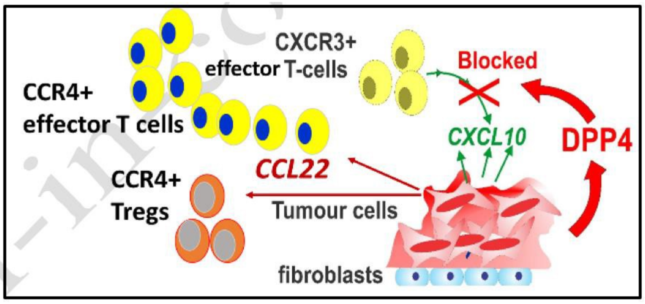

Figure 2B. LDCy effect
Effector CCR4+ CD4 and CD8 T cells migrate into OC environment leading to increased accumulation of beneficial effector CD8 and CD4 T cells.

We propose an initial priming period of three months of olaparib+LDCy in patients with HR-defective OC, to improve the ratio of beneficial effector T cells to Treg within the OC, in order to improve subsequent response to combination olaparib plus checkpoint inhibitor therapy with durvalumab. The rationale for the 12 week lead in with LDCy is that our preliminary data suggests that some patients may require longer than 2-8 weeks of priming, indeed up to 10 weeks. **Preliminary data: Timing of CCR4+ spike and relation to overall survival**. Spike of CCR4 positive CD3 T cells was observed from 2 weeks to 10 weeks post initiation of treatment. Kaplan Meier survival revealed extension in median overall survival from 158 days (CCR4 stable) to 497 days (CCR4 spike) ($p<0.05$). *Translational analysis throughout the 12 week priming period will generate detailed information about timing of immune spikes as surrogates for priming.*

PARP inhibitor therapy alone might also be capable of priming the immune system, by causing DNA damage, potentially causing greater mutational burden and expanding the neoantigen expression, leading to a greater immune recognition of the tumour.(14, 15) We hypothesise that combining olaparib + LDCy as a primer will be superior to priming by olaparib alone. We have therefore designed our study to compare whether the addition of LDCy to olaparib during this priming period results in superior priming ability, compared with priming by olaparib alone. *Translational analysis throughout the 12 week priming period and upon progression will generate detailed information about the immune effects of this therapy.*

As the combination therapy with olaparib+LDCy is only proposed for three months duration (rather than six months as in SOLACE), long-term toxicity from olaparib+LDCy is unlikely to be problematic. Toxicity for olaparib/durvalumab is being assessed in on-going studies and was reported as acceptable in women with gynaecological cancer: the recommended phase 2 dose was durvalumab 1,500mg every 4 weeks with olaparib 300 mg BD. No dose-limiting toxicity was recorded with durvalumab plus olaparib.(15)

1.3 Platinum Sensitivity as a Surrogate for DNA Repair Defective Ovarian Cancer

Our rationale is to select patients based on two criteria for platinum selectivity (normalisation of CA125 by the end of chemotherapy; CA125 GCIG progression not sooner than 6 months following last dose of adjuvant therapy) as a surrogate for DNA repair defective OC. This is based on the limits of current methods of molecular screening for DNA repair defective HGSOC. Data from the NOVA trial (20), the ARIEL2 Part 1 trial (21), and ARIEL3 trial (22) of PARPi therapy as maintenance therapy in relapsed HGSOC (as no first line maintenance data is yet available), reveal

that the current HRD tests used in these trials (the Myriad MyChoice panel and the Foundation Medicine LOH assay) do not provide sufficient selection of HRD HGSOC outside BRCA1/2 mutated HGSOC.(20, 23) We will determine the status of known HR repair pathway genes (mutation, and for appropriate genes, promoter methylation) for all cases as part of the translational componentof this study. DNA repair gene status (mutated vs methylated vs HRD by functional genomic tests) will constitute exploratory end-points of the study. The HRD test will be determined at the appropriate time.

1.4 Durvalumab

1.4.1 Non-clinical and Clinical Experience

The non-clinical and clinical experience with durvalumab is fully described in the most current version of the durvalumab Investigator's Brochure (IB).

Durvalumab is a human monoclonal antibody (mAb) of the immunoglobulin G (IgG) 1 kappa subclass that inhibits binding of PD-L1 and is being developed by AstraZeneca/MedImmune for use in the treatment of cancer. As durvalumab is an engineered mAb, it does not induce antibody-dependent cellular cytotoxicity or complement-dependent cytotoxicity. The proposed mechanism of action for durvalumab is interference of the interaction of PD-L1 with PD-1 and CD80.

To date, durvalumab has been given to more than 1800 patients as part of ongoing studies either as monotherapy or in combination with other anticancer agents. Refer to the current durvalumab Investigator's Brochure for a complete summary of non-clinical and clinical information including safety, efficacy and pharmacokinetics.

1.4.2 Safety

1.4.2.1 Overall Risks

Monoclonal antibodies directed against immune checkpoint proteins, such as programmed cell death ligand 1 (PD-L1) as well as those directed against programmed cell death-1 (PD-1) or cytotoxic T-lymphocyte antigen-4 (CTLA-4), aim to boost endogenous immune responses directed against tumour cells. By stimulating the immune system however, there is the potential for adverse effects on other tissues.

Most adverse drug reactions seen with the immune checkpoint inhibitor class of agents are thought to be due to the effects of inflammatory cells on specific tissues. These risks are generally events with a potential inflammatory mechanism and which may require more frequent monitoring and/or unique interventions such as immunosuppressants and/or endocrine replacement therapy. These risks include gastrointestinal AEs such as colitis and diarrhoea, pneumonitis /interstitial lung disease (ILD), renal AEs such as, nephritis and increases in creatinine, hepatic AEs such as hepatitis and liver enzyme elevations, skin events such as rash and dermatitis, endocrinopathies such as hypo- and hyper-thyroidism, hypophysitis, adrenal insufficiency, diabetes mellitus type I and diabetes insipidus, and neurotoxicities such as myasthenia gravis and Guillain-Barre syndrome.

1.4.2.2 Durvalumab Risks

Risks with durvalumab include diarrhoea, colitis, pneumonitis/ILD, endocrinopathies (hypo- and hyper-thyroidism, thyroiditis, type I diabetes mellitus, diabetes insipidus, hypophysitis and adrenal insufficiency), hepatitis/hepatotoxicity/increases in transaminases, neurotoxicities, myasthenia gravis, nephritis/increases in creatinine, pancreatitis, rash/pruritus/dermatitis (including pemphigoid), infusion-related reactions, anaphylaxis, hypersensitivity or allergic reactions, immune thrombocytopaenia, and immune complex disease.

Further information on these risks can be found in the current version of the durvalumab IB.

In monotherapy clinical studies, AEs (all grades) reported very commonly (≥10% of patients) are fatigue, nausea, decreased appetite, dyspnoea, cough, constipation, diarrhoea, vomiting, back pain, pyrexia, abdominal pain, anaemia, arthralgia, peripheral edema, headache, rash, and pruritus. Approximately 8% of patients experienced an AE that resulted in permanent discontinuation of

durvalumab and approximately 5% of patients experienced an SAE that was considered to be related to durvalumab by the study investigator.

The majority of treatment-related AEs were manageable with dose delays, symptomatic treatment, and in the case of events suspected to have an immune basis, the use of established treatment guidelines for immune-mediated toxicity (see the Dosing Modification and Toxicity Management Guidelines in 4.

A detailed summary of durvalumab monotherapy AE data can be found in the current version of the durvalumab IB.

1.5 Hypothesis

Priming of the immune system with low dose oral cyclophosphamide, in combination with olaparib, followed by combination therapy with olaparib plus anti-PD-L1 inhibitor (durvalumab) will result in higher 36-week progression-free survival (PFS) rate than priming with olaparib alone followed by olaparib and durvalumab in women with asymptomatic relapse of platinum sensitive HGSOC.

1.6 Patient Reported Outcomes

The primary aim of treatment in asymptomatic patients with CA125 progression is to delay the time to first subsequent therapy (TFST) which is commenced for symptomatic progression. Ideally, therapy in asymptomatic patients should be well tolerated and not negatively impact on any of the domains of HRQOL and be associated with high compliance.

The PRO endpoints will help interpret the results of the study and in particular provide patient centred information on whether increasing PFS with the study treatments is of value to patients and the trade-offs, particularly with respect to the adverse effects associated with treatment and their impact on HRQOL. There are a number of endpoints which can be used to interpret the impact of study therapy on PFS in all three treatment arms. These include HRQOL during treatment, at progression and after commencing 2nd line treatment when most patients are symptomatic. Indirect measures of patient benefit and acceptability of maintenance therapy include compliance and patient drop outs for adverse events; impact of adverse effects on HRQOL; the time course and grade of adverse events; time to subsequent chemotherapy and impact of progression and further chemotherapy on HRQOL and finally also patient preferences as ultimately the patient needs to determine whether the benefits are worthwhile to her. These questions are all best addressed in the expansion phase of SOLACE2, but will be looked at during the randomised Phase 2 and the findings used to inform the PRO hypotheses and PRO endpoints in the Phase 3 study.

1.6.1 SOLACE2 PRO Endpoints

The participants who are recruited to SOLACE2 will be asymptomatic with CA125 progression with either no measurable disease or small volume disease not requiring chemotherapy. The aim of study treatment is to prolong the progression free interval and delay the start of second line chemotherapy (TFST) without significantly compromising quality of life and ideally maintaining the quality of life at baseline entry. It is expected that there will adverse effects associated with olaparib, durvalumab and LDCy. The most common adverse effects will be fatigue and nausea which will be transient and more common in the 1st two months of treatment and will improve significantly by 12 weeks. It is anticipated that most participants will continue treatment until progression with <10% stopping early in view of adverse effects. Progressive disease will be associated with symptoms that significantly impact on HRQOL and will indicate the need for 2nd line chemotherapy.

2 AIM AND OBJECTIVES

**Aim**	To determine the efficacy and tolerability of three months of olaparib + low dose oral cyclophosphamide followed by olaparib + durvalumab, versus three months of olaparib monotherapy followed by olaparib + durvalumab, versus olaparib monotherapy alone, in asymptomatic women at first

	CA125 progression with platinum sensitive recurrent ovarian, fallopian tube or primary peritoneal cancer.
**Primary objective**	To determine in each treatment arm:<br>1) 36 week progression-free survival rate (36wPFS, death or PD by RECIST 1.1 criteria at 36 weeks)
**Secondary objectives**	2) Objective response (OR = CR or PR) rate (ORR) by RECIST 1.1 or GCIG CA125 criteria<br>3) Progression free survival (PFS) by RECIST 1.1 or GCIG CA125 criteria<br>4) Objective tumour response (OTR = CR or PR) rate (OTRR) and PFS by iRECIST<br>5) Frequency and severity of adverse events (CTCAE v5.0)<br>6) Aspects of health related quality of life (EORTC QLQ-C30, OV28 and MOST)<br>7) Time to starting first subsequent therapy (TFST) or death<br>8) Time to development of symptoms associated with progression, specifically abdominal/gastrointestinal symptoms
**Tertiary and correlative objectives**	9) To determine whether an immune biomarker can be used to reliably and practically identify patients, diagnostically or prognostically, who will derive maximal beneficial effects from PARPi and/or LDCy combination and/or immune therapy.<br>10) To test whether an immune signature of T cell function, can be used to predict tumour response for the PARPi and/or LDCy combination and /or immune therapy.<br>11) A prognostic (pre-treatment) and diagnostic (post-treatment) Cyclophosphamide-UPregulation (CUP) test may practically identify responders to PARPi and/or LDCy combination and /or immune therapy.<br>12) To test whether genomics analyses of the baseline tumour or post-progression biopsy and/or additional immunological analyses not restricted to T cell function can be used to predict tumour response for the PARPi and/or LDCy combination and /or immune therapy, and to predict the consequences of combined or sequential application of these therapies.<br>13) To test whether baseline tumour PDL1 expression on immunohistochemistry is predictive of benefit with durvalumab therapy<br>14) To test whether change in ctDNA from baseline could be a valid surrogate marker for treatment benefit

3 DESIGN

This is an open-label, randomised, 3-arm, multicentre phase II clinical trial. Participants will be allocated to 3 treatment groups in a ratio of 1:1:1.

Stratification factors are:

Site/Institution
Platinum-free interval (6-12 months vs. >12 months)
CA-125 (<100 U/mL vs. ≥ 100 U/mL)
Measureable disease (yes vs. none)
Germline BRCA mutation status (mutant vs. wild-type)

4 STUDY POPULATION

Patients must meet all of the inclusion criteria and none of the exclusion criteria to be eligible for this trial. No exceptions will be made to these eligibility requirements at the time of randomisation. All enquiries about eligibility should be addressed by contacting the NHMRC CTC prior to randomisation.

4.1 Target Population

The target population is women with high-grade serous carcinoma of the ovary, fallopian tube or primary peritoneum, with features of platinum sensitivity, at first asymptomatic CA125 progression.

4.2 Inclusion Criteria

1. Women, aged 18 years and older, with histologically confirmed high-grade serous carcinoma of the ovary, fallopian tube or primary peritoneum.
2. Undergone prior adequate debulking surgery, as appropriate, and 1 line of platinum-based chemotherapy. Patients who received bevacizumab or hormonal therapy as part of first-line treatment are eligible.
3. Known germline BRCA1 and BRCA2 status. Both BRCA mutant and BRCA wild-type patients are eligible, but they must have normalisation of CA125 to less than upper limit of normal (ULN), defined as 35 units/mL, by the end of first-line chemotherapy.
4. CA125 progression after first-line treatment, occurring 6 months or more following the last dose of chemotherapy. Participants must have raised CA125 readings twice the ULN (≥ 70 kU/L) on 2 occasions at least 1 week apart. The following circumstances are also considered eligible:
    - If CA125 is <70 kU/L, patient must have evaluable disease on imaging (RECIST measurable or non-measurable);
    - In the absence of RECIST measurable disease, CA125 progression (≥ 70 kU/L) after first-line treatment occurring 4 months or more following last dose of chemotherapy is allowed.
5. No indication for immediate chemotherapy or secondary debulking surgery.
6. ECOG performance status of 0-1 (see Appendix 1).
7. Confirmation of the availability of tumour tissue (FFPE) for translational studies (a cell pellet from ascites or pleural fluid is not acceptable). If no tissue is available, participants will not be eligible for this study
8. Adequate bone marrow function (measured within 28 days prior to randomisation and with values within the ranges specified below):
    - Absolute neutrophil count ≥ 1.5×10^9/L
    - Platelet count ≥ 100×10^9/L

- Haemoglobin ≥ 100 g/L with no blood transfusion in the past 28 days
- Peripheral blood smear must not show any features suggestive of myelodysplastic syndrome/acute myeloblastic leukaemia.

9. Adequate liver function, as follows:
   - Total bilirubin ≤ 1.5 x institutional upper limit of normal (ULN)
   - Alkaline phosphatase (ALP), AST (SGOT), ALT (SGPT ≤ 2.5 x ULN (or ≤ 5 x ULN if liver metastases are present)
10. Adequate renal function, as follows:
    - Serum creatinine ≤ 1.5 x ULN, or
    - Creatinine clearance > 50mL/min (Cockcroft-Gault Formula, see Appendix 2)
11. Willing and able to comply with all study requirements, including treatment, timing and/or nature of required assessments.
12. Signed, written informed consent prior to any study specific procedures.

4.3 Exclusion Criteria

1. Previous enrolment or randomisation in the present study.
2. Any previous treatment with a PARP inhibitor, including olaparib.
3. Any previous treatment with durvalumab, or any other anti-PD-1, anti-PD-L1, anti-PD-L2, anti-CTLA-4 antibodies or any other antibody or drug specifically targeting T cell co-stimulation or immune checkpoint pathways.
4. Concomitant use of known strong CYP3A inhibitors (e.g. itraconazole, telithromycin, clarithromycin, protease inhibitors boosted with ritonavir or cobicistat, indinavir, saquinavir, nelfinavir, boceprevir, telaprevir) or moderate CYP3A inhibitors (e.g. ciprofloxacin, erythromycin, diltiazem, fluconazole, verapamil). The required washout period prior to starting olaparib is 2 weeks.
5. Concomitant use of known strong (e.g. phenobarbital, enzalutamide, phenytoin, rifampicin, rifabutin, rifapentine, carbamazepine, nevirapine and St John's Wort) or moderate CYP3A inducers (e.g. bosentan, efavirenz, modafinil). The required washout period prior to starting olaparib is 5 weeks for enzalutamide or phenobarbital and 3 weeks from the last study dose for other agents.
6. Treatment with any investigational product during the last 14 days (or a longer period depending on the defined characteristics of the agents used).
7. Participants receiving any systemic anti-cancer treatment (including endocrine therapy, chemotherapy or VEGF-targeted agents for cancer relapse) or radiotherapy (except for palliative reasons), within 14 days from the last dose prior to study treatment (or a longer period depending on the defined characteristics of the agents used).
8. Participants who are unable to swallow orally administered medication or have gastrointestinal disorders likely to interfere with absorption of the study medication (e.g. including but not limited to partial bowel obstruction or chronic malabsorption syndrome, Crohn's disease and ulcerative colitis).
9. Suspected brain or leptomeningeal metastases, untreated brain metastases or current clinical or radiological progression of known brain metastases, or requirement for steroid therapy for brain metastases. Participants with treated brain metastases are eligible if they have been stable and off steroids for ≥ 3 weeks.
10. Participants with known haemorrhagic cystitis.

11. No history of other active malignancy.
12. History of known or suspected auto-immune disease other than vitiligo, type 1 diabetes, residual hypothyroidism due to an autoimmune condition requiring only hormone replacement, or psoriasis not requiring systematic treatment within the last 2 years.
13. Prior allogeneic organ transplant, double umbilical cord blood transplantation, inflammatory bowel disease (e.g. Crohn's disease, ulcerative colitis), pneumonitis, tuberculosis, or primary immunodeficiency.
14. Participants with prior diagnosis of myelodysplastic syndrome or acute myeloid leukaemia. Any abnormal blood films at baseline need to be reviewed to exclude these conditions.
15. Current or prior use of immunosuppressive medication within 28 days before the first dose of durvalumab, with the exception of intranasal and inhaled corticosteroids or systemic corticosteroids at physiological doses, which are not to exceed 10 mg/day of prednisone or an equivalent corticosteroid. In the case of short term use of systemic corticosteroids (less than 24 hours within 28 days) of greater than 10 mg/day of prednisone or an equivalent corticosteroid, the required washout period prior to starting the first dose of durvalumab is 7 days.
16. Receipt of live attenuated vaccination within 30 days before the first dose of durvalumab.
17. Whole blood transfusions in the last 120 days prior to entry to the study (packed red blood cells and platelet transfusions are acceptable outside of 28 days prior to randomisation).
18. Participants with documented serologically positive hepatitis B or C (defined as positive result for hepatitis B core antibody or hepatitis C antibody).
19. Participants who are known to be serologically positive for human immunodeficiency virus (HIV).
20. Within 28 days of randomisation, participants must have two ECG assessments within a 24 hour period. Participants must not have a resting ECG with a QTc interval of >470 msec or a family history of long QT syndrome.
21. Participants considered a poor medical risk due to a serious, uncontrolled medical disorder, non- malignant systemic disease or active, uncontrolled infection. Examples include, but are not limited to, symptomatic congestive heart failure, uncontrolled hypertension, uncontrolled ventricular arrhythmia, recent (within 3 months) myocardial infarction, unstable angina pectoris, uncontrolled major seizure disorder, unstable spinal cord compression, superior vena cava syndrome, extensive interstitial bilateral lung disease on High Resolution Computed Tomography (HRCT) scan, active peptic ulcer disease or gastritis, active bleeding diathesis, or any psychiatric disorder/social situation that would limit compliance with study requirements or compromise the ability of the subject to give written informed consent.
22. Major surgical procedure within 14 days prior to study treatment, or still recovering from any effects of major surgery.
23. Any unresolved toxicity NCI CTCAE Grade >1 from previous anticancer therapy. Subjects with Grade ≤2 neuropathy or Grade ≤2 alopecia are exceptions to this criterion.
24. Participants with known hypersensitivity to cyclophosphamide or any excipients of olaparib, cyclophosphamide or durvalumab.
25. Life expectancy of less than 12 months.
26. Pregnancy, lactation, or inadequate contraception. Most participants would have undergone prior oophorectomy and hysterectomy as part of surgical treatment for ovarian cancer. For women who have ovaries and uterus in situ, they must be post-menopausal, infertile, or use a reliable means of contraception. Women of childbearing potential must have a negative pregnancy test done within 7 days prior to randomisation.

4.4 *Study Enrolment*

4.4.1 Screening

Written informed consent must be signed and dated by the participant, and signed and dated by the Investigator, prior to any study-specific screening investigations being performed.

4.4.2 Registration and Randomisation

Registration and randomisation must be done according to the instructions in the Study Manual.

Requests for randomisation will only be accepted from authorised investigators at sites that have all requisite approvals in place. Randomisation should be done only after all screening assessments have been performed and the responsible investigator has verified the participant's eligibility.

Participants will be centrally randomised through the NHMRC CTC. The method of randomisation is a stratified dynamic allocation approach, randomly alternating between minimisation and complete randomisation. The strata are: Site/Institution; Platinum-free interval (6-12 months vs. >12 months); CA-125 (<100 U/mL vs. ≥ 100 U/mL); Measureable disease (yes vs. none); and Germline BRCA mutation status (mutant vs. wild-type). Participants must be randomised before starting study treatment. Treatment should be planned to start as soon as possible and within 7 days after randomisation. The baseline research blood must be confirmed as adequate prior to treatment commencement, as described in the Biospecimen Sampling Manual.

Once the randomisation process has been completed, the participant will be assigned a study number, a treatment arm, and written confirmation of successful randomisation will be provided to the site.

Individuals may only be randomised once in this trial.

5 TREATMENT PLAN

All arms in this study are interventional. Durvalumab, with and without immunological "priming" with low dose cyclophosphamide (LDCy), will be administered to participants in Arms A and B. All participants in all three arms (A, B and C) will receive olaparib until unequivocal disease progression.

5.1 *Treatments*

All three drugs, olaparib, durvalumab and LDCy are study interventions in this trial.

5.1.1 Study Treatment

Participants will be randomised to one of three arms (Tables 1 and 2). Treatment will be administered in 4-week cycles throughout the Priming and Consolidation and Maintenance phases.

Table 1. Treatment schema

	Priming phase			Consolidation phase												Maintenance phase
Cycle (4 weeks)	1	2	3	4	5	6	7	8	9	10	11	12	13	14	15-39	Until unequivocal PD
Arm A	O	O	O	O+D	O+D	O+D	O+D	O+D	O+D	O+D	O+D	O+D	O+D	O+D	O+D	O
Arm B	O+C	O+C	O+C	O+D	O+D	O+D	O+D	O+D	O+D	O+D	O+D	O+D	O+D	O+D	O+D	O
Arm C	O	O	O	O	O	O	O	O	O	O	O	O	O	O	O	O

O = olaparib, D = durvalumab, C = cyclophosphamide, PD = progressive disease

Table 2. Study treatments

Agent(s)	Dose	Route	Duration	Arms
Olaparib	300 mg BD	PO	Until unequivocal disease progression or unmanageable toxicity	All arms
Cyclophosphamide	50mg daily for days 1-5 consecutively each week	PO	3 cycles during Priming phase (in the absence of unequivocal disease progression or unmanageable toxicity)	Arm B only
Durvalumab*	1500mg* on day 1 of every 4 week cycle	IV	36 cycles during Consolidation phase (in the absence of unequivocal disease progression or unmanageable toxicity)	Arms A and B only

*If a participant's weight is ≤ 30kg at baseline or during study, this participant should receive weight-based dosing with durvalumab 20 mg/kg Q4W. If the same participant's weight improves to >30 kg, fixed dosing of durvalumab 1500mg is recommended.

5.1.2 Required Background Treatment

There is no required background medication or premedication.

5.1.3 Olaparib

Participants will be administered olaparib orally twice daily at 300 mgs bid continually. Olaparib tablets should be taken at the same time each day, approximately 12 hours apart with one glass of water. The olaparib tablets should be swallowed whole and not chewed, crushed, dissolved or divided. Olaparib tablets can be taken with or without food.

If vomiting occurs shortly after the olaparib tablets are swallowed, the dose should only be replaced if all of the intact tablets can be seen and counted. Should any participant enrolled on the study miss a scheduled dose for whatever reason (e.g., as a result of forgetting to take the tablets or vomiting), the participant will be allowed to take the scheduled dose up to a maximum of 2 hours after that scheduled dose time. If greater than 2 hours after the scheduled dose time, the missed dose is not to be taken and the participant should take their allotted dose at the next scheduled time.

5.1.3.1 Renal Impairment

If subsequent to study entry and while still on study therapy, a participant's estimated CrCl falls below the threshold for study inclusion (≥51 ml/min), retesting should be performed promptly.

A dose reduction is recommended for participants who develop moderate renal impairment (calculated creatinine clearance by Cockcroft-Gault equation of between 31 and 50 ml/min) for any reason during the course of the study: the dose of olaparib should be reduced to 200mg BD.

Because the CrCl determination is only an estimate of renal function, in instances where the CrCl falls to between 31 and 50 mL/min, the investigator should use his or her discretion in determining whether a dose change or discontinuation of therapy is warranted.

Olaparib has not been studied in patients with severe renal impairment (creatinine clearance ≤ 30 ml/min) or end-stage renal disease; if participants develop severe impairment or end stage disease is it recommended that olaparib be discontinued.

5.1.4 Durvalumab Administration

Durvalumab will be administered by controlled infusion pump into a vein after warming to room temperature (approximately 25°C).

Following preparation of durvalumab, the entire contents of the IV bag should be administered as an IV infusion over approximately 60 minutes using a 0.2-μm in-line filter. The IV line must be flushed with a volume of normal saline (equal to the priming volume of the infusion set used) after the contents of the IV bag are fully administered, or complete the infusion according to institutional policy to ensure the full dose is administered. Documentation must be made if the line was not flushed.

Durvalumab may be infused using either normal saline 0.9% [weight/volume] sodium chloride for injection or 5% [weight/volume] dextrose. The durvalumab solution should not be infused through an IV line in which other solutions or medications are being administered.

5.1.5 Monitoring of Durvalumab Administration

Participants will be monitored during and after the infusion with assessment of vital signs at the times specified in the schedule of study assessments.

As with any antibody, allergic reactions to dose administration are possible. Appropriate drugs and medical equipment to treat acute anaphylactic reactions must be immediately available, and study personnel must be trained to recognise and treat anaphylaxis. The study site must have immediate access to emergency resuscitation teams and equipment in addition to the ability to admit participants to an intensive care unit if necessary.

The management of infusion-related reaction is summarised in Appendix 3.

5.2 Dose Modifications

Instructions for treatment delays and dose modifications for adverse events (AEs) are specified below. In general, treatment should be withheld during adverse events of severity Grade 3-4 (according to the Common Terminology Criteria for Adverse Events (CTCAE) v5.0), and not restarted until the adverse event has resolved to Grade 0-1, at the investigator's discretion. Day 1 treatment may be delayed for a maximum of 28 consecutive days. If the adverse event has not resolved to G0-1 after delaying day 1 treatment for 28 consecutive days, then study treatment should be discontinued. Treatment should not be delayed or modified for alopecia of any grade.

Specified dose reductions apply to all subsequent doses of study drug. If a participant experiences several adverse events with differing recommendations, then the modification that results in the longest delay and lowest dose should be used.

In cases of delayed treatment, the research blood sample that has been collected as per the original scheduled collection time will still be sent for analysis. An additional research blood sample must be collected within 3 days prior to the rescheduled treatment.

5.2.1 Dose Levels

Table 3. Dose levels

DRUG(S)	Units	STARTING DOSE	LEVEL			
			-3	-2	-1	0 START
Cyclophosphamide	(mg)	50	50, Day 1, 3 and 5 every week	50, Days 1, 3 and 5 every week	50, Days 1, 3 and 5 every week	50, Day 1 to 5 every week
Olaparib	(mg)	300	200	250	300	300

Dose reductions and dose escalations of durvalumab are not permitted in this trial.

5.2.2 Dose Modifications for Adverse Events

For participants on combination therapy, if the observed AE is specifically attributed to only 1 of the drugs, that drug may be held while the participant continues to receive the drug(s) not associated with the observed AE. The PI will determine whether one or both drugs are responsible for an observed toxicity and will manage that toxicity as described below. The time a given drug is held should not exceed 28 days.

5.2.2.1 Olaparib and Cyclophosphamide

5.2.2.1.1 Haematological AEs

At any stage, treatment can only continue if haemoglobin ≥80 g/L, ANC ≥1.0x10^9/L and platelets ≥75x10^9/L. Haematological AEs are typically associated with olaparib and/or cyclophosphamide and are not anticipated with durvalumab. These dose modifications will apply to participants receiving treatment with olaparib, with or without cyclophosphamide, in all arms at any phase of treatment (Table 4).

Investigations and supportive care for haematological AEs (including growth factor support and blood product transfusions) should be initiated as per local institutional practice.

Table 4. Management of olaparib and cyclophosphamide for haematological AEs

Parameter	Timing	Result or Problem	CTCAE Grade	Action with Study Treatment
Neutrophils (1.0x10^9/L)	Any stage	0.5-1.0	G3	First occurrence: Delay olaparib (and cyclophosphamide) for up to 28 days until ≥1.0. Restart treatment at the same dose level.<br>Second occurrence: Delay olaparib (and cyclophosphamide) for up to 28 days until ≥1.0. Restart treatment with dose reduction by 1 dose level.
	Any stage	<0.5	G4	First occurrence: Delay olaparib (and cyclophosphamide) for up to 28 days until ≥1.0. Restart treatment with dose reduction by 1 dose level.

				Second occurrence: Delay olaparib for up to 28 days until ≥1.0. Restart treatment with olaparib with dose reduction by 1 level. Discontinue cyclophosphamide if in Arm B.<br>Third occurrence: Discontinue olaparib.
Platelets<br>(x10^9/L)	Any stage	50-75	G2	First occurrence: Delay olaparib (and cyclophosphamide) for up to 28 days until platelets ≥75. Restart treatment at the same dose level.<br>Second occurrence: Delay olaparib (and cyclophosphamide) for up to 28 days until platelets ≥75. Restart treatment with dose reduction by 1 dose level.
	Any stage	<50 or >50 with bleeding	G3-4	First occurrence: Delay olaparib (and cyclophosphamide) for up to 28 days until platelets ≥75. Restart treatment with dose reduction by 1 dose level.<br>Second occurrence: Delay olaparib for up to 28 days until platelets ≥75. Restart treatment with olaparib with dose reduction by 1 level. Discontinue cyclophosphamide if in Arm B.<br>Third occurrence: Discontinue olaparib
Haemoglobin (g/L)	Any stage	<80	G3	First occurrence: Delay olaparib (and cyclophosphamide) for up to 28 days until haemoglobin ≥100. Restart treatment at the same dose level.<br>Second occurrence: Delay olaparib (and cyclophosphamide) for up to 28 days until haemoglobin ≥100. Restart treatment with dose reduction by 1 dose level.
	Any stage	<65	G3	First occurrence: Delay olaparib (and cyclophosphamide) for up to 28 days until haemoglobin ≥100. Restart treatment with dose reduction by 1 dose level.<br>Second occurrence: Delay olaparib for up to 28 days until haemoglobin ≥100. Restart treatment with olaparib with dose reduction by 1 dose level. Discontinue cyclophosphamide if in Arm B.<br>Third occurrence: Discontinue olaparib
Day 1 treatment may be delayed for a maximum of 28 consecutive days. If the adverse event has not resolved to G0-1 after delaying day 1 treatment for 28 consecutive days, then treatment should be discontinued.				

Prolonged haematological toxicities are defined as:

- 28 days of interruption/delay in study treatment due to grade 3 or worse anaemia and/or development of blood transfusion dependence
- > 28 days interruption/delay in study treatment due to grade 3 or worse neutropenia (ANC < $1.0x10^9/L$)
- ≥ 28 days interruption/delay in study treatment due to grade 3 or worse thrombocytopenia (Platelets < $50x10^9/L$)

If prolonged haematological toxicities while on study treatment, weekly differential blood counts including reticulocytes (calculate reticulocyte index (RI), RI = reticulocyte count x hematocrit (Hct)/normal Hct; a value of 45 is usually used for normal Hct) and peripheral blood smear should be performed. If any blood parameters remain clinically abnormal after 4 weeks of dose interruption, the participant should be referred to haematologist for further investigations. Bone marrow analysis and/or blood cytogenetic analysis should be considered at this stage according to standard haematological practice.

Development of a confirmed myelodysplastic syndrome or other clonal blood disorder should be reported as an SAE. Study treatment should be discontinued if diagnosis of myelodysplastic syndrome is confirmed.

5.2.2.1.2 Non-haematological AEs

Guidelines are provided here regarding dose modification for non-haematological AEs associated with olaparib (and cyclophosphamide) (Table 5). Dose modifications for nausea, vomiting, and diarrhoea will be made only if they are refractory to treatment. Asymptomatic electrolyte abnormalities with optimal repletion will not require dose reduction if resolution to grade 1 or less is documented within 4 days after onset of the event.

Because the AEs related to olaparib may include asthenia, fatigue and dizziness, participants should be advised to use caution while driving or using machinery if these symptoms occur.

The following antiemetic regimen is recommended in cases of nausea for all participants:

- Metoclopromide 10mg PO qid prn D1-28;
- Steroids and 5HT3 antagonists are not recommended as routine antiemetics, but may be used as required to manage incidental nausea and/or vomiting.

Table 5. Management of olaparib and cyclophosphamide for non-haematological AEs

CTCAE Grade	Management
≤Grade 1	Proceed with treatment<br>No dose adjustment
Grade 2 affecting ADLs for ≤7 days	Stop treatment<br>Delay recommencement of treatment until toxicity has recovered to ≤Grade 1<br>Maintain current dose level
Grade 2 affecting ADLs for >7 days	Stop treatment<br>Delay recommencement of treatment in until toxicity has recovered to ≤Grade 1<br>≤7 days delay: Maintain current dose level<br>>7 days delay: Dose reduce by 1 dose level
≥Grade 3	Stop treatment<br>Delay recommencement of treatment until toxicity has recovered to ≤Grade 1

	≤7 days delay: Dose reduce by 1 dose level<br>>7 days delay: Take participant off study
Day 1 treatment may be delayed for a maximum of 28 consecutive days. If the adverse event has not resolved to G0-1 after delaying day 1 treatment for 28 consecutive days, then treatment should be discontinued.	

If new or worsening pulmonary symptoms (e.g. dyspnoea) or radiological abnormality occurs, an interruption in olaparib dosing is recommended and a diagnostic workup (including a high resolution CT scan) should be performed to exclude pneumonitis. Following investigation, if no evidence of abnormality is observed on CT imaging and symptoms resolve, then olaparib treatment can be restarted, if deemed appropriate by the investigator. Referral to a respiratory physician and consideration of high dose corticosteroids should take place in the event of abnormalities on CT scan or continuing symptoms.

Study drugs should be stopped at least 3 days prior to planned surgery. After surgery, study treatment can be restarted when the wound has healed. No stoppage of study drugs is required for any biopsy or other minor procedures (such as abdominal paracentesis), provided that blood counts are satisfactory for these procedures to be performed safely. If surgery is required due to reasons related to disease progression, participants should be withdrawn from the study treatment.

Study drugs should be discontinued for a minimum of 7 days before a participant undergoes therapeutic radiation treatment. This is not required where palliative radiation doses are used. Study drugs should be stopped and the participant withdrawn from treatment if radiation is administered due to disease progression.

5.2.2.2 Durvalumab

5.2.2.2.1 Immune-related AEs

Dose reductions and dose escalations of durvalumab are not permitted in this trial. See section Appendix 4 for dose delay for immune-related adverse events.

5.2.2.2.2 Non-Immune-Mediated AEs

Dose modifications are not required for adverse events not deemed to be related to durvalumab (i.e. events due to underlying disease) or for laboratory abnormalities not deemed to be clinically significant. Guidelines are provided here for non-immune-mediated AEs associated with durvalumab (Table 6). Participants should be treated accordingly as per institutional standard.

Table 6. Management of durvalumab for non–immune-mediated AEs

CTCAE grade	Management
Grade 1	No dose modifications.
Grade 2	Hold durvalumab until resolution to ≤ grade 1 or baseline
Grade 3	Hold durvalumab until resolution to ≤ grade 1 or baseline<br>For AEs that downgrade to ≤ grade 2 within 7 days or resolve to ≤ grade 1 or baseline within 28 days, resume durvalumab administration at next scheduled dose. Otherwise, discontinue durvalumab.
Grade 4	Discontinue durvalumab (Note: for grade 4 AEs, the decision to discontinue would be based on accompanying clinical signs/symptoms and as per Investigator's clinical judgment and in consultation with the study chair.

5.3 *Post-study treatment*

All participants will continue treatment with olaparib in the absence of disease progression or unmanageable toxicity. Olaparib will be provided as trial stock while the trial is open. After the trial is closed, participants will be provided with olaparib free of charge by the manufacturer until the product is available to the public.

Any other treatment after permanent discontinuation of study treatment is at the discretion of the participant's clinician.

5.4 *Concomitant Medications*

5.4.1 Recommended

Investigators may prescribe concomitant medications or treatments (e.g. paracetamol, opiate analgesics, diphenhydramine) deemed necessary to provide adequate prophylactic or supportive care except for those medications identified as "excluded" as listed in Section 5.4.2.

5.4.2 Prohibited

The following medications should not be used during this study. Participants who require treatment with any of these agents will usually need to discontinue study treatment. This should be discussed with the Study Chair by contacting the NHMRC CTC:

- Other concurrent investigational treatments or anti-cancer therapy.
- Potent inhibitors of CYP3A4, including ketoconazole, itraconazole, ritonavir, idinavir, saquinavir, telithromycin, clarithromycin and nelfinavir. For participants taking any of the above, the required wash-out periods prior to starting olaparib is two week. See Appendix 5 for full list.
- Potent inducers of CYP3A4, including Phenytoin, rifampicin, rifapentin, rifabutin, carbamazepine, phenobarbitone, nevirapine, modafinil and St John's Wort. For participants taking any of the above, the required wash-out periods prior to starting olaparib are 5 weeks for phenobarbitone and 3 weeks for any of the others. See Appendix 5 for full list.
- Corticosteroids (>10mg daily prednisone or equivalent dose of an alternative corticosteroid) or other immunosuppressive medications should be avoided unless they are necessary:
    - To treat severe adverse events, e.g. autoimmune phenomena, or brain metastases developed during study
    - As a temporary measure for other indications at the discretion of the PI (e.g. chronic obstructive pulmonary disease, radiation, nausea, etc.).
- Live attenuated vaccines (including shingles/ herpes zoster vaccine) cannot be given <30 days before the first dose of durvalumab, during treatment with durvalumab and for 30 days post discontinuation of durvalumab). Inactivated viruses such as those in the influenza vaccine are permitted.
- Herbal medications (note: probiotics are not considered herbal medications).
- It is not recommended for participants to consume grapefruit juice while on olaparib therapy.

5.4.3 Use with Caution

The following medications should be avoided whilst participants are on study drug, and must be used with caution:

- Participants who are taking warfarin may participate in this study. However, it is recommended that prothrombin time (INR and APTT) be monitored carefully at least once per week for the first month, then monthly if the INR is stable. Subcutaneous heparin or clexane is permitted.

- Caution should be exercised in the concomitant use of any medication that may markedly affect renal function. Such medications may be used with caution as deemed essential for treatment, or if already in use prior to entry in the study without any effect on renal function.
- P-gp inhibitors. It is possible that co-administration of P-gp inhibitors (eg amiodarone, azithromycin) may increase exposure to olaparib. Caution should therefore be observed.
- Based on limited in vitro data, olaparib may increase the exposure to substrates of CYP3A4, P-gp, OATP1B1, OCT1, OCT2, OAT3, MATE1 and MATE2K. Based on limited in vitro data, olaparib may reduce the exposure to substrates of CYP3A4, CYP1A2, 2B6, 2C9, 2C19 and P-gp. The efficacy of hormonal contraceptives may be reduced if co administered with olaparib. Caution should therefore be observed if substrates of these isoenzymes or transporter proteins are co-administered. Examples of substrates include:
    - CYP3A4 – hormonal contraceptive, simvastatin, cisapride, cyclosporine, ergot alkaloids, fentanyl, pimozide, sirolimus, tacrolimus and quetiapine
    - CYP1A2 – duloxetine, melatonin
    - CYP2B6 – bupropion, efavirenz
    - CYP2C9 – warfarin
    - CYP2C19 - lansoprazole, omeprazole, S-mephenytoin
    - P-gp - simvastatin, pravastatin, digoxin, dabigatran, colchicine
    - OATP1B1 - bosentan, glibenclamide, repaglinide, statins and valsartan
    - OCT1, MATE1, MATE2K – metformin
    - OCT2 - serum creatinine
    - OAT3 - frusemide, methotrexate

5.4.4 Concomitant Medication Reporting

Concomitant medications will not be recorded in the eCRF routinely. Only concomitant medications administered within 30 days prior to a serious adverse event will be recorded as part of the serious adverse event report. Medications used to treat the SAE are not required to be recorded.

5.5 Restrictions During the Study

5.5.1 Contraception

Participants of child bearing potential, who are sexually active, must agree to the use of two highly effective forms of contraception throughout their participation in the study and for 3 months after the last dose of study drugs.

Acceptable non-hormonal birth control methods include:

- Total sexual abstinence. Abstinence must be for the total duration of the study and the drug washout period
- Vasectomised sexual partner plus male condom, with participant assurance that partner has received post-vasectomy confirmation of azoospermia
- Tubal occlusion plus male condom with spermicide
- Intra-uterine device (IUD) (provided coils are copper-banded) plus male condom plus spermicide

Acceptable hormonal birth control methods include:

- Etonogestrel implants (e.g. Implanon, Norplan) plus male condom with spermicide
- Normal and low dose combined oral pills plus male condom with spermicide
- Norelgestromin/EE transdermal system plus male condom with spermicide
- Intravaginal device plus male condom with spermicide (e.g. EE and etonogestrel)

Cerazette (desogestrel) plus male condom with spermicide. Cerazette is currently the only highly efficacious progesterone based pill

5.6 Treatment Compliance

Participant compliance with olaparib and cyclophosphamide can be determined at each clinic visit by reviewing dosing sheets. Additional measures may be considered if needed. The participant should be counselled appropriately if significant non-compliance is determined.

5.7 Study Treatment Discontinuation

Study treatment will be permanently discontinued for any of the following reasons:

- Unequivocal progression, which is defined as evidence of disease progression and investigator assessment that the participant is no longer benefitting from treatment.

  Note. In the event of progression by RECIST, treatment may be continued for patient benefit at the discretion of the Investigator in consultation with the Chief Investigator (event noted as PD). If a participant in Arms A or B has disease progression during the maintenance phase (while receiving olaparib alone) more than 12 months after ceasing durvalumab, they may be considered for recommencement of durvalumab after discussion with the study chair. Participants who continue study treatment beyond disease progression must continue to follow the on-treatment assessments schedule, including the CT-CAP scans.

- Unacceptable toxicity as determined by the participant or site investigator or as defined in Section 5.2.2
- Grade ≥ 3 infusion reaction
- Development of myelodysplastic syndrome of acute myeloid leukaemia
- The PI determines that continuation of treatment is not in the participant's best interest.
- Occurrence of an exclusion criterion affecting participant safety, e.g. pregnancy or psychiatric illness.
- Required use of a concomitant treatment that is not permitted, as defined in Section 5.4.2
- Failure to comply with the protocol, e.g. repeatedly failing to attend scheduled assessments.
- The participant declines further study treatment, or withdraws their consent to participate in the study.
- Inability to reduce corticosteroid to a dose of ≤10 mg of prednisone per day (or equivalent) **within 12 weeks** after last dose of durvalumab
- Grade 3-4 immune-related AE as outlined in Appendix 4 for durvalumab. Continuation of olaparib could be allowed after discussion with study chair.

The reasons for discontinuing treatment will be documented in the participant's medical record and eCRF.

Participants who stop study treatment will be requested to continue follow-up visits according to the protocol. All participants will be followed until the end of the study.

If a participant wishes to stop the study visits, they will be requested to allow their ongoing health status to be periodically reviewed via phone contact or from their general practitioner, or medical records, state-based cancer registries and/or the national mortality registry (AIHW).

6 ASSESSMENT PLAN

6.1 Schedule of Assessments

	**Screening/ Baseline**	**On Treatment**					**Follow-up After Study Treatment**		
		Priming Phase (weeks 1-12)		Consolidation Phase (weeks 13-156)		Maintenance Phase (week 157 onwards)	Until progression [13]	At progression	After progression / Follow-Up
	Within 28 days prior to randomisation	Within 3 days prior to day 1 of every 4-week cycle	Day 15 of every 4-week cycle (within 3 days prior)	Early consolidation phase (weeks 13-60) Within 3 days prior to day 1 of every 4-week cycle	Late consolidation phase (weeks 61-156) 8-weekly: within 3 days prior to day 1 of every 2nd cycle	8-weekly: within 3 days prior to day 1 of every 2nd cycle	12-weekly +/- 14 days		12-weekly +/- 14 days
Written informed consent	X								
Archival tissue (mandatory)[10]	X								
Medical history	X								
Clinic assessment	X	X[1]		X	X	X	X[13]		
Weight, HR, temp, RR, BP		X		X	X	X			
ECOG performance status	X	X		X	X	X			
HIV and hepatitis screening[2]	X								
Haematology and biochemistry[2]	X	X[1]		X	X	X	X[13]	X	
Coagulation tests[3]	X								
TFTs[4]	X	X[1]		X	X	X	X[13]	X	
CA125 tumour marker	X	X[1]		X	X	X	X	X[14]	X[14]
ECG[5]	X								
Urinalysis[5]	X								
CT chest, abdomen and pelvis	X	From randomisation, at weeks 8 and 24, then 12-weekly[11]							
PRO questionnaires (QLQ C-30, OV28 and MOST)	X	X[1]		X[12]	X	X	X	X[9]	4 weeks after progression
Concomitant medications		Only for SAEs[6]							
(Serious) Adverse Events		X		X	X	X	X[6]	X[6]	X[6]
Bloods for translational research[7]	X[7]	X[7]	X[7]	X[7]	X[7]			X	
Tumour biopsy (optional)[10]	X							X	
Survival and anti-cancer treatments[8]							X		X

Footnotes

1. Repeat only if more than 7 days between baseline screening and the first dose of study treatment.
2. Haematology tests must include a full blood count and differentials. Clinical chemistry tests must include the following: bicarbonate, urea, creatinine, total bilirubin, albumin, total protein, AST, ALT, GGT, ALP, LDH, calcium, magnesium, phosphate, chloride, potassium, sodium, glucose, lipase and uric acid.

   Hepatitis B and C serological testing is required prior to study entry, if not already done previously. HIV testing prior to study entry is only required if clinically indicated and at Investigator's discretion.
3. Coagulation tests, including PT, APTT and INR, are only mandated at screening. They may otherwise be performed if clinically indicated.
4. TFTs include free T3, free T4 and TSH. They are only routinely required during the consolidation and maintenance phases for arms A and B. They should also be measured if there is a clinical suspicion of an adverse event related to the endocrine system in arms A and B.
5. At Screening, participants must have two ECG assessments within a 24-hour period. Subsequently, repeat only if clinically indicated.
6. Until 90 days after cessation of study treatment.
7. Blood samples will be collected from all participants (required) for translational research at up to 26 time-points: baseline, on days 1 and 15 of cycles 1-3 (priming phase), on day 1 of cycles 4-15 (early consolidation phase), on day 1 of every 4th cycle for cycles 16-39 (late consolidation phase) and at progression. The timing of collection (excluding baseline) is within 3 days prior to day 1 of the cycle. Refer to Biospecimen Sampling Manual for processing and storage details.
8. Follow-up should be conducted following study treatment discontinuation every 12 weeks (+/- 14 days) from EOT. Contact by telephone is acceptable.
9. Questionnaires will be administered to participants at time of disease progression, unless questionnaires have been completed in the previous 4 weeks.
10. Archival tissue that is available for testing at central pathology laboratory is mandatory for all participants. Confirmation is required from the pathology laboratory that they hold a block with tumour and will send requested block/slides. For participants who undergo biopsies or surgery (optional) to investigate disease progression prior to randomisation or on study treatment, additional tissue will be requested for translational research.
11. Scans may be performed +/- 7 days of the protocol-scheduled timepoints. Participants who have evidence of progressive disease on a routine CT scan, and who would like to continue Durvalumab treatment on trial, must undergo an additional confirmatory CT scan within 4-8 weeks later to confirm progressive disease.
12. PRO questionnaires are administered at baseline, every 4 weeks for 60 weeks, then every 8 weeks beyond 60 weeks until progression.
13. If a participant discontinues due to reasons other than disease progression (e.g. unacceptable toxicity), Clinic assessment, Haematology, Biochemistry & TFTs can continue at the Investigator's discretion. All other assessments must continue as per protocol until disease progression, the participant has commenced another anti-cancer therapy, or the participant has withdrawn consent.
14. A repeat CA125 test is required to confirm disease progression prior to starting a new anti-cancer therapy, where possible (refer to Appendix 10). The repeat test should be performed at least one week after the initial CA125 where progression was first detected.

6.2 Details of Assessments

6.2.1 Screening phase

Screening procedures will be performed up to 28 days before randomisation, unless otherwise specified. All participants must first read, understand, and sign the HREC approved consent form before any study-specific screening procedures are performed. After signing the consent form, completing all screening procedures, and being deemed eligible for entry, participants will be enrolled in the study. Procedures that are performed prior to the signing of the consent form and are considered standard of care may be used as screening assessments if they fall within the 21-day screening window.

6.2.2 Clinical Assessment

C1D1 must occur within 7 days of randomisation. Subsequent CXD1 visits should be scheduled every 28 days (+/- 3) days from original schedule. Clinical assessments must occur within 3 days prior to D1 of every cycle.

Physical examination

A full physical examination will be performed during screening and will include an assessment of the following: general appearance, respiratory, cardiovascular, abdomen, skin, head and neck, lymph nodes, thyroid, musculoskeletal, and neurological systems and at screening only, height. A targeted physical examination as clinically indicated will be performed on subsequent evaluations. These assessments are not recorded directly in the eCRF.

Vital signs

At the start of every 4-week cycle, vital signs (blood pressure, pulse rate, respiratory rate and temperature) will be measured.

For participants in arms A and B who are being treated with durvalumab during the consolidation phase, vital signs will be measured on the day of first infusion only of durvalumab treatment: within an hour prior to start of durvalumab administration, at 30 minutes during the infusion (+/- 5 minutes) and at the end of infusion (+/- 5 minutes). For subsequent infusions, observations pre- and during infusion should be checked as per institutional standard of care.

Post-infusion, vital signs will also be measured at 30 and 60 minutes after the infusion (i.e. 90 and 120 minutes from the start of the infusion) (+/- 5 minutes) for the first infusion only, and then for subsequent infusions only as clinically indicated. If the infusion takes longer than 60 minutes, then blood pressure and pulse measurements should follow the principles described here or more frequently if clinically indicated. These assessments are not recorded in the eCRF.

Laboratory safety assessment

Any clinically significant abnormal laboratory values should be repeated as clinically indicated.

ECGs

All ECGs for this trial are done using standard techniques using 12 leads. Participants should be supine and have been resting for at least 5 minutes. The same method of assessment should be used throughout the study.

Two ECG assessments within a 24-hour period is required to be recorded during the Screening period (with 2-5 minute lag time between each). Subsequently, ECGs will be repeated only if clinically indicated.

6.2.3 Imaging

Contrast enhanced CT chest, abdomen and pelvis is required for this study. From randomisation, tumour assessment CT scans will be performed at 8 weeks, 24 weeks and then every 12 weeks. Scans may be performed +/- 7 days of the protocol-scheduled timepoints. If a participant is noted to have progressive disease (iUPD by iRECIST) on a routine CT scan and would like to continue Durvalumab treatment on trial, they must undergo a confirmatory CT scan within 4-8 weeks later to confirm progressive disease (iCPD by iRECIST).

CT scans for screening and tumour assessments must be performed using the same machine and settings. Low dose CT imaging is not acceptable.

6.2.4 Quality of Life

Health-related quality of life (HRQLQ) will be assessed using the EORTC QLQ-C30, OV28 and Measure of Ovarian Symptoms and Treatment (MOST) questionnaires (Appendices 6, 7 and 8) at baseline, every 4 weeks for 60 weeks, then every 8 weeks beyond 60 weeks until progression, and then 4 weeks later. Questionnaires will be administered to participants at progression, unless questionnaires have been completed in the previous 4 weeks.

The HRQLQ should be given to participants for completion during their visit, preferably before seeing their oncologist and having their study treatment. Participants should complete the forms without assistance from others. It should be explained to the participant that the assessment covers the QOL during the previous 4 or 8 weeks.

It is recommended that the same member of staff explains and collects QOL forms for this trial to optimise completeness, compliance, and consistency.

6.2.5 Blood Collection

Local pathology laboratories will be used for routine blood tests. Blood will be taken prior to the administration of each cycle.

Blood for translational research will be collected from all participants. Consent for translational research is part of the consent for the study. Blood for research will be collected and couriered to a central laboratory in real-time for translational research studies. See section 9.2 for collection time-points. There are **TWO** separate translational research (TR) blood collections **prior to starting study treatment**. The first TR blood collection is at screening (also referred to as the baseline sample) and should be collected within 28 days **prior** to starting treatment. The second TR blood collection (Cycle 1 Day 1 (C1D1) sample) must be collected within three days **prior** to starting treatment. Thereafter, TR bloods may be taken following any morning doses of Olaparib and Cyclophosphamide. However, they must be collected within 3 days **prior** to the Durvalumab.

If there is a treatment delay, TR bloods are to be collected as per the schedule, relative to the participant’s last dose administration, with an additional re-collect of the same time point within 3 days prior to the rescheduled treatment cycle.

Refer to the Biospecimen Sampling Manual for procedures.

6.2.6 Tissue Collection

Archival formalin-fixed paraffin-embedded tumour tissue must be available for translational studies from all participants at baseline (eligibility).

Separate participant consent will be sought using a tick-box for additional tumour biopsies for research to be performed prior to commencing study treatment, and from post-progression biopsies (optional).

Refer to the Biospecimen Sampling Manual for procedures.

6.2.7 Ascites/ Pleural Fluid Collection

Currently Melbourne sites only (pending future funding for non-Melbourne sites): Participants may require ascites or pleural fluid drainage for symptom management as part of standard of care. Optional consent will be sought for the collection of ascites/ pleural fluid for translational studies for participants enrolled in some hospital sites.

6.3 Follow-up After Treatment

Follow-up after treatment and also for those participants who have progressed or stopped treatment due to any other reason, continues according to the protocol until the end of the study. This is estimated to be at least 38 months after randomisation of the last participant. Participants who are unable to attend the clinic may be followed by telephone calls to their home or other medical staff.

7 OUTCOMES, ENDPOINTS AND OTHER MEASUREMENTS

7.1 Progression Free Survival

Progression free survival (PFS) is defined as the interval from the date of randomisation until the date of first evidence of disease progression or the date of death, whichever occurs first, or otherwise the date the participant is last known to be alive and progression-free (i.e., last clinical assessment). If a participant receives other anti-cancer therapy, they will be considered to have clinical progression. If a participant receives other anti-cancer therapy for reasons not related to disease progression, consideration will be given to censoring the participant at the date she was last seen for tumour assessment before starting the new chemotherapy.

Disease progression is defined according to RECIST 1.1 (Appendix 9). The primary endpoint ofthis trial is the PFS rate at 36 weeks (9 months) from randomisation. PFS according to RECIST 1.1 or GCIG CA125 criteria and PFS according to iRECIST (Appendices 9, 10 and 11) are secondary endpoints for this trial.

7.2 Objective Response Rate

Objective response rate (ORR) is defined as the proportion of participants experiencing a complete response (CR) or partial response (PR) by RECIST 1.1 or GCIG CA125 criteria (Appendices 9 and 10). ORR by RECIST 1.1 and GCIG CA125 criteria is a secondary endpoint.

Objective tumour response rate (OTRR) in each arm is defined as the proportion of participants with an objective tumour response (OTR = CR + PR) divided by the total number of participants with measurable disease at baseline in that cohort. OTRR according to iRECIST is a secondary endpoint for this trial.

7.3 Frequency and Severity of Adverse Events

See Section 8.1 for the definition of an adverse event (AE), and reporting of Serious Adverse Events (SAEs).

The NCI Common Terminology Criteria for Adverse Events version 4 (NCI CTCAE v5.0) will be used to classify and grade the intensity of adverse events after each treatment cycle.

7.4 Health Related Quality of Life

Aspects of health related quality of life (QOL) will be assessed with the EORTC QLQ-C30, OV28 and Measure of Ovarian Symptoms and Treatment (MOST) recent status questionnaires.(24-26) The QOL Core Questionnaire 30 (QLQ-C30) was designed by the European Organization for Research and Treatment of Cancer (EORTC) Quality of Life Group in 1991. It initially comprised of 36 items, but has undergone multiple revisions with improvements in the scales which has resulted in the present 30 item measure (EORTC QLQ-C30 version 3), and is now the most widely used measure in oncology. The ovarian cancer module OV28 addresses additional important symptoms and concerns not included in QLQ-C30, and like all EORTC modules, is designed to be used in conjunction with the QLQ-C30.

The MOST is an ovarian cancer symptom benefit instrument comprising 35 individual items which provide comprehensive coverage of all the symptoms and aspects of QOL identified as those most noticed by patients from the Gynecologic Cancer Intergroup Symptom Benefit Study.(26) The form asks participants to rate the troublesomeness of symptoms and concerns “on average during the last 4 weeks”.

The aim of the health-related QOL component of this study is to describe the scores for global QOL, fatigue, abdominal/gastrointestinal symptoms of participants on study treatment and until disease progression. The primary QOL endpoint is the mean change in QLQ-C30 global QOL score at 36 weeks after randomisation in each arm. This is consistent with the primary trial outcome of 36 week PFS rate.

Secondary PRO objectives are to determine in each arm:

- Mean QLQ-C30 global scores at 4, 8 and 12 weeks after randomisation

- Mean individual item scores for Fatigue, Nausea and Vomiting from QLQ-C30 assessed every 4 weeks
- Mean scores for abdominal/gastrointestinal symptoms from OV28 and MOST at progression and prior to progression
- QLQ-C30 global scores at progression
- Time to increase in OV28 abdominal/gastrointestinal symptom subscale and in abdominal/gastrointestinal symptoms items in MOST in participants coming off study for progression. This will be an exploratory endpoint for the Phase 3 trial.

7.5 Time to Starting First Subsequent Therapy or Death

The time to starting first subsequent therapy (TSFT) or death is defined as the interval from the date of randomisation until the date a participant starts subsequent chemotherapy after stopping study treatment, or death, whichever occurs first.

7.6 Tertiary/correlative Measures

Translational research studies will include/comprise identifying immune or genomic biomarkers that are prognostic and/or predictive of response to treatment, safety and resistance to study treatment (associations of biomarkers with clinical outcomes). Studies will be outlined in a separate translational protocol to be submitted to local ethics committees and may include, but are not limited to:

- Investigating cellular biomarkers of response to treatments including a Cx-Upregulation (CUP) test that may identify responders to LDCy
- Assessment of germline and tumour DNA for DNA repair gene mutations and BRCA1 and RAD51C methylation
- Investigating changes in circulating tumour DNA that may clarify response or resistance
- Analysis of post-progression tissue biopsies (where available) to investigate mechanisms of resistance to study treatment including the generation and analysis of long-lived cell lines
- Analysis of soluble biomarkers of response to treatments in blood
- Identification of genomic signatures in baseline archival tumour which may clarify response or resistance
- Assessment of cytokine signature in baseline archival tumour which may enhance understanding of response or resistance
- Assessment of genomic signatures at baseline and post-treatment in blood
- Assessment of PD-L1 Immunohistochemistry as an predictive biomarker assay
- Assessment of change in ctDNA level from baseline could be a valid surrogate marker for treatment benefit

Since the identification of new biomarkers correlating with disease activity and the efficacy or safety of treatment is rapidly evolving, the definitive list of biomarkers remains to be determined.

8 SAFETY REPORTING

8.1 Definitions

An ADVERSE EVENT (AE) is any untoward medical occurrence in a patient or clinical investigational participant administered a pharmaceutical product and which does not necessarily have a causal relationship with this treatment. An AE can therefore be any unfavourable or unintended sign (including an abnormal laboratory finding), symptom, or disease temporally associated with the use of a medicinal investigational product, whether or not considered related to the medicinal product (see below).

AEs include the following:

- All suspected adverse drug or device reactions
- All reactions from drug or device – overdose, abuse, withdrawal, sensitivity, toxicity or failure of expected pharmacological action (if appropriate)
- Apparently unrelated illnesses
- Worsening (severity, frequency) of pre-existing illnesses or symptoms
- Injury or accidents
- Abnormalities in physiological testing or physical examination that require clinical intervention or further investigation (beyond ordering a repeat examination)
- Laboratory abnormalities that require clinical intervention or further investigation (beyond ordering a laboratory test). Any untoward event that occurs after the protocol-specified reporting period which the Investigator believes may be related to the drug or device.

A SERIOUS ADVERSE EVENT (SAE) is any untoward medical occurrence that at any dose:

- results in death,
- is life-threatening (i.e. the participant is at risk of death at the time of the event),
- requires inpatient hospitalisation or prolongation of existing hospitalisation,
- results in persistent or significant disability or incapacity,
- is a congenital anomaly/birth defect,
- other important medical events which, in the opinion of the investigator, are likely to become serious if untreated, or as defined in the protocol

NOTES:

(i) The term “life-threatening” in the definition of “serious” refers to an event in which the participant was at risk of death at the time of the event; it does not refer to an event which hypothetically might have caused death if it were more severe.

(ii) Important medical events which may not be immediately life-threatening or result in death or hospitalization but which may jeopardize the participant or may require intervention to prevent one of the listed outcomes in the definition above should also be considered serious.

In Australia, the following definitions are used for reporting of safety events;

- A SIGNIFICANT SAFETY ISSUE (SSI) is defined as a safety issue that could adversely affect the safety of participants or materially impact on the continued ethical acceptability or conduct of the trial. These events may be in addition to the current SAE/SADR/SUSAR reports and generally have a consequence related to participant safety within the current study protocol, which thus requires some type of amendment.
- An URGENT SAFETY MEASURE (USM) is one type of significant safety issue where sponsors or trial investigators act immediately to protect participants from an immediate hazard to their health and safety. USMs are often instigated before the TGA and HREC are notified. In these cases, it is strongly recommended that the sponsor contact the TGA within 24 hours of the measure being taken.

Examples include:

- a serious adverse event that could be associated with the trial procedures and that requires modification of the conduct of the trial;
- a hazard to the participant population, such as lack of efficacy of an IMP used for the treatment of a life-threatening disease;
- a major safety finding from a newly completed animal study (such as carcinogenicity);
- a temporary halt/termination of a trial for safety reasons;

- recommendations of the IDSMC, where relevant for the safety of participants, such as an increase in frequency or severity of an expected adverse reaction;
- single case events (e.g. toxic epidermal necrolysis, agranulocytosis, hepatic failure) that lead to an urgent safety measure).

SSIs or USMs do not necessarily meet all criteria to be considered an SAE. For purpose of safety reporting, these events are to be reported as SAE with a note that this concerns an SSI or USM.

A SUSPECTED UNEXPECTED SERIOUS ADVERSE REACTION (SUSAR) is an SAE that is related to the drug or device and is unexpected, i.e. not listed in the investigator brochure or approved Product Information; or is not listed at the specificity or severity that has been observed; or is not consistent with the risk information described in the Participant Information Sheet and Informed Consent Form or elsewhere in the protocol. An event is causally related if there is a reasonable possibility that the drug [intervention] caused the AE, i.e. there is evidence to suggest a causal relationship between the drug and the event.

For the purposes of this study, the following adverse events are not reported to the responsible coordinating centre as SAEs:

- Hospitalisations related to management of the disease under study
- Deaths related to disease under study
- Elective hospitalisations to facilitate the administration of treatment, e.g. Porta-Cath insertion
- Elective hospitalisations for other procedures, e.g. screening colonoscopy, stent change, cardiac catheter, etc.

ADVERSE EVENT OF SPECIAL INTEREST (AESI)

An adverse event of special interest (AESI) is one of scientific and medical interest specific to understanding of the Investigational Product and may require close monitoring and rapid communication by the PI to the sponsor. An AESI may be serious or non-serious. The rapid reporting of AESIs allows ongoing surveillance of these events in order to characterise and understand them in association with the use of this investigational product.

8.2 Adverse Events of Special Interest

8.2.1 AESIs for Durvalumab

AESIs for durvalumab include but are not limited to events with a potential inflammatory or immune-mediated mechanism and which may require more frequent monitoring and/or interventions such as steroids, immunosuppressants and/or hormone replacement therapy. These AESIs are being closely monitored in clinical studies with durvalumab monotherapy and combination therapy.

AESIs observed with durvalumab include:

- Diarrhea / Colitis and intestinal perforation
- Pneumonitis / ILD
- Hepatitis / transaminase increases
- Endocrinopathies (i.e. events of hypophysitis/hypopituitarism, adrenal insufficiency, hyper- and hypothyroidism and type I diabetes mellitus)
- Rash / Dermatitis
- Nephritis / Blood creatinine increases
- Pancreatitis / serum lipase and amylase increases
- Myocarditis
- Myositis / Polymyositis
- Neuropathy / neuromuscular toxicity (e.g. Guillain-Barré, and myasthenia gravis)
- Other inflammatory responses that are rare / less frequent with a potential immune-mediated aetiology include, but are not limited to, pericarditis, sarcoidosis, uveitis and other

events involving the eye, skin, haematological and rheumatological events.

It is important to remember that there are other rare autoimmune related events that have been documented with similar class agents that have not been seen with durvalumab, but should still be considered in the event of an adverse event that cannot be otherwise accounted for by infection or other cause. Further information on these risks (e.g. presenting symptoms) can be found in the current version of the durvalumab Investigator Brochure.

An immune-related adverse event (irAE) is defined as an adverse event that is associated with drug exposure and is consistent with an immune-mediated mechanism of action and where there is no clear alternate aetiology. Serologic, immunologic, and histologic (biopsy) data, as appropriate, should be used to support an irAE diagnosis. Appropriate efforts should be made to rule out neoplastic, infectious, metabolic, toxin, or other etiologic causes of the irAE.

If the Investigator has any questions about an adverse event (AE) being an irAE, the Investigator should promptly contact the Study Chair.

8.2.2 AESIs for Olaparib

AESIs for olaparib are the Important Potential Risks of new primary malignancy (other than MDS/AML) and pneumonitis, and the Important Identified Risk of MDS/AML. ANY event of MDS/AML, new primary malignancy, or pneumonitis should be reported to the sponsor whether it is considered a non-serious AE [e.g. non-melanoma skin cancer] or SAE, and regardless of investigator's assessment of causality or knowledge of the treatment arm.

8.3 Recording Adverse Events

AEs will be recorded from the first dose of study treatment until 90 days after cessation of study treatment. The National Cancer Institute Common Terminology Criteria for Adverse Events version 4 (NCI CTCAE v5.0) will be used to classify and grade the intensity of AEs after each treatment cycle. The worst grade will be recorded for each event.

The following information will be recorded for each SAE**:**

- Event description including classification according to NCI CTCAE
- Reason for classification as an SAE (death, hospitalisation etc.)
- Severity / worst grade
- Attribution to study intervention
- Expectedness
- Action taken with study intervention (including rechallenge, if done)
- Outcome of SAE, including end date if recovered.

8.4 Pregnancy

In the event of a pregnancy occurring during the course of a study, the participant must be withdrawn from study drug immediately. Pregnancies occurring up to 6 months after the completion of the study drug must also be reported to the investigator. The investigator should counsel the participant; discuss the risks of continuing with the pregnancy and the possible effects on the foetus.

The NHMRC CTC must be notified within 24 hours and the participant followed during the entire course of the pregnancy and postpartum period. Parental and neonatal outcomes must be recorded even if they are completely normal.

8.5 Overdose

8.5.1 Olaparib

Use of study medication in doses in excess of that specified in the protocol is considered to be an overdose. There is limited clinical experience with overdose of olaparib. In the event of overdose, appropriate supportive management should be instigated. There is no known antidote for olaparib

and treatment of adverse effects associated with its use should be for the underlying adverse symptoms. The Maximum Tolerated Dose is 300mg bid (tablet).

Adverse reactions associated with overdose should be treated symptomatically and should be managed appropriately. An overdose with associated AEs is recorded as the AE diagnosis/symptoms on the relevant AE modules in the eCRF.

8.5.2 Cyclophosphamide

The most serious consequences of overdose are myelosuppression (particularly granulocytopenia) and haemorrhagic cystitis. Bleeding, possibly severe, may occur from the bladder and gastrointestinal tract.

Profound myelosuppression may require such measures as blood transfusions, antibiotic therapy, colony stimulating factors (G-CSF, GM-CSF) and reverse barrier nursing. Consider administration of activated charcoal in the event of a potentially toxic ingestion. Activated charcoal is most effective when administered within one hour of ingestion. In participants who are not fully conscious or have impaired gag reflex, consideration should be given to administering activated charcoal via nasogastric tube once the airway is protected. In all cases, forced alkaline diuresis with copious fluid intake, using diuretics if necessary, should be employed.

Participants should be observed for signs of fluid overload and monitored for electrolyte disturbances and renal failure. In rare cases, haemodialysis may be employed to treat electrolyte disturbances and renal failure when traditional measures are ineffective. Mesna may be considered for protection of the urinary system, particularly in cases of haemorrhagic cystitis.

8.6 Reporting of Serious Adverse Events (including SUSARs)

The investigator is responsible for reporting all SAEs (including SUSARs) occurring during the study to the NHMRC CTC within 24 hours of investigational site staff becoming aware of the event according to the procedure documented in the Study Manual. The reporting period for SAEs is the period immediately following the first dose of study treatment until 90 days after the last dose of study treatment or until the initiation of alternative anticancer therapy.

The NHMRC CTC will be responsible for providing reports to the Lead HREC. The NHMRC CTC will provide SUSAR reports and SAE line listings to Investigators for submission to Human Research Ethics Committees (HRECs) as required. The investigator must notify the local HREC as required.

The NHMRC CTC will submit ‘reportable safety events’ to the TGA in Australia in time to comply with the requisite specified regulatory time windows (usually 7 days for fatal/life threatening events with an 8 day follow-up report, and 15 days for other events) .

When an urgent safety measure occurs, where possible, the CTC will notify the TGA within 24 hours and, no later than 72 hours of the measure being taken. SSIs related to USMs should be reported to the TGA by the Sponsor within 72 hours. The CTC will report all other SSIs to be reported to the TGA within 15 calendar days.

9 CENTRAL REVIEW

9.1 Central Tissue Collection

The availability of formalin-fixed paraffin-embedded (FFPE) tumour tissue is required and will be collected for translational studies from all participants. The availability of FFPE tissue will be confirmed during screening and tissue requested later by the central genomic laboratory. Tissue from all participants will be couriered from sites every six months to the central genomics laboratory for central review and storage for subsequent tertiary correlative translational studies. Refer to the Biospecimen Sampling Manual for the details regarding central tissue collection.

9.2 Central Blood Collection

Blood samples will be collected from all participants (required) for translational research at up to 26

time-points: baseline, on days 1 and 15 of cycles 1-3 (priming phase), on day 1 of cycles 4-15 (early consolidation phase), on day 1 of every 4th cycle for cycles 16-39 (late consolidation phase) and at progression.

The research samples will be shipped in real-time to the central immunology laboratory for processing, storage and research. Refer to the Biospecimen Sampling Manual for procedures.

9.3 Central Ascites/ Pleural Fluid Collection

Melbourne sites only (pending future funding for non-Melbourne sites): where paracentesis or pleurocentesis is clinically indicated, ascites or pleural fluid (surplus to diagnostic requirements) will be collected for participants who have consented to this option. Refer to the Biospecimen Sampling Manual for procedures.

10 CENTRAL STORAGE OF BIOSPECIMENS

Tumour, blood and fluid samples will be stored in a secure central laboratory in Australia. All samples will be stored with coded identification only. No identifying information will be held at the central laboratory. Refer to the Biospecimen Sampling Manual for further information.

Sample collection and storage are overseen by the ethics and regulatory approval of the SOLACE2 study. All proposed translational work using the biospecimens will be reviewed by the SOLACE2 Trial Management Committee (TMC), ANZGOG and obtain approval from a Human Research Ethics Committee.

11 STUDY TREATMENT SUPPLY AND ACCOUNTABILITY

The following investigational products will be used as study treatments:

INTERVENTION: Durvalumab with and without immunological “priming” low dose cyclophosphamide (LDCy)

CONTROL: Olaparib

11.1 Study Treatment and Supply - Durvalumab

Durvalumab (MEDI 4736) is formulated at 50 mg/mL in 26 mM histidine/histidine-HCl, 275 mM trehalose dihydrate, 0.02% (w/v) polysorbate 80, pH 6.0.

Durvalumab is supplied as a vialed liquid solution in clear 10R glass vials closed with an elastomeric stopper and a flip-off cap overseal. Each vial contains 500 mg (nominal) of durvalumab at a concentration of 50 mg/mL (500 mg/vial). The solution will be diluted with 0.9% (w/v) saline for IV infusion.

The final concentration of durvalumab in the IV bag must be between 1 and 15mg/mL.

Total time from needle puncture of durvalumab vial to start of administration should not exceed 24 hours at 2-8°C or 4 hours at room temperature.

If a participant’s weight is ≤ 30kg at baseline or during study, this participant should receive weight-based dosing with durvalumab 20 mg/kg Q4W. If the same participant’s weight improves to >30 kg, fixed dosing of durvalumab 1500mg is recommended.

Unopened vials of liquid durvalumab must be stored at 2°C to 8°C (36°F to 46°F). Durvalumab should be kept in secondary packaging until use to avoid prolonged exposure to light.

Durvalumab must be used within the individually assigned expiry date on the label.

Durvalumab will be provided by Astra Zeneca via the central pharmacy. Participating institutions will be provided with a start-up supply of study medication once the institution has been activated to commence accrual by the NHMRC CTC. Please see the Pharmacy Manual for further details.

11.2 Study Treatment and Supply – Olaparib

Olaparib will be supplied as 150 mg and 100 mg tablets in bottles containing 32 tablets. The bottles are constructed of high density polyethylene (HDPE). The 150 mg tablets are to be used for the standard dose (300 mg BD). With a 32 tablet count per bottle, each participant will need 4 bottles for 1 month's supply. The 100 mg tablets are available to manage dose reductions (if required).

Storage conditions for olaparib are 'store below 30°C'.

Olaparib will be provided by Astra Zeneca via the central pharmacy. Participating institutions will be provided with a start-up supply of study medication once the institution has been activated to commence accrual by the NHMRC CTC. Please see the Pharmacy Manual for further details.

11.3 Study Treatment and Supply - Cyclophosphamide (LDCy)

Cyclophosphamide (LDCy) will be accessed in Australia via the Pharmaceutical Benefits Scheme (PBS) according to usual hospital practice.

11.4 Drug Accountability

The Pharmacy Department at participating institutions will maintain a record of drugs dispensed for each participant and subsequent returns. The Pharmacy will also maintain a record of drug receipt for durvalumab and olaparib and drug destruction for olaparib.

Drug dispensing records will be maintained for cyclophosphamide (LDCy) accessed via PBS stock. Participant medical records must record the drugs and doses and dates prescribed.

Participants will be asked to return unused drug of olaparib and cyclophosphamide and empty drug containers at each return visit.

11.5 Background treatment

Adequate prophylactic and/or supportive care will be supplied according to usual hospital practice.

12 STATISTICAL CONSIDERATIONS

12.1 Sample Size

The choice of PFS rate at the 36-week (9-month) time-point from randomization is considered optimal for providing the best possible data to address the primary hypothesis. Based on Rustin et al (MRC OV05/EORTC 55955, Lancet 2010) from the first rise in CA125, the median time to second chemotherapy was 5.6 months for all patients with treatment delayed to the time of symptoms. It is reasonable to assume that for the subset of patients with BRCA mutant and other HRD HGSOC, the median time to second chemotherapy would be delayed with study treatment, to around 9 months from first rising CA125.

The sample size calculation is based on results from ARIEL2 and other emerging data. The participant population will be equivalent to the BRCA mutant and loss of heterozygosity (LOH) "high" group of ARIEL2. It is anticipated that BRCA mutant and LOH "high" patients will constitute 33% and 67% respectively of the recruited participant population in SOLACE2 as estimated from the ARIEL2 trial. The PFS rates at 36 weeks for the BRCA mutant and LOH "high" group of ARIEL2 are estimated to be 70% and 35% respectively. Therefore, the average PFS rate at 36 weeks, weighted according to study population incidence of the BRCA mutant and LOH "high" group is estimated to be 47%.

Assuming olaparib monotherapy (arm C) will have a PFS rate at 36 weeks of 47%, we expect that combination therapy (arm A and/or arm B) will result in 20% improvement over olaparib monotherapy. The choice of 20% is also considered to be clinically meaningful as we expect that this PFS improvement will offset any potential increased toxicity from combination therapy.

Using Fleming's single stage design, with 95% confidence and 80% power, 38 participants per arm will be required in order to exclude at 36 weeks an uninteresting PFS rate of 47% in favour of a more clinically meaningful PFS rate of 67%. Additionally as there are 3 groups a total of 114 participants will also allow (using the selection design of Simon, Wittes, and Ellenberg) a minimum of 15% difference from the best performing regimen to be detected with a probability >0.80. The best

performing regimen will then be selected to inform a Phase III study.

In each of the combination arms (arm A and arm B), 38 participants will be recruited. The contemporary control arm (arm C) also with 38 participants is important to ensure that the assumption of a PFS rate at 36 weeks for olaparib monotherapy is approximately 47%.

The total sample size for the study is 114 participants and participants found to be ineligible after randomisation will be replaced.

12.2 Event Monitoring

Serious adverse events will be monitored by the trial management committee on an ongoing basis to ensure that the rate of these events remains acceptable for the study. No interim analysis is planned for this study.

12.3 Statistical Analysis

Assessments of efficacy data will be based on the intention-to-treat (ITT) principle. Analysis of safety endpoints (i.e., toxicity) will be according to treatment received, including only participants who started at least 1 cycle of treatment. All p-values will be two tailed. A nominal significance level of 0.05 will be applied. The anticipated final analysis will be performed after 12 months of follow-up from randomisation, or if the amount of actual information available (at a 36-week PFS comparison) is greater than 80%.

12.3.1 Primary Endpoint

Progression-free survival (PFS) will be analysed using time-to-event methods. The PFS rate at 36 weeks will be calculated using Kaplan-Meier estimates, with corresponding 95% confidence intervals (CIs). Kaplan-Meier curves will be constructed for graphical display and the log-rank test used to compare the PFS distribution between treatment arms. Median PFS (with 95% CIs) will be calculated for each treatment arm. The Cox proportional hazards model will be used to obtain hazards ratios for the pairwise treatment comparisons and the associated 95% CIs.

12.3.2 Secondary Endpoints

Proportions of participants achieving objective responses will be calculated with appropriate 95% confidence intervals. Proportions of participants suffering grade 3 or worst toxicity will be reported by treatment arm. Comparisons of proportions between treatment arms will be performed using chi-squared or exact tests.

The primary QoL endpoint change in QLQ-C30 global score from baseline to 36 weeks will be analysed using regression methods including adjustment for baseline variables and other appropriate statistical methods. Amongst progressors the time to clinically significant increase in abdominal/gastrointestinal symptom scores will be analysed using the Kaplan-Meier method and described using the median with 95% CI. Abdominal/gastrointestinal scores at progression and prior to progression will be compared using the paired t-test.

Time to starting first subsequent therapy or death will be analysed using time-to-event methods, similarly as for PFS above, and characterised in terms of the median (with 95% CI).

13 STUDY ORGANISATION and COMMITTEES

13.1 Study coordination

The study is a locally developed and led investigator initiated collaborative group study. Coordination, monitoring, data acquisition and management and statistical analysis will be performed by the NHMRC CTC. Management of the translational component will be performed by ANZGOG.

13.2 Trial Management Committee

The Trial Management Committee (TMC) will oversee study planning, monitoring, progress, review

of information from related research, and implementation of recommendations from other study committees and external bodies (e.g. ethics committees).

The TMC will consider whether to continue the study as planned, modify, or stop it, based on real-time review of safety data and other information.

13.3 Independent Safety and Data Monitoring Committee

There will be regular monitoring of the study for safety by an Independent Safety Data Monitoring Committee (ISDMC) who will review the safety profile of the participants in the trial and make recommendations to the TMC. The ISDMC will be appointed by the TMC and will consist of a minimum of three members, comprising of at least one biostatistician/clinical epidemiologist and two oncologists. The ISDMC will also review the trial progress in light of any emerging evidence which may impact the study.

14 ADMINISTRATIVE ASPECTS

14.1 Ethics and regulatory compliance

In Australia, the study will be conducted according to the ICH Guideline for Good Clinical Practice Integrated Addendum to ICH E6 (R1): Guideline for Good Clinical Practice ICH E6(R2) dated 9 November 2016 annotated with TGA (Therapeutic Goods Administration) comments and in compliance with applicable laws and regulations. The study will be performed in accordance with the National Statement on Ethical Conduct in Human Research 2007 (updated 2018, and as amended from time to time), the Australian Code for the Responsible Conduct of Research (2018, and as amended from time to time), and the principles laid down by the World Medical Association in the Declaration of Helsinki 2013.

To this end, no participant will be recruited to the study until all the necessary approvals have been obtained and the participant has provided written informed consent. Further, the investigator shall comply with the protocol, except when a protocol deviation is required to eliminate immediate hazard to a participant. In this circumstance the NHMRC CTC, principal investigator and HREC must be advised immediately.

14.2 Confidentiality

The study will be conducted in accordance with applicable Privacy Acts and Regulations. All data generated in this study will remain confidential. All information will be stored securely at the NHMRC CTC, University of Sydney and will only be available to people directly involved with the study.

14.3 Protocol deviations and amendments

A protocol deviation is any non-compliance with the clinical trial protocol, GCP, or other ethical or regulatory requirement. The non-compliance may be either on the part of the participant, the investigator, or the study staff. As a result of deviations, corrective actions are to be developed by the Sponsor and implemented promptly. All deviations must be addressed in study source documents, reported to the CTC. Protocol deviations should be sent to the Lead HREC and RGO as per their local guidelines.

Changes and amendments to the protocol can only be made by the TMC. Approval of amendments by the Institutional HREC is required prior to their implementation. In some instances, an amendment may require a change to a consent form. The Investigator must receive approval/advice of the revised consent form prior to implementation of the change. In addition, changes to the data collected, if required, will be incorporated in the amendment.

The investigator should not implement any changes to, or deviations from, the protocol except where necessary to eliminate immediate hazard(s) to trial participant(s).

In response to unforeseen circumstances (e.g. natural disasters, pandemics, etc.), the study Investigators may direct an immediate change to the protocol with the primary aim of continuing health care and ensuring the participant's best interests at all times. Changes will be communicated

with the relevant parties e.g. the lead HREC/RGOs and regulatory authorities if appropriate, and participants in due course.

14.4 Data Handling and Record Keeping

All trial data required for the monitoring and analysis of the study will be recorded on the (e)CRFs provided. All required data entry fields must be completed. Data corrections will be done according to the instructions provided. The investigator will be asked to confirm the accuracy of completed CRFs by signing key CRFs as indicated.

Source documents pertaining to the trial must be maintained by investigational sites. Source documents may include a participant's medical records, hospital charts, clinic charts, the investigator's participant study files, as well as the results of diagnostic tests such as X-rays, laboratory tests, and electrocardiograms. The investigator's copy of the case report forms serves as part of the investigator's record of a participant's study-related data.

The following information should be entered into the participant's medical record:

a. The participant’s protocol identification.

b. The date that the participant entered the study, and participant number.

c. A statement that informed consent was obtained (including the date)

d. Relevant medical history

e. Dates of all participant visits and results of key trial parameters.

f. Occurrence and status of any SAEs and AESIs

g. The date the participant exited the study, and a notation as to whether the participant completed the study or reason for discontinuation.

All study-related documentation at ANZ sites will be maintained for 15 years following completion of the study.

14.5 Study Monitoring

Data from this study will be monitored by Clinical Trials Program staff from the NHMRC CTC or their delegates in accordance with a separate study monitoring plan. Monitoring will include centralised review of CRFs and other study documents for protocol compliance, data accuracy and completeness. Monitoring may include monitoring visits to investigational sites during the study for source data verification, review of the investigator’s site file and drug handling records. The NHMRC CTC will be given direct access to source documents, CRFs and other study-related documents. By signing the informed consent form, the participant gives authorised NHMRC CTC staff direct access to their medical records and the study data.

14.6 Audit and Inspection

This study may be participant to audit or inspection by representatives of ANZGOG, Astra Zeneca, the CTC or representatives of local or international regulatory bodies (e.g. Therapeutic Goods Administration (TGA)).

14.7 Clinical Study Report

A Clinical Study Report which summarises and interprets all the pertinent study data collected will be issued which may form the basis of a manuscript intended for publication. The Clinical Study Report or summary thereof will be provided to Astra Zeneca.

14.8 Publication Policy

The Trial Management Committee will appoint a Writing Committee to draft manuscript(s) based on the trial data. Manuscript(s) will be submitted to peer-reviewed journal(s). The Writing Committee will develop a publication plan, including authorship, target journals and expected dates of publication. All publications must receive prior written approval from the TMC prior to submission.

15 PROTOCOL AMENDMENTS

Amendment no.	Date	Summary of change
2.0	21 Jun 2018	Number of durvalumab cycles changed from 12 to 36
2.1	28 March 2019	Update of CTCAE from version 4 to version 5<br>Improved definition of Platinum-Free Interval (PFI) days for Stratification factor<br>Update of Olaparib IB from version 15 to 16<br>Update of Durvalumab IB from version 12 to 14<br>MOST Quality of Life questionnaire version correction (v1, not v2)<br>Participants over 70 years of age now included in the study<br>Specification that all TR bloods are collected at site<br>Correction of total TR blood collection time points from 25 to 26.<br>Removal of reference to reimbursement of costs to participants<br>Inclusion of Ascites and/or pleural fluid collection<br>Specification of TR blood collection protocol in instances of delayed treatment<br>Clarification of TR blood collection timing<br>Correction of term *Biologica*l Sampling Manual to *Biospecimen* Sampling Manual<br>Clarification of Upper Limit of Normal (ULN) CA125 definition (35 units/mL)<br>Clarification to text surrounding the destruction of patient data.

3.0	13 November 2019	Clarified cyclophosphamide dosing<br>Amended inclusion criteria 2 to allow patients without prior adequate debulking surgery (if not appropriate) and prior hormonal therapy as part of first-line treatment<br>Amended inclusion criteria 3 to allow patients with normalised CA125 readings < ULN by the end of first-line chemotherapy (previously by end of cycle 4 of first-line chemotherapy)<br>Amended inclusion criteria 4 by removing "First" CA125 GCIG progression and added that in the absence of RECIST measurable disease, CA125 GCIG progression after first-line treatment occurring <u>4 months or more</u> is permitted<br>Amended inclusion criteria 5 to allow patients with normal CA125, but have RECIST measurable disease; if no measurable disease, they must have CA125 readings twice the ULN<br>Removed inclusion criteria of "No symptoms of cancer progression" as it contradicts with inclusion criteria of "ECOG 0-1"<br>Amended exclusion criteria 11 to "no history of other active malignancy"<br>Clarified in exclusion criteria 18 that patients with documented serologically positive hepatitis B or C will be excluded<br>Extended toxicity break from 14 to 28 days<br>Additional details regarding randomisation methodology<br>Clarified wording on the two separate translational research blood collections prior to starting study treatment<br>Amended patient diary to dosing sheet<br>Administrative changes
4.0	24 November 2020	Addition of ANZCTR<br>Amended "registration" to "randomisation";<br>Amended screening window to be within 28 days prior to randomisation (previously within 21 days);<br>Clarified that oral cyclophosphamide 50mg is to be taken daily on days 1-5 <u>consecutively</u> under "*Study Treatments"* in *Synopsis and Schema* section and under "*Table 2. Study treatments"* in Section 5.1.1 *Study Treatment;*<br>Clarified Section 4.2, Inclusion Criteria #5 – participants can have RECIST measurable or non-measurable disease, or no disease, but all participants must have raised CA125 readings twice the ULN on two occasions;<br>Removed "+/- 5 minute" window for Durvalumab infusion in Section 5.1.4 *Durvalumab Administration*<br>Amended "*Table 4. Management of olaparib and cyclophosphamide for haematological AEs"* in Section 5.2.2.1.1 *Haematological AEs* to allow for up to 28 days delay for haemoglobin to recover;<br>Clarified Section 5.4.2 *Prohibited Concomitant Medications* – probiotics are not considered herbal medications;

		Clarified Section 5.4.4 *Concomitant Medication Reporting* – concomitant medications only need to be recorded in the eCRF if they were administered within 30 days prior to an SAE;<br><br>Clarified Section 5.7 *Study Treatment Discontinuation* – participants continuing study treatment beyond disease progression should continue to follow the on-treatment schedule as per protocol and all CT-CAP scans should continue according to original schedule;<br><br>Updated Section 6.1 *Schedule of Assessments* to:<br>• clarify that Day 15 TR bloods must be taken within 3 days prior to study treatment dosing;<br>• clarify Footnote #5 – at Screening, two ECG assessments within a 24-hour period is required and any subsequent ECG assessments only if clinically indicated;<br>• clarify Footnote #8 – 12-weekly Follow-Up should be calculated from EOT date;<br>• clarify Footnote #11 – scans may be performed within a +/-7 day window period, and that confirmatory scans within 4-8 weeks are only required for participants continuing Durvalumab beyond disease progression;<br>• clarify Footnote #13 – if participant discontinues treatment for reasons other than disease progression, Clinic assessment, Haematology, Biochemistry & TFTs can continue at Investigator's discretion. All other assessments should continue as per protocol until disease progression, the participant has commenced another anti-cancer therapy or participant has withdrawn consent;<br>• add Footnote #15 – repeat CA125 test required where possible (i.e. prior to starting another anti-cancer therapy), at least one week after the CA125 test where progression was first detected;<br>• clarify which assessments are required at progression – added biochemistry, haematology and TFTs, CA125, and SAE review;<br>• clarify which assessments are required after progression – added repeat CA125 test, SAE review (until 90 days after last dose of study treatment);<br><br>Clarified "*Vital Signs*" under Section 6.2.2 *Clinical Assessment* – vital sign measurements are only required for first Durvalumab infusion only (pre-, during and post-infusion). Institutional standard of care may be followed for subsequent infusions;<br><br>Clarified "*ECGs*" under Section 6.2.2 *Clinical Assessment* – only two assessments within a 24-hour period is required at Screening;<br><br>Clarified Section 6.2.3 *Imaging* – as per Footnote #11 under *Schedule of Assessments;*<br><br>Updated Section 8.1 *Definitions* to include definitions used in reporting of safety events in Australia, and examples;<br><br>Updated Section 8.6 *Reporting of Serious Adverse Events (including SUSARs)* to include Sponsor actions for urgent safety measures;<br><br>Updated Section 14.1 *Ethics and regulatory compliance* to reference the latest guidelines;<br><br>Updated Section 14.3 *Protocol deviations and amendments* to

		include definition of a protocol deviation and response to unforeseen circumstances;<br>Updated Appendix 4 *Management of Immune-Related Adverse Events (imAEs)* in line with the current Durvalumab Toxicity Management Guidelines dated 17 November 2020;<br>Administrative changes.
5.0	06 May 2021	Updated Section 1.3 *Platinum Sensitivity* wording to be in line with the current inclusion criteria text;<br>Section 4.2 *Inclusion Criteria* number 4 clarified;<br>Section 4.3 *Exclusion Criteria*: no.15 – correction from 218 days to 28 days;<br>Section 5.2 *Dose Modifications* and Section 5.2.2.1.1 *Haematological AEs* – correction to treatment delay (14 to 28) days.<br>Updated Section 6.2.2 *Clinical Assessment* with window for CXD1 visits;<br>Updated 6.2.5 *Blood Collection* – added text to clarify timepoints for TR blood collections during treatment delay;<br>Updated Section 9.1 *Central Tissue Collection* – TR tissue request to occur “later from the central genomic laboratory” instead of “from the diagnostic pathologist at the time of enrolment”.

16 REFERENCES

1. Network CGAR. Integrated genomic analyses of ovarian carcinoma. Nature. 2011;474(7353):609.
2. Harter P, Johnson T, Berton-Rigaud D, Park S-Y, Friedlander M, Del Campo JM, et al. BRCA1/2 mutations associated with progression-free survival in ovarian cancer patients in the AGO-OVAR 16 study. Gynecologic oncology. 2016;140(3):443-9.
3. Ledermann J, Harter P, Gourley C, Friedlander M, Vergote I, Rustin G, et al. Olaparib maintenance therapy in platinum-sensitive relapsed ovarian cancer. New England Journal of Medicine. 2012;366(15):1382-92.
4. Ledermann J, Harter P, Gourley C, Friedlander M, Vergote I, Rustin G, et al. Olaparib maintenance therapy in patients with platinum-sensitive relapsed serous ovarian cancer: a preplanned retrospective analysis of outcomes by BRCA status in a randomised phase 2 trial. The lancet oncology. 2014;15(8):852-61.
5. Audeh MW, Carmichael J, Penson RT, Friedlander M, Powell B, Bell-McGuinn KM, et al. Oral poly (ADP-ribose) polymerase inhibitor olaparib in patients with BRCA1 or BRCA2 mutations and recurrent ovarian cancer: a proof-of-concept trial. The Lancet. 2010;376(9737):245-51.
6. Scott CL, Swisher EM, Kaufmann SH. Poly (ADP-ribose) polymerase inhibitors: recent advances and future development. Journal of Clinical Oncology. 2015;33(12):1397-406.
7. Brahmer JR, Tykodi SS, Chow LQ, Hwu W-J, Topalian SL, Hwu P, et al. Safety and activity of anti–PD-L1 antibody in patients with advanced cancer. N Engl J Med. 2012;366(26):2455-65.
8. Hamanishi J, Mandai M, Ikeda T, Minami M, Kawaguchi A, Murayama T, et al. Safety and antitumor activity of anti–PD-1 antibody, nivolumab, in patients with platinum-resistant ovarian cancer. Journal of Clinical Oncology. 2015;33(34):4015-22.

9. Preston CC, Maurer MJ, Oberg AL, Visscher DW, Kalli KR, Hartmann LC, et al. The ratios of CD8+ T cells to CD4+ CD25+ FOXP3+ and FOXP3-T cells correlate with poor clinical outcome in human serous ovarian cancer. PloS one. 2013;8(11):e80063.

10. Eng KH, Tsuji T. Differential antigen expression profile predicts immunoreactive subset of advanced ovarian cancers. PloS one. 2014;9(11):e111586.

11. Yigit R, Figdor CG, Zusterzeel PL, Pots JM, Torensma R, Massuger LF. Cytokine analysis as a tool to understand tumour–host interaction in ovarian cancer. European Journal of Cancer. 2011;47(12):1883-9.

12. Barnett JC, Bean SM, Whitaker RS, Kondoh E, Baba T, Fujii S, et al. Ovarian cancer tumor infiltrating T-regulatory (T reg) cells are associated with a metastatic phenotype. Gynecologic oncology. 2010;116(3):556-62.

13. Curiel TJ, Coukos G, Zou L, Alvarez X, Cheng P, Mottram P, et al. Specific recruitment of regulatory T cells in ovarian carcinoma fosters immune privilege and predicts reduced survival. Nature medicine. 2004;10(9):942-9.

14. Lee JM, Ivy SP, Kohn EC. Challenges and Opportunities for Immunotherapies in Gynecologic Cancers. Oncology (Williston Park, NY). 2016;30(1):67-9.

15. Lee JM, Cimino-Mathews A, Peer CJ, Zimmer A, Lipkowitz S, Annunziata CM, et al. Safety and Clinical Activity of the Programmed Death-Ligand 1 Inhibitor Durvalumab in Combination With Poly (ADP-Ribose) Polymerase Inhibitor Olaparib or Vascular Endothelial Growth Factor Receptor 1-3 Inhibitor Cediranib in Women's Cancers: A Dose-Escalation, Phase I Study. J Clin Oncol. 2017;35(19):2193-202.

16. Lee CK, Scott CL, Lindeman GJ, Gibbs E, Badger HD, Paterson RJ, et al. Phase I study of olaparib (O), in combination with oral cyclophosphamide (C), in patients (pts) with metastatic triple negative breast cancer (TNBC) and recurrent high grade serous ovarian cancer (OVCA). American Society of Clinical Oncology; 2017.

17. Madondo MT, Quinn M, Plebanski M. Low dose cyclophosphamide: mechanisms of T cell modulation. Cancer treatment reviews. 2016;42:3-9.

18. Handolias D, Quinn M, Foo S, Mileshkin L, Grant P, Dutu G, et al. Oral cyclophosphamide in recurrent ovarian cancer. Asia-Pacific Journal of Clinical Oncology. 2016;12(1).

19. Emens LA, Middleton G. The interplay of immunotherapy and chemotherapy: harnessing potential synergies. Cancer immunology research. 2015;3(5):436-43.

20. Mirza MR, Monk BJ, Herrstedt J, Oza AM, Mahner S, Redondo A, et al. Niraparib maintenance therapy in platinum-sensitive, recurrent ovarian cancer. New England Journal of Medicine. 2016;375(22):2154-64.

21. Swisher EM, Lin KK, Oza AM, Scott CL, Giordano H, Sun J, et al. Rucaparib in relapsed, platinum-sensitive high-grade ovarian carcinoma (ARIEL2 Part 1): an international, multicentre, open-label, phase 2 trial. The Lancet Oncology. 2017;18(1):75-87.

22. Coleman RL, Oza AM, Lorusso D, Aghajanian C, Oaknin A, Dean A, et al. Rucaparib maintenance treatment for recurrent ovarian carcinoma after response to platinum therapy (ARIEL3): a randomised, double-blind, placebo-controlled, phase 3 trial. The Lancet. 2017.

23. Swisher EM, Lin KK, Oza AM, Scott CL, Giordano H, Sun J, et al. Rucaparib in relapsed, platinum-sensitive high-grade ovarian carcinoma (ARIEL2 Part 1): an international, multicentre, open-label, phase 2 trial. The Lancet Oncology. 2017;18(1):75-87.

24. Fayers PM, Aaronson NK, Bjordal K, Grønvold M, Curran D, Bottomley A. EORTC QLQ-C30 scoring manual. 2001.

25. Greimel E, Bottomley A, Cull A, Waldenstrom A-C, Arraras J, Chauvenet L, et al. An international field study of the reliability and validity of a disease-specific questionnaire module (the QLQ-OV28) in assessing the quality of life of patients with ovarian cancer. European Journal of Cancer. 2003;39(10):1402-8.

26. King MT, Stockler MR, Butow P, O'Connell R, Voysey M, Oza AM, et al. Development of the measure of ovarian symptoms and treatment concerns: aiming for optimal measurement of

patient-reported symptom benefit with chemotherapy for symptomatic ovarian cancer. International Journal of Gynecological Cancer. 2014;24(5):865-73.

27. Eisenhauer EA, Therasse P, Bogaerts J, Schwartz LH, Sargent D, Ford R, et al. New response evaluation criteria in solid tumours: revised RECIST guideline (version 1.1). European journal of cancer (Oxford, England : 1990). 2009;45(2):228-47.

28. Rustin GJS, Vergote I, Eisenhauer E, Pujade-Lauraine E, Quinn M, Thigpen T, et al. Definitions for response and progression in ovarian cancer clinical trials incorporating RECIST 1.1 and CA 125 agreed by the Gynecological Cancer Intergroup (GCIG). International journal of gynecological cancer. 2011;21(2):419-23.

29. Seymour L, Bogaerts J, Perrone A, Ford R, Schwartz LH, Mandrekar S, et al. iRECIST: guidelines for response criteria for use in trials testing immunotherapeutics. Lancet Oncol. 2017;18(3):e143-e52.

17 APPENDICES

17.1 Appendix 1 ECOG Performance Status Scales

ECOG Performance Status Scale

**Grade**	**Descriptions**
0	Normal activity. Fully active, able to carry on all pre-disease performance without restriction.
1	Symptoms, but ambulatory. Restricted in physically strenuous activity, but ambulatory and able to carry out work of a light or sedentary nature (e.g. light housework, office work).
2	In bed < 50% of the time. Ambulatory and capable of all self-care, but unable to carry out any work activities. Up and about more than 50% of waking hours.
3	In bed > 50% of the time. Capable of only limited self-care, confined to bed or chair more than 50% of waking hours.
4	100% bedridden. Completely disabled. Cannot carry on any self-care. Totally confined to bed or chair.
5	Dead.

17.2 Appendix 2 Cockroft-Gault formula

Estimation and Measurement of Glomerular Filtration Rate (GFR)

Males:

$$\text{Creatinine CL (mL/min)} = \frac{\text{Weight (kg)} \times (140 - \text{Age})}{72 \times \text{serum creatinine (mg/dL)}}$$

Females:

$$\text{Creatinine CL (mL/min)} = \frac{\text{Weight (kg)} \times (140 - \text{Age}) \times 0.85}{72 \times \text{serum creatinine (mg/dL)}}$$

To convert serum creatinine in mg/dl to µmol/l use the following formula:

Cr (µmol/l) = Cr (mg/dl) x 88.4

17.3 Appendix 3 Management of Infusion-related Reactions Associated with Durvalumab

CTCAE grade	Dose Modification	Management
Any grade		Management per institutional standard at the discretion of investigator<br>Monitor patients for signs and symptoms of infusion-related reactions (e.g. fever and/or shaking chills, flushing and/or itching, alterations in heart rate and blood pressure, dyspnea or chest discomfort, skin rashes etc.) and anaphylaxis (e.g. generalized urticaria, angioedema, wheezing, hypotension, tachycardia, etc.)
Grade 1	The infusion rate of study drug/study regimen may be decreased by 50% or temporarily interrupted until resolution of the event	Paracetamol and/or antihistamines may be administered per institutional standard at the discretion of the investigator<br>Consider premedication per institutional standard prior to subsequent doses
Grade 2	The infusion rate of study drug/study regimen may be decreased 50% or temporarily interrupted until resolution of the event (up to 4 hours).	
	Subsequent infusions may be given at 50% of the initial infusion rate	
Grade 3/4	Permanently discontinue Durvalumab.	Manage severe infusion-related reactions per institutional standards (e.g. IM adrenaline, followed by IV diphenhydramine and famotidine, and IV glucocorticoid)

17.4 Appendix 4 Management of Immune-Mediated Adverse Events (imAEs)

Based on the mechanism of action of durvalumab leading to T-cell activation and proliferation, there is the possibility of observing immune-mediated Adverse Events (imAEs) during the conduct of this study. Potential imAEs include (but are not limited to) immune mediated myocarditis, enterocolitis, dermatitis, hepatitis, and endocrinopathies. Patients are to be monitored for signs and symptoms of imAEs. In the absence of an obvious alternative cause (e.g., infection or progressive disease) signs or symptoms of enterocolitis, dermatitis, hepatitis, and endocrinopathy should be considered to be immune-related.

General and specific guidelines for the investigation, management of imAE and AESI and criteria for dose delays and discontinuation are included in the tables below.

These guidelines are provided as a recommendation to support investigators in the management of potential immune-mediated adverse events (imAEs)

Immune-mediated events can occur in nearly any organ or tissue, therefore, these guidelines may not include all the possible immune-mediated reactions. Investigators are advised to take into consideration the appropriate practice guidelines and other society guidelines (e.g., NCCN, ESMO) in the management of these events. Refer to the section of the table titled "Other Immune-Mediated Reactions" for general guidance on imAEs not noted in the "Specific Immune-Mediated Reactions".

Early identification and management of immune-mediated adverse events (imAEs) are essential to ensure safe use of the study drug. Monitor patients closely for symptoms and signs that may be clinical manifestations of underlying immune-mediated adverse events. Patients with suspected imAEs should be thoroughly evaluated to rule out any alternative aetiologies (e.g., disease progression, concomitant medications, infections). In the absence of a clear alternative etiology, all such events should be managed as if they were immune-mediated. Institute medical management promptly, including specialty consultation as appropriate. In general, withhold study drug/study regimen for severe (Grade 3) imAEs. Permanently discontinue study drug/study regimen for life-threatening (Grade 4) imAEs, recurrent severe (Grade 3) imAEs that require systemic immunosuppressive treatment, or an inability to reduce corticosteroid dose to 10 mg or less of prednisone or equivalent per day within 12 weeks of initiating corticosteroids.

More potent immunosuppressive agents such as TNF inhibitors (e.g., infliximab) should be considered for events not responding to systemic steroids. Alternative immunosuppressive agents not listed in this guideline may be considered at the discretion of the investigator based on clinical practice and relevant guidelines. With long-term steroid and other immunosuppressive use, consider need for *Pneumocystis jirovecii* pneumonia (PJP, formerly known as *Pneumocystis carinii* pneumonia) prophylaxis, gastrointestinal protection, and glucose monitoring.

Dose modifications of study drug/study regimen should be based on severity of treatment-emergent toxicities graded per NCI CTCAE version 5.0.

17.4.1 Pneumonitis/Interstitial Lung Disease Management Algorithm

Monitor participants for signs and symptoms of pneumonitis or Interstitial Lung Disease (ILD) (new onset or worsening shortness of breath or cough). Participants should be evaluated with imaging and pulmonary function tests including other diagnostic procedures as described below.

Suspected pneumonitis should be confirmed with radiographic imaging and other infectious and disease-related aetiologies excluded, and managed as described below.

Initial work-up may include clinical evaluation, monitoring of oxygenation via pulse oximetry (resting and exertion), laboratory work-up and high-resolution CT scans.

Consider Pulmonary and Infectious Diseases consults.

CTCAE grade	Dose Modification	Management
Grade 1	No modification required. Consider holding durvalumab as clinically appropriate and during diagnostic work-up for other aetiologies	Monitor and closely follow up in 2-4 days for clinical symptoms, pulse oximetry (resting and exertion) and laboratory work-up and then as clinically indicated
Grade 2	Hold durvalumab until grade 2 resolution to ≤ grade 1<br><br>- If toxicity worsens then treat as grade 3 or grade 4<br><br>- If toxicity improves to ≤ grade 1, then the decision to initiate durvalumab at next scheduled treatment date will be based upon treating physician's clinical judgement and after completion of steroid taper	Monitor symptoms daily and consider hospitalisation<br><br>Promptly start systemic steroids (e.g., prednisone 1-2mg/kg/day or IV equivalent)<br><br>Reimage as clinically indicated, consider chest CT with contrast and repeat in 3-4 weeks<br><br>If no improvement within 2 to 3 days, additional workup should be considered and prompt treatment with IV methylprednisolone 2 to 4 mg/kg/day started<br><br>If still no improvement within 2 to 3 days despite IV methylprednisolone at 2 to 4 mg/kg/day, promptly start immunosuppressive therapy such as TNF inhibitors (e.g., infliximab at 5 mg/kg IV once, may be repeated at 2 and 6 weeks after initial dose at the discretion of the treating provider). Caution: It is important to rule out sepsis and refer to infliximab label for general guidance before using infliximab.<br><br>Consider, as necessary, discussing with study physician.
Grade 3 or 4	Discontinue durvalumab permanently	Promptly initiate empiric IV methylprednisolone 1 to 4 mg/kg/day or equivalent.<br><br>Consider discussing with study physician, as needed.<br><br>Hospitalise the patient.<br><br>Supportive care (e.g., oxygen).

		If no improvement within 2 to 3 days, additional workup should be considered and prompt treatment with additional immunosuppressive therapy such as TNF inhibitors (e.g., infliximab at 5 mg/kg IV, may be repeated at 2 and 6 weeks after initial dose at the discretion of the treating provider). Caution: rule out sepsis and refer to infliximab label for general guidance before using infliximab.

17.4.2 Diarrhoea/Colitis Management Algorithm

Monitor for symptoms that may be related to diarrhoea/ enterocolitis (abdominal pain, cramping, or changes in bowel habits such as increased frequency over baseline or blood in stool) or related to bowel perforation (such as sepsis, peritoneal signs and ileus).

WHEN SYMPTOMS OR EVALUTAION INDICATE A PERFORATION IS SUSPECTED, CONSULT A SURGEON EXPERIENCED IN ABDOMINAL SURGERY IMMEDIATELY WITHOUT ANY DELAY.

PERMANENTLY DISCONTINUE STUDY DRUG FRO ANY GRADE OF INTESTINAL PERFORATION.

Participants should be thoroughly evaluated to rule out any alternative etiology (e.g. disease progression, other medications, or infections, etc.), including testing for clostridium dificile toxin, etc.

Steroids should be considered if an alternative aetiology is not determined, even for low grade events, in order to prevent potential progression to higher grade event, including intestinal perforation. Use analgesics carefully; they can mask symptoms of perforation and peritonitis.

**CTCAE grade**	**Dose Modification**	**Management**
Grade 1	No dose modification required	Close monitoring for worsening symptoms<br><br>Consider symptomatic treatment including hydration, electrolyte replacement, dietary changes, loperamide and other supportive care measures.<br><br>If symptoms persist, consider checking lactoferrin; if positive, treat as Grade 2 below. If negative and no infection, continue Grade 1 management.
Grade 2	Hold durvalumab until grade 2 resolution to ≤ grade 1<br>- If toxicity worsens then treat as grade 3 or grade 4<br>- If toxicity improves to grade ≤1,then study treatment can resume after completion of steroid taper.	Consider symptomatic treatment including hydration, electrolyte replacement, dietary changes, and loperamide and/or budesonide<br><br>Promptly start prednisone 1-2 mg/kg/day or iv equivalent<br><br>If event is not responsive within 2 to 3 days or worsens despite prednisone at 1-2 mg/kg/day PO or IV equivalent, consult a GI specialist for consideration of further workup, such

		as imaging and/or colonoscopy, to confirm colitis and rule out perforation<br>If still no improvement within 2 to 3 days despite 1-2 mg/kg IV methylprednisolone, promptly start immunosuppressant agents such as infliximab at 5 mg/kg IV may be repeated at 2 and 6 weeks after initial dose at the discretion of the treating provider.<br>Caution: it is important to rule out bowel perforation and refer to infliximab label for general guidance before using infliximab.<br>Consider, as necessary, discussing with study physician if no resolution to Grade ≤1 in 3 to 4 days.
Grade 3 or 4	<u>For Grade 3:</u><br>For patients treated with PD-L1 inhibitors, hold study drug/study regimen until resolution to Grade ≤1; study drug/study regimen can be resumed after completion of steroid taper. Permanently discontinue study drug/study regimen for Grade 3 if toxicity does not improve to Grade ≤1 within 14 days.<br>Permanently discontinue study drug for any grade of intestinal perforation in any patient treated with ICI<br><u>For Grade 4:</u><br>Permanently discontinue study drug/study regimen.	Promptly initiate empiric IV methylprednisolone 1-2 mg/kg/day or equivalent.<br>Monitor stool frequency and volume and maintain hydration.<br>Urgent GI consult and imaging and/or colonoscopy as appropriate.<br>If still no improvement within 2 days, continue steroids and promptly add further immunosuppressants (e.g., infliximab at 5 mg/kg IV, may be repeated at 2 and 6 weeks after initial dose at the discretion of the treating provider). Caution: Ensure GI consult to rule out bowel perforation and refer to infliximab label for general guidance before using infliximab.<br>**If perforation is suspected, consult a surgeon experienced in abdominal surgery immediately without any delay.**

17.4.3 Hepatitis/ Elevated LFTs Management Algorithm

Monitor and evaluate liver function test: AST, ALT, ALP and total bilirubin. Evaluate for alternative aetiologies (e.g. viral hepatitis, disease progression, concomitant medications).

INFLIXIMAB SHOULD NOT BE USED FOR MANAGEMENT OF IMMUNE RELATED HEPATITIS.

CTCAE grade	Dose Modification	Management
Grade 1	No dose modification required.<br>If it worsens, then treat as Grade 2 event.	Continue LFT monitoring per protocol
Grade 2	Hold study drug/study regimen dose until Grade 2 resolution to Grade ≤1.<br>If toxicity worsens, then treat as Grade 3 or Grade 4.<br>If toxicity improves to Grade ≤1 or baseline, resume study drug/study regimen after completion of steroid taper.<br>Permanently discontinue study drug/study regimen for any case meeting Hy's law criteria (AST and/or ALT >3 x ULN + bilirubin >2 x ULN without initial findings of cholestasis (i.e. elevated alkaline P04) and in the absence of any alternative cause[b]	Regular and frequent checking of LFTs (e.g., every 1 to 2 days) until LFT elevations improve or resolve.<br>If no resolution to Grade ≤1 in 1 to 2 days, consider discussing with study physician, as needed.<br>If event is persistent (>2 to 3 days) or worsens, promptly start prednisone 1-2 mg/kg/day PO or IV equivalent.
Grade 3	For elevations in transaminases ≤8 × ULN, or elevations in bilirubin ≤5 × ULN:<br>Hold study drug/study regimen dose until resolution to Grade ≤1 or baseline<br>Resume study drug/study regimen if elevations downgrade to Grade ≤1 or baseline within 14 days and after completion of steroid taper.<br>Permanently discontinue study drug/study regimen if the elevations do not	Promptly initiate empiric IV methylprednisolone at 1-2 mg/kg/day or equivalent.<br>If still no improvement within 2 to 3 days despite 1-2 mg/kg/day methylprednisolone IV or equivalent, promptly start treatment with an immunosuppressant therapy (i.e., mycophenolate mofetil 0.5 – 1g every 12 hours then taper in consultation with hepatology consult). Discuss with study physician if mycophenolate is not available. **Infliximab should NOT be used.**<br>Perform hepatology consult, abdominal workup, and imaging as appropriate.

	downgrade to Grade ≤1 or baseline within 14 days<br>For elevations in transaminases >8 × ULN or elevations in bilirubin<br>>5 × ULN, permanently discontinue study drug/study regimen.	
Grade 4	Permanently discontinue durvalumab	

If transaminase rise is not isolated but (at any time) occurs in setting of either increasing total/direct bilirubin (≥1.5×ULN, if normal at baseline; or 2×baseline, if >ULN at baseline) or signs of DILI/liver decompensation (e.g., fever, elevated INR):

- Manage dosing for Grade 1 transaminase rise as instructed for Grade 2 transaminase rise
- Manage dosing for Grade 2 transaminase rise as instructed for Grade 3 transaminase rise
- Grade 3-4: Permanently discontinue study drug/study regimen

17.4.4 Nephritis or Renal Dysfunction (elevated serum creatinine) Management Algorithm

Consult a nephrologist. Monitor for signs and symptoms that may be related to changes in renal function (e.g. routine urinalysis, elevated serum BUN and creatinine, decreased creatinine clearance, electrolyte imbalance, decrease in urine output, proteinuria, etc.). Participants should be thoroughly evaluated to rule out any alternative aetiology (e.g. disease progression, infections, recent IV contrast, medications, fluid status, etc.). Consider using steroids should in the absence of clear alternative aetiology even for low grade events (Grade 2), in order to prevent potential progression to higher grade event.

CTCAE grade	Dose Modification	Management
Grade 1	No dose modification required.	Monitor serum creatinine weekly and any accompanying symptom<br>Consider symptomatic treatment including hydration, electrolyte replacement, diuretics, etc<br>If improves<br>- Resume its regular monitoring per study protocol.<br>If worsens<br>- Depends on severity, treat as Grade 2-4.
Grade 2	Hold durvalumab until grade 2 resolution to ≤ grade 1<br>- If toxicity worsens then treat as grade 3 or grade 4<br>- If toxicity improves to baseline then treat at next scheduled treatment date<br>- Durvalumab can be resumed at the next scheduled dose once event stabilises to ≤ grade 1 and 5-7 days after completion of steroid taper.	Consider symptomatic treatment including hydration, electrolyte replacement, diuretics, etc.<br>Carefully monitor serum creatinine every 2-3 days and as clinically warranted<br>Consult nephrologist and consider renal biopsy if clinically indicated<br>If event is persistent beyond 3 to 5 days or worsens, promptly start prednisone 1-2 mg/kg/day PO or IV equivalent.<br>If event is not responsive within 3-5 days or worsens, promptly start prednisone at 1-2 mg/kg/day PO or IV equivalent, consider additional workup<br>When event returns to baseline, resume study drug/study regimen and routine serum creatinine monitoring per study protocol.
Grade 3 or 4	Permanently discontinue durvalumab	Carefully monitor serum creatinine<br>Consult nephrologist and consider renal biopsy if clinically indicated<br>If the event is not responsive within 3 to 5 days or worsens despite prednisone at 1-2 mg/kg/day PO or IV equivalent, consider additional workup and prompt treatment with an immunosuppressive in consultation with a nephrologist.

17.4.5 Rash or Dermatitis (including Pemphigoid) Management Algorithm

Monitor for signs and symptoms of dermatitis (rash and pruritus)

HOLD STUDY DRUG IF STEVENS-JOHNSON SYNDROME (SJS), TOXIC EPIDERMAL NECROLYSIS (TEN) OR OTHER SEVERE CUTANEOUS ADVERSE REACTION (SCAR) IS SUSPECTED

PERMANENTLY DISCONTINUE STUDY DRUG IF SJS, TEN OR SCAR IS CONFIRMED.

CTCAE grade	Management	Management
Grade 1	No dose modification required	Consider symptomatic treatment including oral antipruritics (e.g. diphenhydramine or hydroxyzine) and topical therapy (e.g. emollient lotion or institutional standard)
Grade 2	For persistent (>1 week) Grade 2 events, hold scheduled study drug/study regimen until resolution to Grade ≤1 or baseline.<br>- If toxicity worsens, then treat as grade 3	Obtain Dermatology consult<br>Consider symptomatic treatment including oral antipruritics (e.g. diphenhydramine or hydroxyzine) and topical therapy<br>Consider moderate-strength topical steroid<br>If no improvement within 3-5 days or worsens<br>- Discuss with study physician<br>Consider systemic steroids prednisone 0.5 to 1 mg/kg/day or IV equivalent<br>If persistent for >1-2 weeks or recurs<br>Consider skin biopsy
Grade 3 or 4	<u>For Grade 3:</u><br>Hold study drug/study regimen until resolution to Grade ≤ 1 or baseline.<br>• If toxicity improves to Grade ≤ 1 or baseline, then resume study drug/study regimen after completion of steroid taper.<br>• If toxicity worsens, then treat as Grade 4.<br><u>For Grade 4:</u><br>Permanently discontinue durvalumab	Consult dermatology<br>Promptly initiate empiric IV corticosteroids (e.g. methylprednisolone IV or equivalent) at 1-2 mg/kg/day<br>Consider hospitalisation<br>Monitor extent of rash [Rule of Nines]<br>Consider skin biopsy (preferably more than 1) as clinically feasible.<br>Consider, as necessary, discussing with study physician.

17.4.6 Endocrinopathy Management Algorithm

Examples of endocrinopathy include hyperthyroidism, thyroiditis, hypothyroidism, Type 1 diabetes mellitus, hypophysitis, hypopituitarism, and adrenal insufficiency.

Consider consulting an Endocrinologist for endocrine events, and consider discussing with study physician, as needed.

Monitor participants for signs and symptoms of endocrinopathies. Non-specific symptoms include headache, fatigue, behaviour changes, mental status changes, photophobia, visual field cuts, vertigo, abdominal pain, unusual bowel habits, hypotension and weakness.

Participants should be thoroughly evaluated to rule out any alternative aetiology (e.g. disease progression including brain metastases, infections, etc.)

Depending on the suspected endocrinopathy, monitor and evaluate thyroid function tests: TSH, free T3 and free T4 and other relevant endocrine and related labs (e.g., blood glucose and ketone levels, HgA1c).

If a participant experiences an AE that is thought to be possibly of autoimmune nature (e.g. thyroiditis, pancreatitis, hypophysitis, diabetes insipidus), the investigator should send a blood sample for appropriate autoimmune antibody testing.

Investigators should ask participants with endocrinopathies who may require prolonged or continued hormonal replacement, to consult their primary care physicians or endocrinologists about further monitoring and treatment after completion of the study.

**CTCAE grade**	**Dose Modification**	**Management**
Grade 1	No dose modification required.	Monitor patient with appropriate endocrine function tests.<br>For suspected hypophysitis/hypopituitarism, consider consulting an endocrinologist to guide assessment of early-morning ACTH, cortisol, TSH and free T4; also consider gonadotropins, sex hormones, and prolactin levels, as well as cosyntropin stimulation test (though it may not be useful in diagnosing early secondary adrenal insufficiency).<br>If TSH < 0.5 × LLN, or TSH >2 × ULN, or consistently out of range in 2 subsequent measurements, include free T4 at subsequent cycles as clinically indicated and consider consultation of an endocrinologist.

Grade 2, 3 or 4	For Grade 2-4 endocrinopathies other than hypothyroidism and Type 1 diabetes mellitus, consider holding study drug/study regimen dose until acute symptoms resolve.<br><br>Study drug/study regimen can be resumed once patient stabilises and after completion of steroid taper.<br><br>Patients with endocrinopathies who may require prolonged or continued steroid replacement (e.g., adrenal insufficiency) can be retreated with study drug/study regimen if patient is clinically stable as per investigator or treating physician's clinical judgement.<br><br>If toxicity worsens, then treat based on severity.	Consult endocrinologist to guide evaluation of endocrine function and, as indicated by suspected endocrinopathy and as clinically indicated, consider pituitary scan.<br><br>For all patients with abnormal endocrine work up, except those with isolated hypothyroidism or Type 1 DM, and as guided by an endocrinologist, consider short-term corticosteroids (e.g., 1 to 2 mg/kg/day methylprednisolone or IV equivalent) and prompt initiation of treatment with relevant hormone replacement (e.g., hydrocortisone, sex hormones).<br><br>Isolated hypothyroidism may be treated with replacement therapy, without study drug/study regimen interruption, and without corticosteroids.<br><br>Isolated Type 1 diabetes mellitus (DM) may be treated with appropriate diabetic therapy and without corticosteroids. Only hold study drug/study regimen in setting of hyperglycaemia when diagnostic workup is positive for diabetic ketoacidosis.<br><br>For patients with normal endocrine workup (laboratory assessment or MRI scans), repeat laboratory assessments/MRI as clinically indicated.

17.4.6.1 Amylase/Lipase Increased

CTCAE grade	Dose Modification	Management
Grade 1	No dose modifications.	For modest asymptomatic elevations in serum amylase and lipase, corticosteroid treatment is not indicated as long as there are no other signs or symptoms of pancreatic inflammation.<br>If isolated elevation of enzymes without evidence of pancreatitis, continue immunotherapy.<br>Assess for signs/symptoms of pancreatitis<br>Consider appropriate diagnostic testing (e.g. abdominal CT with contrast, MRCP if clinical suspicion of pancreatitis and no radiologic evidence on CT)<br>If evidence of pancreatitis, manage according to pancreatitis recommendations.
Grade 2, 3 or 4	In consultation with relevant pancreatic specialist, consider continuing study drug/regiment if no clinical/radiologic evidence of pancreatitis +/- improvement in amylase/lipase.	

17.4.6.2 Acute Pancreatitis

Consider Gastroenterology referral.

CTCAE grade	Dose Modification	Management
Grade 1	No dose modifications.	IV hydration<br>Manage as per amylase/lipase increased (asymptomatic)
Grade 2, 3 or 4	<u>For Grade 2:</u><br>Hold study drug/study regimen dose until resolution to Grade ≤1.<br>Consider resumption of study drug/study regimen if no clinical/radiologic evidence of pancreatitis +/- improvement in amylase/lipase in consultation with the relevant pancreatic specialist.<br><u>For Grade 3 or 4:</u><br>Permanently discontinue study drug/study regimen	Promptly start systemic steroids prednisone 1-2 mg/kg/day PO or IV equivalent<br><br>IV hydration

17.4.7 Immune-mediated Neurotoxicity (to include, but not be limited to non-infectious meningitis, non-infectious encephalitis and automatic neuropathy, excluding Myasthenia Gravis and Guillain-Barre) Management Algorithm

Participants should be evaluated to rule out any alternative aetiology (e.g. disease progression, infections, metabolic syndromes and medications, etc.)

Monitor participant for general symptoms (headache, nausea, vertigo, behavior change, or weakness).

Consider appropriate diagnostic testing (e.g. electromyogram and nerve conduction investigations).

Perform symptomatic treatment with Neurology consult as appropriate.

FOR TRANSVERSE MYELITIS, PERMANENTLY DISCONTINUE FOR ANY GRADE.

CTCAE grade	Dose Modification	Management
Grade 1	No dose modification required	
Grade 2	- For acute motor neuropathies or neurotoxicity, hold durvalumab until resolution to ≤ grade 1<br>- For sensory neuropathy/neuropathic pain, consider holding durvalumab until resolution to ≤ grade 1<br>- Permanently discontinue durvalumab if Grade 2 imAE does not resolve to Grade <1 within 30 days<br>- If toxicity worsens, then treat as grade 3 or grade 4	Consider, as necessary, discussing with study physician<br>Obtain Neurology consult<br>Sensory neuropathy/neuropathic pain may be managed by appropriate medications (e.g. gabapentin, duloxetine, etc.)<br>Promptly start systemic steroids prednisone 1-2mg/kg/day PO or IV equivalent<br>If no improvement within 2-3 days, consider additional workup and promptly treat with an additional immunosuppressive therapy (e.g. IV IG or other immunosuppressive depending on the specific imAE)
Grade 3 or 4	Permanently discontinue durvalumab	Consider, as necessary,<br>discussing with study<br>physician<br>Obtain Neurology consult<br>Consider hospitalisation<br>Promptly initiate empiric IV methylprednisolone 1-2 mg/kg/day or equivalent<br>If no improvement within 2-3 days, consider additional workup and promptly treat with an additional immunosuppressive depending on the specific imAE<br>Once stable, gradually taper steroids over ≥28 days

17.4.8 Immune-mediated Peripheral Neuromotor Syndromes, such as Guillain-Barre and Myasthenia Gravis, Management Algorithm

The prompt diagnosis of immune-mediated peripheral neuromotor syndromes is important, since certain participants may unpredictably experience acute decompensation which can result in substantial morbidity or in the worst case, death. Special care should be taken for certain sentinel symptoms which may predict a more severe outcome, such as prominent dysphagia, rapidly progressive weakness, and signs of respiratory insufficiency or autonomic instability

Participants should be evaluated to rule out any alternative aetiology (e.g. disease progression, infections, metabolic syndromes and medications, etc.). It should be noted that the diagnosis of immune-mediated peripheral neuromotor syndromes can be particularly challenging in participants with underlying cancer, due to the multiple potential confounding effects of cancer (and its treatments) throughout the neurological system. Given the importance of prompt and accurate diagnosis, it is essential to have a low threshold to obtain a neurological consult.

Neurophysiologic diagnostic testing (e.g. electromyogram and nerve conduction investigations, and "repetitive stimulation" if myasthenia is suspected) are routinely indicated upon suspicion of such conditions and may be best facilitated by means of a neurology consultation.

It is important to consider that the use of steroids as the primary treatment of Guillain-Barre is not typically considered effective. Participants requiring treatment should be considered for plasmapheresis (or IVIG, as an alternative).

CTCAE grade	Dose Modification	Management
Grade 1	No dose modification required.	Consider discussing with the study physician, as needed.<br>Care should be taken to monitor participants for sentinel symptoms of a potential decompensation as described above<br>Consult a Neurologist.
Grade 2	Hold durvalumab until resolution to ≤ grade 1.<br>- Permanently discontinue durvalumab if it does not resolve to ≤ grade 1 within 30 days or if there are signs of respiratory insufficiency or autonomic instability	Consider discussing with study physician, as needed.<br>Care should be taken to monitor participants for sentinel symptoms of a potential decompensation as described above<br>Consult a Neurologist<br>Sensory neuropathy/neuropathic pain may be managed by appropriate medications (e.g. gabapentin, duloxetine, etc.)<br>*MYASTHENIA GRAVIS:*<br>Steroids may be successfully used to treat Myasthenia Gravis. Important to consider that steroid therapy (especially with high doses) may result in transient worsening of myasthenia and should typically be administered in a monitored setting under supervision of a consulting neurologist.<br>Participants unable to tolerate steroids may be candidates for treatment with

<table>
<tr><td></td><td></td><td>plasmapheresis or IVIG. Such decisions are best made in consultation with a neurologist, taking into account the unique needs of each patient.

If Myasthenia Gravis-like neurotoxicity present, consider starting acetylcholine esterase (AChE) inhibitor therapy in addition to steroids. Such therapy, if successful, can also serve to reinforce the diagnosis.

Avoid medications that can worsen myasthenia gravis

GUILLAIN-BARRE:

Important to consider here that the use of steroids as the primary treatment of Guillain-Barre is not typically considered effective. Participants requiring treatment should be started with IV IG and followed by plasmapheresis if not responsive to IV IG.</td></tr>
<tr><td>Grade 3</td><td>Hold durvalumab until resolution to ≤ grade 1

– Permanently discontinue durvalumab if Grade 3 imAE does not resolve to ≤ grade 1 within 30 days or if there are signs of respiratory insufficiency or autonomic instability.</td><td rowspan="2">Consider discussing with

study physician, as needed

Recommend hospitalisation

Monitor symptoms and obtain neurological consult

MYASTHENIA GRAVIS:

Steroids may be successfully used to treat Myasthenia Gravis. It should be administered in a monitored setting under supervision of a consulting neurologist.

Participants unable to tolerate steroids may be candidates for treatment with plasmapheresis or IVIG.

If Myasthenia Gravis-like neurotoxicity present, consider starting acetylcholine esterase (AChE) inhibitor therapy in addition to steroids. Such therapy, if successful, can also serve to reinforce the diagnosis.

Avoid medications that can worsen myasthenia gravis

GUILLAIN-BARRE:

Important to consider here that the use of steroids as the primary treatment of Guillain-Barre is not typically considered effective. Participants requiring treatment should be started with IV IG and followed by plasmapheresis if not responsive to IV IG.</td></tr>
<tr><td>Grade 4</td><td>Permanently discontinue durvalumab.</td></tr>
</table>

17.4.9 Myocarditis Management Algorithm

The prompt diagnosis of immune-mediated myocarditis is important, particularly in patients with baseline cardiopulmonary disease and reduced cardiac function.

Consider discussing with the study physician, as needed.

Monitor patients for signs and symptoms of myocarditis (new onset or worsening chest pain, arrhythmia, shortness of breath, peripheral edema). As some symptoms can overlap with lung toxicities, simultaneously evaluate for and rule out pulmonary toxicity as well as other causes (e.g., pulmonary embolism, congestive heart failure, malignant pericardial effusion). Consult a Cardiologist early to promptly assess whether and when to complete a cardiac biopsy, including any other diagnostic procedures.

Initial work-up should include clinical evaluation, BNP, cardiac enzymes, ECG, echocardiogram (ECHO), monitoring of oxygenation via pulse oximetry (resting and exertion), and additional laboratory work-up as indicated. Spiral CT or cardiac MRI can complement ECHO to assess wall motion abnormalities when needed.

Discontinue drug permanently if biopsy-proven immune-mediated myocarditis.

CTCAE grade	Dose Modification	Management
Grade 1	No dose modifications required unless clinical suspicion is high, in which case hold study drug/study regimen dose during diagnostic work-up for other aetiologies. If study drug/study regimen is held, resume after complete resolution to Grade 0.	Monitor and closely follow up in 2 to 4 days for clinical symptoms, BNP, cardiac enzymes, ECG, ECHO, pulse oximetry (resting and exertion), and laboratory work-up as clinically indicated.<br>Consider using steroids if clinical suspicion is high.
Grade 2, 3 or 4	If Grade 2 - Hold study drug/study regimen dose until resolution to Grade 0. If toxicity rapidly improves to Grade 0, then the decision to reinitiate study drug/study regimen will be based upon treating physician’s clinical judgment and after completion of steroid taper. If toxicity does not rapidly improve, permanently discontinue study drug/study regimen.<br>If Grade 3-4, permanently discontinue study drug/study regimen.	Monitor symptoms daily, hospitalize.<br>Promptly start IV methylprednisolone 2 to 4 mg/kg/day or equivalent after Cardiology consultation has determined whether and when to complete diagnostic procedures including a cardiac biopsy.<br>Supportive care (e.g., oxygen).<br>If no improvement within 2 to 3 days despite IV methylprednisolone at 2 to 4 mg/kg/day, promptly start immunosuppressive therapy such as TNF inhibitors (e.g., infliximab at 5 mg/kg IV, may be repeated at 2 and 6 weeks after initial dose at the discretion of the treating provider). **Caution: It is important to rule out sepsis and refer to infliximab label for general guidance before using infliximab. Infliximab is contraindicated for patients who have heart failure.**

17.4.10 Myositis/Polymyositis Management Algorithm

Monitor patients for signs and symptoms of poly/myositis. Typically, muscle weakness/pain occurs in proximal muscles including upper arms, thighs, shoulders, hips, neck and back, but rarely affects the extremities including hands and fingers; also difficulty breathing and/or trouble swallowing can occur and progress rapidly. Increased general feelings of tiredness and fatigue may occur, and there can be new-onset falling, difficulty getting up from a fall, and trouble climbing stairs, standing up from a seated position, and/or reaching up.

If poly/myositis is suspected, a Neurology consultation should be obtained early, with prompt guidance on diagnostic procedures. Myocarditis may co-occur with poly/myositis; refer to guidance under Myocarditis. Given breathing complications, refer to guidance under Pneumonitis/ILD. Given possibility of an existent (but previously unknown) autoimmune disorder, consider Rheumatology consultation.

Consider, as necessary, discussing with the study physician.

Initial work-up should include clinical evaluation, creatine kinase, aldolase, LDH, BUN/creatinine, erythrocyte sedimentation rate or C-reactive protein level, urine myoglobin, and additional laboratory work-up as indicated, including a number of possible rheumatological/antibody tests (i.e., consider whether a rheumatologist consultation is indicated and could guide need for rheumatoid factor, antinuclear antibody, anti-smooth muscle, antisynthetase [such as anti-Jo-1], and/or signal-recognition particle antibodies). Confirmatory testing may include electromyography, nerve conduction studies, MRI of the muscles, and/or a muscle biopsy. Consider Barium swallow for evaluation of dysphagia or dysphonia.

Patients should be thoroughly evaluated to rule out any alternative etiology (e.g., disease progression, other medications, or infections).

**CTCAE grade**	**Dose Modification**	**Management**
Grade 1	No dose modifications.	Monitor and closely follow up in 2 to 4 days for clinical symptoms and initiate evaluation as clinically indicated.<br>Consider Neurology consult.<br>Consider, as necessary, discussing with the study physician.
Grade 2	Hold study drug/study regimen dose until resolution to Grade ≤1.<br>Permanently discontinue study drug/study regimen if it does not resolve to Grade ≤1 within 30 days or if there are signs of respiratory insufficiency.	Monitor symptoms daily and consider hospitalisation.<br>Obtain Neurology consult, and initiate evaluation.<br>Consider, as necessary, discussing with the study physician.<br>If clinical course is rapidly progressive (particularly if difficulty breathing and/or trouble swallowing), promptly start IV methylprednisolone 2 to 4 mg/kg/day systemic steroids along with receiving input from Neurology consultant<br>If clinical course is not rapidly progressive, start systemic steroids (e.g., prednisone 1-2 mg/kg/day PO or IV equivalent); if no improvement within 3 to 5 days, continue additional work up and start treatment with IV methylprednisolone 2 to 4 mg/kg/day<br>If after start of IV methylprednisolone

		at 2 to 4 mg/kg/day there is no improvement within 2 to 3 days consider starting another immunosuppressive therapy such as TNF inhibitors (e.g. infliximab at 5 mg/kg IV, may be repeated at 2 and 6 weeks after initial dose at the discretion of the treating provider). Caution: It is important to rule out sepsis and refer to infliximab label for general guidance before using infliximab.
Grade 3 or 4<br><br>(pain associated with severe weakness; limiting self-care ADLs)	For Grade 3:<br><br>Hold study drug/study regimen dose until resolution to Grade ≤1.<br><br>Permanently discontinue study drug/study regimen if Grade 3 imAE does not resolve to Grade ≤1 within 30 days or if there are signs of respiratory insufficiency.<br><br>For Grade 4:<br><br>Permanently discontinue study drug/study regimen.	Monitor symptoms closely; recommend hospitalization.<br><br>Obtain Neurology consult, and complete full evaluation<br><br>Consider discussing with the study physician, as needed<br><br>Promptly start IV methylprednisolone 2 to 4 mg/kg/day systemic steroids along with receiving input from Neurology consultant.<br><br>If after start of IV methylprednisolone at 2 to 4 mg/kg/day there is no improvement within 2 to 3 days, consider starting another immunosuppressive therapy such as TNF inhibitors<br>(e.g., infliximab at 5 mg/kg IV, may be repeated at 2 and 6 weeks after initial dose at the discretion of treating provider). **Caution: It is important to rule out sepsis and refer to infliximab label for general guidance before using infliximab.**<br><br>Consider whether patient may require IV IG, plasmapheresis.

17.4.11 Other Immune-Mediated Reactions

CTCAE grade	Dose Modification	Management
Any Grade	Note: it is possible that events with an inflammatory or immune-mediated mechanism could occur in nearly all organs, some of them are not noted specifically in these guidelines (e.g. immune thrombocytopaenia, haemolytic anaemia, uveitis, vasculitis)	– The study physician may be contacted for immune-mediated reactions not listed in the "specific immune-mediated reactions" section<br>– Thorough evaluation to rule out any alternative etiology (e.g. disease progression, concomitant medications, and infections)<br>– Consultation with relevant specialist<br>– Treat accordingly, as per institutional standard.
Grade 1	No dose modifications.	Monitor as clinically indicated.
Grade 2	• Hold study drug/study regimen until resolution to ≤Grade 1 or baseline.<br>• If toxicity worsens, then treat as Grade 3 or Grade 4.<br>• Study drug/study regimen can be resumed once event stabilises to Grade ≤1 after completion of steroid taper.<br>• Consider whether study drug/study regimen should be permanently discontinued in Grade 2 events with high likelihood for morbidity and/or mortality when they do not rapidly improve to Grade <1 upon treatment with systemic steroids and following full taper.	Treat accordingly, as per institutional standard, appropriate clinical practice guidelines, and other society guidelines (e.g. NCCN, ESMO)
Grade 3	Hold study drug/study regimen.	
Grade 4	Permanently discontinue study drug/study regimen.	

[a] ASCO Educational Book 2015 "Managing Immune Checkpoint Blocking Antibody Side Effects" by Michael Postow MD.
[b] FDA Liver Guidance Document 2009 Guidance for Industry: Drug Induced Liver Injury – Premarketing Clinical Evaluation.
[c] NCCN Clinical Practice Guidelines in Oncology "Management of Immunotherapy-Related Toxicities" Version 1.2020 – December 2019

Abbreviations: AChE Acetylcholine esterase; ADL Activities of daily living; AE Adverse event; ALP Alkaline phosphatase test; ALT Alanine aminotransferase; AST Aspartate aminotransferase; BUN Blood urea nitrogen; CT Computed tomography; CTCAE Common Terminology Criteria for Adverse Events; ESMO European Society for Medical Oncology; ILD Interstitial lung disease; imAE immune-mediated adverse event; IG Immunoglobulin; IV Intravenous; GI Gastrointestinal; LFT Liver function tests; LLN Lower limit of normal; MRI Magnetic resonance imaging; NCI National Cancer Institute; NCCN National Comprehensive Cancer Network; PJP Pneumocystis jirovecii pneumonia (formerly known as Pneumocystis carinii pneumonia); PO By mouth; T3 Triiodothyronine; T4 Thyroxine; TB Total bilirubin; TNF Tumor necrosis factor; TSH Thyroid-stimulating hormone; ULN Upper limit of normal.

17.5 Appendix 5 Inhibitors and Inducers of CYP3A

17.5.1 CYP3A4 INDUCERS (PROHIBITED)

Armodafenil[1]<br>Barbiturates[2]<br>Bosentan[1]<br>Carbamazepine<br>Efavirenz<br>Fosphenytoin[1]	Modafinil[2]<br>Nafcillin[1]<br>Nevirapine<br>Oxcarbazepine<br>Pentobarbital[1]<br>Phenobarbital<br>Phenytoin<br>Pioglitazone[2]	Primidone[1]<br>Rifabutin<br>Rifampin<br>Rifapentine[1]<br>St. John's wort[2]<br>Troglitazone[3]

1. Cited in Cytochrome P450 Enzymes: Substrates, Inhibitors, and Inducers. In: Lacy CF, Armstrong LL, Goldman MP, Lance LL, eds. Drug Information Handbook 20th ed. Hudson, OH; LexiComp Inc. 2011-2012: 1810-1818
2. Cited in Flockhart DA. Drug Interactions: Cytochrome P450 Drug Interaction Table. Indiana University School of Medicine (2007). http://medicine.iupui.edu/clinpharm/ddis/table.asp. Accessed Nov 2011.
3. Weak inhibitor per Lacy et al. May be used with caution.

Drugs without a superscript are cited in both the Lacy and Flockhart references.

17.5.2 CYP3A4 INHIBITORS

**Strong Inhibitors** (prohibited)	**Moderate Inhibitors** (use with caution, avoid if possible)	**Weak Inhibitors** (use with caution, avoid if possible)
Amprenavir[1]<br>Atazanavir[1]<br>Clarithromycin<br>Conivaptan[1]<br>Delavirdine[1]<br>Fosamprenavir[1]<br>Fospropofol[1]<br>Imatinib[1]<br>Indinavir<br>Isoniazid[1]<br>Itraconazole<br>Ketoconazole<br>Miconazole[1]<br>Nefazodone<br>Nelfinavir<br>Nicardipine[1]<br>Posaconazole[1]<br>Propofol[1]<br>Quinidine[1]<br>Ritonavir<br>Saquinavir[2]<br>Telithromycin	Amiodarone[1]<br>Aprepitant<br>Cimetidine[1]<br>Clotrimazole[1]<br>Cyclosporine[1]<br>Desipramine[1]<br>Doxycycline[1]<br>Efavirenz[1]<br>Erythromycin<br>Fluconazole<br>Fosaprepitant[1]<br>Grapefruit juice<br>Haloperidol[1]<br>Lidocaine[1]<br>Metronidazole[1]<br>Norfloxacin[1]<br>Sertraline[1]<br>Tetracycline[1]<br>Verapamil<br>Voriconazole[1]	Chloramphenicol[2]<br>Ciprofloxacin[2]<br>Diethyldithiocarbamate[2]<br>Fluvoxamine[2]<br>Gestodene[2]<br>Mibefradil[2]<br>Mifepristone<br>Norfluoxetine[2]<br>Star fruit[2]<br>Troleandomycin[2]

1. Cited in Cytochrome P450 Enzymes: Substrates, Inhibitors, and Inducers. In: Lacy CF, Armstrong LL, Goldman MP, Lance LL, eds. Drug Information Handbook 20th ed. Hudson, OH; LexiComp Inc. 2011-2012: 1810-1818
2. Cited in Flockhart DA. Drug Interactions: Cytochrome P450 Drug Interaction Table. Indiana University School of Medicine (2007). http://medicine.iupui.edu/clinpharm/ddis/table.asp. Accessed Nov 2011.

Drugs without a superscript are cited in both the Lacy and Flockhart references.

17.5.3 CYP3A4 Substrates (Allowed – take note of possible interactions)

Alfentanil	Dexlansoprazole	Isradipine	Ranolazine
Alfuzosin	Dextromethorphan[2]	Itraconazole	Rifabutin
Alprazolam	Diazepam	Ixabepilone	Repaglinide
Ambrisentan	Dihydroergotamine	Ketamine	Risperidone[2]
Amiodarone	Diltiazem	Ketoconazole	Ritonavir
Amlodipine	Disopyramide	Lansoprazole	Salmeterol
Aprepitant	Docetaxel	Lapatinib	Saquinavir
Aripiprazole	Domperidone[2]	Lercanidipine[2]	Sibutramine
Armodafinil	Doxorubicin	Levonorgestrel	Sildenafil
Astemizole[2]	Eletriptan	Lidocaine	Simvastatin
Atazanavir	Efavirenz	Lovastatin[2]	Sirolimus
Atorvastatin	Eplerenone	Lopinavir	Solifenacin
Benzphetamine	Ergoloid mesylates	Maraviroc	Spiramycin
Bisoprolol	Ergonovine	Medroxyprogesterone	Sufentanil
Bortezomib	Ergotamine	Mefloquine	Sunitinib
Bosentan	Erlotinib	Mestranol	Tacrolimus
Bromazepam	Erythromycin	Methadone	Tadalafil
Bromocriptine	Escitalopram	Methylergonovine	Tamoxifen
Budesonide	Esomeprazole	Methylprednisolone	Tamsulosin
Buprenorphine	Estradiol	Miconazole	Temsirolimus
Buspirone	Estrogens, conjugated	Midazolam	Telithromycin
Busulfan	synthetic	Mirtazapine	Teniposide
Cafergot[2]	Estrogens, conjugated	Modafinil	Terfenadine[2]
Caffeine[2]	equine	Montelukast	Testosterone[2]
Carbamazepine	Estrogens, esterified	Nateglinide	Tetracycline
Cerivastatin[2]	Estropipate	Nefazodone	Theophylline
Chlordiazepoxide	Eszopiclone	Nelfinavir	Tiagabine
Chloroquine	Ethinyl estradiol	Nevirapine	Ticlopidine
Chlorpheniramine	Ethosuximide	Nicardipine	Tinidazole
Ciclesonide	Etoposide	Nifedipine	Tipranavir
Cilostazol	Exemestane	Nilotinib	Tolterodine
Cisapride	Felbamate	Nimodipine	Toremifene
Citalopram	Felodipine	Nitrendipine[2]	Tramadol
Clarithromycin	Fentanyl	Nisoldipine	Trazodone
Clobazam	Finasteride[2]	Norethindrone	Triazolam
Clonazepam	Flunisolide	Norgestrel	Trimethoprim
Clorazepate	Flurazepam	Omeprazole	Trimipramine
Cocaine	Flutamide	Ondansetron	Vardenafil
Codeine[2]	Fluticasone	Paclitaxel	Venlafaxine
Colchicine	Fosamprenavir	Paricalcitol	Verapamil
Conivaptan	Fosaprepitant	Pazopanib	Vinblastine
Cyclophosphamide	Gefitinib	Pimozide	Vincristine
Cyclosporine	Haloperidol	Primaquine	Vinorelbine
Dantrolene	Hydrocortisone[2]	Propranolol[2]	Zaleplon[2]
Dapsone	Ifosfamide	Progesterone	Ziprasidone[2]
Darifenacin	Imatinib	Quazepam	Zolpidem
Darunavir	Indinavir	Quetiapine	Zonisamide
Dasatinib	Irinotecan	Quinidine	Zopiclone

17.5.3 CYP3A4 Substrates (Allowed – take note of possible interactions)

Delavirdine<br>Dexamethasone	Isosorbide dinitrate<br>Isosorbide mononitrate	Quinine<br>Rabeprazole	

Notes: CYP3A4 Substrate Table adapted from Cytochrome P450 Enzymes: Substrates, Inhibitors, and Inducers. In: Lacy CF, Armstrong LL, Goldman MP, Lance LL, eds. Drug Information Handbook 20th ed. Hudson, OH; LexiComp Inc. 2011-2012: 1810-1818

Substrates with 2 superscript denote substrates cited in Flockhart DA. Drug Interactions: Cytochrome P450 Drug Interaction Table. Indiana University School of Medicine (2007). http://medicine.iupui.edu/clinpharm/ddis/table.asp. Accessed Nov 2011

17.6 Appendix 6 EORTC QLQ-C30 Questionnaire

ENGLISH

EORTC QLQ-C30 (version 3)

We are interested in some things about you and your health. Please answer all of the questions yourself by circling the number that best applies to you. There are no "right" or "wrong" answers. The information that you provide will remain strictly confidential.

Please fill in your initials:
Your birthdate (Day, Month, Year):
Today's date (Day, Month, Year): 31

	Not at All	A Little	Quite a Bit	Very Much
1. Do you have any trouble doing strenuous activities, like carrying a heavy shopping bag or a suitcase?	1	2	3	4
2. Do you have any trouble taking a long walk?	1	2	3	4
3. Do you have any trouble taking a short walk outside of the house?	1	2	3	4
4. Do you need to stay in bed or a chair during the day?	1	2	3	4
5. Do you need help with eating, dressing, washing yourself or using the toilet?	1	2	3	4

**During the past week:**	Not at All	A Little	Quite a Bit	Very Much
6. Were you limited in doing either your work or other daily activities?	1	2	3	4
7. Were you limited in pursuing your hobbies or other leisure time activities?	1	2	3	4
8. Were you short of breath?	1	2	3	4
9. Have you had pain?	1	2	3	4
10. Did you need to rest?	1	2	3	4
11. Have you had trouble sleeping?	1	2	3	4
12. Have you felt weak?	1	2	3	4
13. Have you lacked appetite?	1	2	3	4
14. Have you felt nauseated?	1	2	3	4
15. Have you vomited?	1	2	3	4
16. Have you been constipated?	1	2	3	4

Please go on to the next page

During the past week:

	Not at All	A Little	Quite a Bit	Very Much
17. Have you had diarrhea?	1	2	3	4
18. Were you tired?	1	2	3	4
19. Did pain interfere with your daily activities?	1	2	3	4
20. Have you had difficulty in concentrating on things, like reading a newspaper or watching television?	1	2	3	4
21. Did you feel tense?	1	2	3	4
22. Did you worry?	1	2	3	4
23. Did you feel irritable?	1	2	3	4
24. Did you feel depressed?	1	2	3	4
25. Have you had difficulty remembering things?	1	2	3	4
26. Has your physical condition or medical treatment interfered with your family life?	1	2	3	4
27. Has your physical condition or medical treatment interfered with your social activities?	1	2	3	4
28. Has your physical condition or medical treatment caused you financial difficulties?	1	2	3	4

For the following questions please circle the number between 1 and 7 that best applies to you

29. How would you rate your overall health during the past week?

1	2	3	4	5	6	7
Very poor						Excellent

30. How would you rate your overall quality of life during the past week?

1	2	3	4	5	6	7
Very poor						Excellent

© Copyright 1995 EORTC Quality of Life Group. All rights reserved. Version 3.0

17.7 Appendix 7 EORTC OV28 Questionnaire

EORTC QLQ - OV28

Patients sometimes report that they have the following symptoms or problems. Please indicate the extent to which you have experienced these symptoms or problems during the past week.

**During the past week:**	Not at All	A Little	Quite a Bit	Very Much
31. Did you have abdominal pain?	1	2	3	4
32. Did you have a bloated feeling in your abdomen / stomach?	1	2	3	4
33. Did you have problems with your clothes feeling too tight?	1	2	3	4
34. Did you experience change in bowel habit as a result of your disease or treatment?	1	2	3	4
35. Were you troubled by passing wind / gas / flatulence?	1	2	3	4
36. Have you felt full up too quickly after beginning to eat?	1	2	3	4
37. Have you had indigestion or heartburn?	1	2	3	4
38. Have you lost any hair?	1	2	3	4
39. Answer this question only if you had any hair loss: Were you upset by the loss of your hair?	1	2	3	4
40. Did food and drink taste different from usual?	1	2	3	4
41. Have you had tingling hands or feet?	1	2	3	4
42. Have you had numbness in your fingers or toes?	1	2	3	4
43. Have you felt weak in your arms or legs?	1	2	3	4
44. Did you have aches or pains in your muscles or joints?	1	2	3	4
45. Did you have problems with hearing?	1	2	3	4
46. Did you urinate frequently?	1	2	3	4
47. Have you had skin problems (e.g. itchy, dry)?	1	2	3	4
48. Did you have hot flushes?	1	2	3	4
49. Did you have night sweats?	1	2	3	4

Please go on to next page

**During the past week:**	**Not at All**	**A Little**	**Quite a Bit**	**Very Much**
50. Have you felt physically less attractive as a result of your disease or treatment?	1	2	3	4
51. Have you been dissatisfied with your body?	1	2	3	4
52. How much has your disease been a burden to you?	1	2	3	4
53. How much has your treatment been a burden to you?	1	2	3	4
54. Were you worried about your future health?	1	2	3	4

**During the past 4 weeks:**	**Not at All**	**A Little**	**Quite a Bit**	**Very Much**
55. To what extent were you interested in sex?	1	2	3	4
56. To what extent were you sexually active?	1	2	3	4
Answer the following two questions only if you were sexually active:				
57. To what extent was sex enjoyable for you?	1	2	3	4
58. Did you have a dry vagina during sexual activity?	1	2	3	4

© Copyright 1997 EORTC Quality of Life Group. All rights reserved. (phase III module)

17.8 Appendix 8 MOST Questionnaire

SOLACE2
(CTC 0178 / ANZGOG 1723/2018)

Patient No. ☐☐☐ / ☐☐☐ Visit No.

Measure of Ovarian Cancer Symptoms & Treatment Concerns – Recent

Patient initials: ☐☐☐ (First middle last) Today's date: ☐☐ (D D) ☐☐ (M M) ☐☐ (Y Y)

Please **circle** one number for each line to best show **how much that aspect troubled you on average <u>during the last month</u>.**

	No trouble at all		Mild			Moderate			Severe		Worst I can imagine
1. Pain (all and anywhere)	0	1	2	3	4	5	6	7	8	9	10
2. Fatigue (tiredness)	0	1	2	3	4	5	6	7	8	9	10
3. Poor appetite (or feeling full quickly)	0	1	2	3	4	5	6	7	8	9	10
4. Abdominal pain, discomfort and/or cramps	0	1	2	3	4	5	6	7	8	9	10
5. Abdominal swelling, bloating and/or fullness	0	1	2	3	4	5	6	7	8	9	10
6. Trouble eating	0	1	2	3	4	5	6	7	8	9	10
7. Indigestion	0	1	2	3	4	5	6	7	8	9	10
8. Nausea	0	1	2	3	4	5	6	7	8	9	10
9. Vomiting	0	1	2	3	4	5	6	7	8	9	10
10. Diarrhoea	0	1	2	3	4	5	6	7	8	9	10
11. Constipation	0	1	2	3	4	5	6	7	8	9	10
12. Bladder problems	0	1	2	3	4	5	6	7	8	9	10
13. Shortness of breath	0	1	2	3	4	5	6	7	8	9	10
14. Leg swelling	0	1	2	3	4	5	6	7	8	9	10
15. Trouble sleeping	0	1	2	3	4	5	6	7	8	9	10

Please circle one number for each line to show how you would have rated yourself on that aspect on average **<u>during the last month</u>**.

	Best possible	Very Good		Good		Fair		Poor		Very poor	Worst possible
16. Physical well-being	10	9	8	7	6	5	4	3	2	1	0
17. Emotional well-being	10	9	8	7	6	5	4	3	2	1	0
18. Overall well-being	10	9	8	7	6	5	4	3	2	1	0

Version 1.0 NHMRC Clinical Trials Centre, University of Sydney

SOLACE2
(CTC 0178 / ANZGOG 1723/2018)

Patient No. [| |] / [| |] Visit No.

Measure of Ovarian Cancer Symptoms & Treatment Concerns – Recent

Patient initials: [| |] Study # [| | | | | |] Today's date: [|] [|] [|]
First middle last — D D — M M — Y Y

Please circle one number for each line to show how you would have rated yourself on that aspect on average **during the last month**.

	No trouble at all		Mild			Moderate			Severe		Worst I can imagine
19. Altered sense of taste	0	1	2	3	4	5	6	7	8	9	10
20. Sore mouth or throat	0	1	2	3	4	5	6	7	8	9	10
21. Difficulty swallowing	0	1	2	3	4	5	6	7	8	9	10
22. Loss of appetite	0	1	2	3	4	5	6	7	8	9	10
23. Hair loss	0	1	2	3	4	5	6	7	8	9	10
24. Skin rash	0	1	2	3	4	5	6	7	8	9	10
25. Numbness or pins and needles	0	1	2	3	4	5	6	7	8	9	10
26. Sore hands and feet	0	1	2	3	4	5	6	7	8	9	10
27. Problems taking tablets	0	1	2	3	4	5	6	7	8	9	10
28. Problems with needles or injections	0	1	2	3	4	5	6	7	8	9	10
29. Inconvenience of treatment	0	1	2	3	4	5	6	7	8	9	10
30. Thought of actually having treatment	0	1	2	3	4	5	6	7	8	9	10
31. Trouble concentrating	0	1	2	3	4	5	6	7	8	9	10
32. Anxiety (feeling worried)	0	1	2	3	4	5	6	7	8	9	10
33. Depression (feeling sad)	0	1	2	3	4	5	6	7	8	9	10
34. Problems doing what I wanted	0	1	2	3	4	5	6	7	8	9	10
35. Problems for my family or friends	0	1	2	3	4	5	6	7	8	9	10

Thank you for filling out this form.

Version 1.0 — NHMRC Clinical Trials Centre, University of Sydney

17.9 Appendix 9 RECIST 1.1 Criteria

Response Evaluation Criteria in Solid Tumours (RECIST v1.1)

These instructions are based on the guidelines recommended in Eisenhauer EA, Therasse P, Bogaerts J et al. New response evaluation criteria in solid tumours: Revised RECIST guideline (version 1.1). (27)

Evaluable for Response

All participants who have received at least one cycle of therapy and have their disease re-evaluated will be considered evaluable for response (exceptions will be those who exhibit objective disease progression prior to the end of cycle 1 who will also be considered evaluable). Participants on therapy for at least this period and who meet the other listed criteria will have their response classified according to the definitions set out below.

17.9.1 Disease and Lesion Definitions

Measurable Disease

Measurable *tumour lesions* are defined as those that can be accurately measured in at least one dimension (longest diameter to be recorded) as

- ≥ 20 mm with chest x-ray
- ≥10 mm with CT scan or clinical examination.
- Bone lesions are considered measurable only if assessed by CT scan and have an identifiable soft tissue component that meets these requirements (soft tissue component ≥ 10 mm by CT scan).

Malignant lymph nodes must be ≥ 15mm in the short axis to be considered measurable; only the short axis will be measured and followed.

All tumour measurements must be recorded in millimetres. Previously irradiated lesions are not considered measurable unless progression has been documented in the lesion.

Non-measurable Disease

All other lesions (or sites of disease), including small lesions are considered non-measurable disease. Bone lesions without a measurable soft tissue component, leptomeningeal disease, ascites, pleural/pericardial effusions, lymphangitis cutis/pulmonis, inflammatory breast disease, lymphangitic involvement of lung or skin and abdominal masses followed by clinical examination are all non-measurable. Lesions in previously irradiated areas are non-measurable, unless progression has been demonstrated.

Target Lesions

When more than one measurable tumour lesion is present at baseline all lesions up to *a maximum of 5 lesions in total* (and a maximum of *2 lesions per organ*) representative of all involved organs should be identified as target lesions and will be recorded and measured at baseline. Target lesions should be selected on the basis of their size (lesions with the longest diameter), be representative of all involved organs, but in addition should be those that lend themselves to *reproducible repeated measurements*.

Note that pathological lymph nodes must meet the criterion of having a short axis of ≥ 15 mm by CT scan and only the *short* axis of these lymph nodes will contribute to the baseline sum. All other pathological lymph nodes (those with a short axis ≥ 10 mm but <15 mm) should be considered non-target lesions. Nodes that have a short axis < 10 mm are considered non-pathological and should not be recorded or followed. At baseline, the sum of the target lesions (longest diameter of tumour lesions plus short axis of target lymph nodes: overall maximum of 5) is to be recorded.

After baseline, a value should be provided on the CRF for all identified target lesions for each assessment, even if very small. If extremely small and faint lesions cannot be accurately measured but are deemed to be present, a default value of 5 mm may be used. If lesions are too small to measure and indeed are believed to be absent, a default value of 0 mm may be used.

Non-target Lesions

All non-measurable lesions (or sites of disease) plus any measurable lesions over and above those listed as target lesions are considered *non-target lesions*. Measurements are not required but these lesions should be noted at baseline and should be followed as “present” or “absent”.

17.9.2 Response Definitions

**Definition**	**Evaluation**
Complete Response (CR)	Disappearance of all target and non-target lesions.<br>Any pathological lymph nodes (whether target or non-target) must have reduction in short axis to <10mm
Partial Response(PR)	At least a 30% decrease in the sum of the diameters of target lesions, taking as reference the baseline sum diameters. Non target lesions must be non-PD.
Stable Disease (SD)	Neither sufficient shrinkage to qualify for PR nor sufficient increase to qualify for PD, taking as reference the smallest sum diameter while on trial
Progressive Disease (PD)	At least a 20% increase in the sum of the diameters of target lesions, taking as reference the smallest sum on trial (this includes the baseline sum if that is the smallest on trial) AND an absolute increase of ≥ 5mm. Appearance of new lesions will also constitute progressive disease (including lesions in previously unassessed areas).<br>In exceptional circumstances, unequivocal progression of non-target disease may be accepted as evidence of disease progression, where the overall tumour burden has increased sufficiently to merit discontinuation of treatment or where the tumour burden appears to have increased by at least 73% in volume. Modest increases in the size of one or more non-target lesions are NOT considered unequivocal progression. If the evidence of PD is equivocal (target or non-target), treatment may continue until the next assessment, but if confirmed, the earlier date must be used.

Patients with measurable disease at baseline

Target lesions	Non-target lesions	New Lesions	Overall Response
CR	CR	No	CR
CR	Non-CR/Non-PD	No	PR
CR	Not all evaluated	No	PR
PR	Non-PD/ not all evaluated	No	PR
SD	Non-PD/ not all evaluated	No	SD
Not all evaluated	Non-PD	No	NE
PD	Any	Any	PD
Any	PD	Any	PD
Any	Any	Yes	PD

Patients with non-target disease only

Non-target lesions	New lesions	Overall response
CR	No	CR
Non-CR/Non-PD	No	Non-CR/Non-PD
Not all evaluated	No	NE
Unequivocal PD	Yes or No	PD
Any	Yes	PD

17.10 Appendix 10 GCIG criteria for CA125 response

The GCIG recommends that for trials of relapsed ovarian cancer the following definition for response according to CA125 be used in addition to the standard RECIST response criteria.(28)

Definition of response – A response according to CA125 has occurred if there is at least a 50% reduction in CA125 levels from a pre-treatment sample. The response must be confirmed and maintained for at least 28 days. Patients can be evaluated according to CA125 only if they have a pre-treatment sample that is at least twice the upper limit of normal and within 2 weeks prior to starting treatment.

Definition of progression or recurrence – Patients with elevated CA125 pre-treatment and normalisation of CA125 must show evidence of CA125 greater than, or equal to, two times the upper normal limit on two occasions.

OR

Patients with elevated CA125 pre-treatment, which never normalises must show evidence of CA125 greater than, or equal to, two times the nadir (lowest) value on two occasions at least one week apart.

Elevated values must be confirmed by two separate measurements obtained at least one week apart. CA125 progression will be assigned the date of the first measurement that meets the criteria as noted.

17.11 Appendix 11 iRECIST Criteria

Overall response will be assessed using iRECIST (29). Immunotherapeutics may result in infiltration of immune cells leading to transient increase in the size in malignant lesions, resulting in previously undetectable lesions becoming detectable. The criteria are identical to those of RECIST 1.1 in many respects but have been adapted to account for instances where an increase in tumour burden, or the appearance of new lesions, does not reflect true tumour progression.

Key differences are described below. All responses defined using iRECIST criteria are designated with a prefix. iRECIST time-point and best overall responses will be recorded separately.

17.11.1 Confirming Progression

Unlike RECIST 1.1, iRECIST requires the confirmation of progression and uses the terms iUPD (unconfirmed progression) and iCPD (confirmed progression). Confirmatory scans should be performed at least 4 weeks, but no longer than 8 weeks after iUPD.

iCPD is confirmed if further progression, compared to the last assessment, is seen as evidenced by one or more of the following

- Continued progression where RECIST 1.1 definitions of progression had been met (from nadir) in target, non-target disease or new lesions (from iUPD)
    - Progression in target disease worsens with an increase of at least 5mm in the absolute value of the sum
    - Continued unequivocal progression in non-target disease with an increase in tumour burden
    - Increase in size of previously identified new lesion(s) (an increase of at least 5mm in the absolute value of the sum of those considered to be target new lesions)
- RECIST 1.1 criteria are met where progression was not previously identified, including the appearance of additional new lesions

If iUPD is not confirmed at the next assessment, then the appropriate response will be assigned (iUPD if the criteria are still met, but no worsening, or iSD, iPR or iCR if those criteria are met compared to baseline). As can be seen in Table 2, the prior documentation of iUPD does not preclude assigning iCR, iPR, or iSD in subsequent time-point assessments or as best overall response (BOR) providing that iCPD is not documented.

17.11.2 New Lesions (NL)

New lesions should be assessed and measured as they appear using RECIST 1.1 criteria (maximum of 5 lesions, no more than 2 per site, at least 10 mm in long axis (or 15 mm in short axis for nodal lesions), and recorded as New Lesions-Target (NLT) and New Lesion-Non-Target (NLNT) to allow clear differentiation from baseline target and non-target lesions.

New lesions may meet the criteria of NLT or NLNT to drive iUPD (or iCPD). However, the measurements of target lesions should NOT be included in the sum of measures of original target lesions identified at baseline. Rather, these measurements will be collected on a separate table in the case record form. These data will enable the development and testing of alternate response criteria, or further modifications of RECIST.

PD is confirmed in the New Lesion category if the next imaging assessment, conducted at least 4 weeks (but not more than 8 weeks) after iUPD confirms further progression from iUPD with either an increase of at least 5mm in the absolute value of the sum of NLT OR an increase (but not necessarily unequivocal increase) in the size of NLNT lesions OR the appearance of additional new lesions.

17.11.3 Time-point (TP) iResponse

<table>
<tr><th rowspan="2">Target Lesions*</th><th rowspan="2">Non-Target Lesions*</th><th rowspan="2">New Lesions*</th><th colspan="2">Time Point Response</th></tr>
<tr><th>No prior iUPD**</th><th>Prior iUPD**</th></tr>
<tr><td>CR / iCR</td><td>CR</td><td>No</td><td>CR</td><td>iCR</td></tr>
<tr><td>CR/iCR</td><td>Non-CR/Non-PD
Non-iCR/Non-iUPD</td><td>No</td><td>PR</td><td>iPR</td></tr>
<tr><td>PR/iPR</td><td>Non-CR/Non-PD
Non-iCR/Non-iUPD</td><td>No</td><td>PR</td><td>iPR</td></tr>
<tr><td>SD/iSD</td><td>Non-CR/Non-PD
Non-iCR/Non-iUPD</td><td>No</td><td>SD</td><td>iSD</td></tr>
<tr><td>iUPD with no change OR decrease from last TP</td><td>iUPD with no change OR decrease from last TP</td><td>Yes</td><td>NA</td><td>The appearance NLs confirms PD if
o Additional NLs or
o iUPD in last TP based on NLs and increase in size (≥ 5mm for NLT or any increase for NLNT)
If no change in NLs from last TP, remains iUPD</td></tr>
<tr><td>PD</td><td>Non-CR/Non-PD
Non-iCR/Non-iUPD</td><td>No</td><td>iUPD</td><td>Remains iUPD unless iCPD confirmed based on further increase in sum of at least 5mm, otherwise remains iUPD</td></tr>
<tr><td>PD</td><td>PD</td><td>No</td><td>iUPD</td><td>iCPD if further increase in previously identified***
T lesion iUPD ≥ 5mm and / or NT lesion iUPD</td></tr>
<tr><td>PD</td><td>PD</td><td>Yes</td><td>iUPD</td><td>iCPD if further increase in previously identified
T lesion iUPD ≥ 5mm and / or NT lesion iUPD and / or size or number of new lesion</td></tr>
<tr><td>Non-iUPD</td><td>Non-iUPD</td><td>Yes</td><td>iUPD</td><td>iCPD if increase in size of previously identified new lesions of increased number of new lesion</td></tr>
<tr><td colspan="5">* Using RECIST 1.1 principles. If no pseudoprogression occurs, RECIST 1.1 and iRECIST categories for CR, PR and SD would be the same ** in any category *** previously identified in assessment immediately prior to this time-point NA = not applicable</td></tr>
</table>

17.11.4 iRECIST Best Overall Response (iBOR)

All participants will have their iBOR from the start of study treatment until the end of treatment classified as outlined below.

**Time Point Response 1 (TPR1)**	**Time Point Response 2 (TPR2)**	**Time Point Response 3 (TPR3)**	**Time Point Response 4 (TPR4)**	**Time Point Response 5 (TPR5)**	**iRECIST Best Overall Response (iBOR)**
CR	CR, PR, iUPD, NE	CR/iCR, PR/iPR, iUPD, iCPD, NE	iUPD	iCPD	iCR
iUPD	PR, SD, NE	CR	CR, PR, SD, iUPD, NE	CR, PR, SD, iUPD, iCPD, NE	iCR
iUPD	PR	PR, SD, iUPD, NE	PR, SD, iUPD, NE, cPD	PR, SD, iUPD, NE, iCPD	iPR
iUPD	SD, NE	PR	PR, SD, iUPD, NE	PR, SD, iUPD, iCPD, NE	iPR
iUPD	SD	SD, iUPD, NE	SD, iUPD, iCPD, NE	SD, iUPD, iCPD, NE	iSD
iUPD	iUPD	Anything	Anything	Anything	iCPD
iUPD	iUPD	iCPD	Anything	Anything	iCPD
iUPD	NE	NE	NE	NE	iUPD

1) Table assumes a randomized study where confirmation of CR or PR is not required
2) NE = not evaluable that cycle
3) Designation "I" for BOR can be used to indicate prior uPD to aid in data interpretation
4) For participants with non-target disease only at baseline, only CR or non-CR/non-PD can be assigned at each TPR but is not shown in the table for ease of presentation

17.11.5 Response and Stable Disease Duration (RECIST 1.1 and iRECIST)

Response duration will be measured from the time measurement criteria for CR/PR or iCR/iPR (whichever is first recorded) are first met until the first date that recurrent or progressive disease is objectively documented, taking as reference the smallest measurements recorded on study (including baseline).

Stable disease duration will be measured from the time of start of treatment until the criteria for progression are met, taking as reference the smallest sum on study (including baseline).
