# Supplementary material for: Olaparib, durvalumab, and cyclophosphamide, and a prognostic blood signature in platinum-sensitive ovarian cancer: the randomized phase 2 SOLACE2 trial
Source: Nat Commun. 2025 Nov 5;16:9756. doi: 10.1038/s41467-025-64130-6 (PMC12589412; doi:10.1038/s41467-025-64130-6)
Supplement: Supplementary file 2 — Description of Additional Supplementary Files [file 41467_2025_64130_MOESM2_ESM.pdf]

Description of Additional Supplementary Files

Supplementary datasets of the SOLACE2 trial: Supplementary Dataset 1. Complete adverse events listing by treatment arm (Priming phase)

Supplementary datasets of the SOLACE2 trial: Supplementary Dataset 2. Complete adverse events listing by treatment arm (Consolidation phase)

Supplementary datasets of the SOLACE2 trial: Supplementary Dataset 3. Immune related adverse events by treatment arm.
