# Supplementary material for: Olaparib, durvalumab, and cyclophosphamide, and a prognostic blood signature in platinum-sensitive ovarian cancer: the randomized phase 2 SOLACE2 trial
Source: Nat Commun. 2025 Nov 5;16:9756. doi: 10.1038/s41467-025-64130-6 (PMC12589412; doi:10.1038/s41467-025-64130-6)
Supplement: Supplementary file 4 — Reporting Summary [file 41467_2025_64130_MOESM4_ESM.pdf]

Corresponding author(s): Chee Khoon Lee

Last updated by author(s): 7/17/2025

Reporting Summary

Nature Portfolio wishes to improve the reproducibility of the work that we publish. This form provides structure for consistency and transparency in reporting. For further information on Nature Portfolio policies, see our Editorial Policies and the Editorial Policy Checklist.

Statistics

For all statistical analyses, confirm that the following items are present in the figure legend, table legend, main text, or Methods section.

n/a	Confirmed	
☐	☒	The exact sample size (*n*) for each experimental group/condition, given as a discrete number and unit of measurement
☐	☒	A statement on whether measurements were taken from distinct samples or whether the same sample was measured repeatedly
☐	☒	The statistical test(s) used AND whether they are one- or two-sided<br>*Only common tests should be described solely by name; describe more complex techniques in the Methods section.*
☐	☒	A description of all covariates tested
☐	☒	A description of any assumptions or corrections, such as tests of normality and adjustment for multiple comparisons
☐	☒	A full description of the statistical parameters including central tendency (e.g. means) or other basic estimates (e.g. regression coefficient) AND variation (e.g. standard deviation) or associated estimates of uncertainty (e.g. confidence intervals)
☐	☒	For null hypothesis testing, the test statistic (e.g. *F*, *t*, *r*) with confidence intervals, effect sizes, degrees of freedom and *P* value noted<br>*Give P values as exact values whenever suitable.*
☒	☐	For Bayesian analysis, information on the choice of priors and Markov chain Monte Carlo settings
☒	☐	For hierarchical and complex designs, identification of the appropriate level for tests and full reporting of outcomes
☐	☒	Estimates of effect sizes (e.g. Cohen's *d*, Pearson's *r*), indicating how they were calculated

Our web collection on statistics for biologists contains articles on many of the points above.

Software and code

Policy information about availability of computer code

Data collection	No software was used as our work did not involve custom computer code or algorithm used to generate results that are reported in the paper and central to the study result.
Data analysis	Data analysis was undertaken using STATA version 15.1. the Swimmer's plot was developed using R version 2.2.2

For manuscripts utilizing custom algorithms or software that are central to the research but not yet described in published literature, software must be made available to editors and reviewers. We strongly encourage code deposition in a community repository (e.g. GitHub). See the Nature Portfolio guidelines for submitting code & software for further information.

Data

Policy information about availability of data

All manuscripts must include a data availability statement. This statement should provide the following information, where applicable:

- Accession codes, unique identifiers, or web links for publicly available datasets
- A description of any restrictions on data availability
- For clinical datasets or third party data, please ensure that the statement adheres to our policy

Deidentified study data are available for sharing. To request access to the deidentified study data, please contact the Corresponding Author. Requests will be reviewed by the Trial Management Committee and written applications from investigators with the academic capability and credibility to undertake the work proposed will be considered. The scientific merit of the proposal, including the appropriate methods, analysis, and publication plan will be assessed. Consideration will be taken of any overlap with analyses already undertaken or planned to be undertaken by the study team. If a proposal is approved, a signed data transfer

agreement will be required before data sharing.

Research involving human participants, their data, or biological material

Policy information about studies with human participants or human data. See also policy information about sex, gender (identity/presentation), and sexual orientation and race, ethnicity and racism.

Reporting on sex and gender	This study is about ovarian cancer is limited only to biological females with this disease
Reporting on race, ethnicity, or other socially relevant groupings	This study was a randomized trial. We did not restrict any patients based on their race, ethnicity, or other socioeconomic status. None of these variables were collected as part of this trial. As the study has randomized design, known and unknown confounders would be equally distributed between treatment arms.
Population characteristics	The study population is as outlined in the baseline demographic characteristics. The inclusion and exclusion criteria which defined the study population characteristics are stated in brief in the manuscript and also outlined in details in the study protocol.
Recruitment	Recruitment process is outlined in brief in the manuscript and more details outlined in the study protocol
Ethics oversight	Sydney Local Health District Human Research Ethics Committee (Approval number: X18-0123 & HREC/18/RPAH/169)

Note that full information on the approval of the study protocol must also be provided in the manuscript.

Field-specific reporting

Please select the one below that is the best fit for your research. If you are not sure, read the appropriate sections before making your selection.

- [x] Life sciences
- [ ] Behavioural & social sciences
- [ ] Ecological, evolutionary & environmental sciences

For a reference copy of the document with all sections, see nature.com/documents/nr-reporting-summary-flat.pdf

Life sciences study design

All studies must disclose on these points even when the disclosure is negative.

Sample size	Sample size is listed in the Methods section of the manuscript together with sample size assumptions
Data exclusions	Core inclusion and exclusion criteria are listed in the manuscript, together with a reference to Supplementary note 2 which contains the Trial Protocol with the complete inclusion and exclusion criteria. Data excluded from analyses has been outlined in the manuscript including the reason for its exclusion.
Replication	Replication was not undertaken as this is a Randomized Controlled Trial, and replication is not done within this trial design. Sufficient information regarding the trial design and conduct has been provided so that replication could be undertaken if desired.
Randomization	Radomization process described in brief, and stratification factors are also outlined. Detailed randomization process is listed in the study protocol provided as part of the submission
Blinding	This is an open-label unblinded study

Reporting for specific materials, systems and methods

We require information from authors about some types of materials, experimental systems and methods used in many studies. Here, indicate whether each material, system or method listed is relevant to your study. If you are not sure if a list item applies to your research, read the appropriate section before selecting a response.

Materials & experimental systems

n/a	Involved in the study	
[ ]	[x]	Antibodies
[x]	[ ]	Eukaryotic cell lines
[x]	[ ]	Palaeontology and archaeology
[x]	[ ]	Animals and other organisms
[ ]	[x]	Clinical data
[x]	[ ]	Dual use research of concern
[x]	[ ]	Plants

Methods

n/a	Involved in the study	
[x]	[ ]	ChIP-seq
[ ]	[x]	Flow cytometry
[x]	[ ]	MRI-based neuroimaging

Antibodies

Antibodies used	1. anti-CD3-AF700 (Manufacturer: Biolegend, Catalog No: 300424, Clone Name: UCHT1, Lot No: B317098, dilution used: 1:100)<br>2. anti-CD8-PerCP-Cy5.5 (Manufacturer: Biolegend, Catalog No: 560662, Clone Name: RPA-T8, Lot No: 309622, dilution used: 1:100)<br>3. anti-CD4-APC-Cy7 (Manufacturer: Biolegend, Catalog No: 300518, Clone Name: RPA-T4, Lot No: B314214, dilution used: 1:100)<br>4. anti-CD25-PE-Dazzle594 (Manufacturer: Biolegend, Catalog No: 356126, Clone Name: M-A251, Lot No: B327560, dilution used: 1:50)<br>5. anti-CCR4-BV421 (Manufacturer: Biolegend, Catalog No: 359414, Clone Name: L291H4, Lot No: B381005, dilution used: 1:50)<br>6. anti-FoxP3-APC (Manufacturer: Invitrogen, Catalog No: 17-4776-42, Clone Name: PCH101, Lot No: 2146550, dilution used: 1:40)<br>7. anti-CD127-BV650 (Manufacturer: Biolegend, Catalog No: 351326, Clone Name: A019D5, Lot No: B329912, dilution used: 1:100)
Validation	1. CD3-AF700 Species Reactivity Verified: Human, Application: Quality validated for flow cytometry, Manufacturer's website: https://www.biolegend.com/en-gb/products/alexa-fluor-700-anti-human-cd3-antibody-3394?GroupID=BLG5900<br>2. CD8-PerCP-Cy5.5 Species Reactivity Verified: Human, Application: Quality validated for flow cytometry, Manufacturer's website: https://www.biolegend.com/en-gb/products/percp-cyanine5-5-anti-human-cd8-20764<br>3. CD4-APC-Cy7 Species Reactivity Verified: Human, Application: Quality validated for flow cytometry, Manufacturer's website: https://www.biolegend.com/en-gb/products/apc-cyanine7-anti-human-cd4-antibody-1933?GroupID=BLG5901<br>4. CD25-PE-Dazzle594 Species Reactivity Verified: Human, Application: Quality validated for flow cytometry, Manufacturer's website: https://www.biolegend.com/ja-jp/products/pe-dazzle-594-anti-human-cd25-antibody-9782<br>5. CCR4-BV421 Species Reactivity Verified: Human, Application: Quality validated for flow cytometry, Manufacturer's website: https://www.biolegend.com/fr-ch/search-results/brilliant-violet-421-anti-human-cd194-ccr4-antibody-8790<br>6. FoxP3-APC Species Reactivity Verified: Human, Application: Quality validated for flow cytometry, Manufacturer's website: https://www.thermofisher.com/antibody/product/FOXP3-Antibody-clone-PCH101-Monoclonal/17-4776-42<br>7. CD127-BV650 Species Reactivity Verified: Human, Application: Quality validated for flow cytometry, Manufacturer's website: https://www.biolegend.com/fr-ch/products/brilliant-violet-650-anti-human-cd127-il-7ralpha-antibody-7673

Clinical data

Policy information about clinical studies

All manuscripts should comply with the ICMJEguidelines for publication of clinical research and a completedCONSORT checklist must be included with all submissions.

Clinical trial registration	Australian New Zealand Clinical Trials Registry ACTRN12618000686202
Study protocol	The Trial Protocol is included with the manuscript in Supplementary note 2. The protocol was approved by Sydney Local Health District Human Research Ethics Committee - RPAH Zone.
Data collection	Data collection process are outlined in brief of the manuscript and also in greater details in the study protocol
Outcomes	Pre-defined outcome measures are outlined in the manuscript and also study protocol

Plants

Seed stocks	N/A
Novel plant genotypes	N/A
Authentication	N/A

Flow Cytometry

Plots

Confirm that:

- [x] The axis labels state the marker and fluorochrome used (e.g. CD4-FITC).
- [x] The axis scales are clearly visible. Include numbers along axes only for bottom left plot of group (a 'group' is an analysis of identical markers).
- [x] All plots are contour plots with outliers or pseudocolor plots.
- [x] A numerical value for number of cells or percentage (with statistics) is provided.

Methodology

Sample preparation	Peripheral blood mononuclear cells (PBMCs) were isolated within 24 hours of blood collection using Ficoll density gradient centrifugation. After red blood cell lysis, the PBMCs were washed and collected. They were then cryopreserved in 90% human serum and 10% DMSO. For flow cytometry analysis, cryopreserved PBMCs from trial participants and healthy adult female donors from the Australian Red Cross Blood Service were thawed and cultured in complete AIM-V media, either alone or with mafosphamide.
Instrument	ZE-5 cell analyzer (Bio-rad, USA)
Software	Everest software (Bio-rad, USA) for flow cytometry and FlowJo software (TreeStar, USA) for analysis.
Cell population abundance	The CCR4+ cells were determined with the use of fluorescence minus one controls that enable accurate gating.
Gating strategy	Gating strategy is provided in the supplementary figure, with the following sequence: single cells, then live cells, then lymphocytes, then CD3+, then CD8+ continued with CD8+CCR4+, or CD4+CD25- continued with CD4+CD25-CCR4+, or Treg continued with TregCCR4+

- [x] Tick this box to confirm that a figure exemplifying the gating strategy is provided in the Supplementary Information.
